# Design, Synthesis and Biological Evaluation of Novel Oleanolic Acid Derivatives as Potential Anti-Leukemic Agents

**DOI:** 10.3390/life16081335

**Published:** 2026-08-14

**Authors:** Nefise Demir, Halilibrahim Ciftci, Khaled M. Elamin, Mustafa Can, Belgin Sever

**Affiliations:** 1 Department of Nanoscience and Nanotechnology, Izmir Kâtip Celebi University, Izmir 35620, Türkiye; demir.nefise53@gmail.com; 2 Department of Molecular Biology and Genetics, Burdur Mehmet Akif Ersoy University, Istiklal Campus, Burdur 15200, Türkiye; hciftci@mehmetakif.edu.tr; 3 Medicinal and Biological Chemistry Science Farm Joint Research Laboratory, Faculty of Life Sciences, Kumamoto University, Kumamoto 862-0973, Japan; 4 Department of Drug Discovery, Science Farm Ltd., Kumamoto 862-0976, Japan; 5 Global Center for Natural Products Research, Faculty of Life Sciences, Kumamoto University, 5-1 Oe-honmachi, Chuo-ku, Kumamoto 862-0973, Japan; khaled@kumamoto-u.ac.jp; 6 Department of Bioengineering Sciences, Faculty of Engineering and Architecture, Izmir Kâtip Celebi University, Izmir 35620, Türkiye; 7 Department of Pharmaceutical Chemistry, Faculty of Pharmacy, Anadolu University, Eskisehir 26470, Türkiye

**Keywords:** leukemia, oleanolic acid derivatives, apoptosis, targeted drug design

## Abstract

**Background:** Chronic myeloid leukemia (CML) and other leukemia subtypes remain significant hematological malignancies that require the development of novel therapeutic agents with improved efficacy and selectivity. This study aimed to design, synthesize, and evaluate novel oleanolic acid (OA) derivatives as potential anti-leukemic agents. **Methods:** Structural modifications at the C-28 position afforded halogenated benzyl ester derivatives (MT-A–D), while aromatic acyl substitutions at the C-3 position generated derivatives NOA-1–16. The cytotoxic effects of the synthesized compounds were evaluated against K562, HL-60, Jurkat, and MT-2 leukemia cell lines and healthy PBMCs using the MTT assay. Apoptosis induction and ABL tyrosine kinase (TK) inhibition were also investigated. Molecular docking, MM/GBSA binding free energy calculations, and molecular dynamics (MD) simulations were performed to characterize ligand-enzyme interactions and binding stability. **Results:** MT-A exhibited the highest activity against HL-60 cells (IC_50_ = 4.17 ± 0.65 μM), surpassing imatinib (IC_50_ = 13.34 ± 1.92 μM). NOA-8 was the most potent compound against Jurkat and MT-2 cells, with IC_50_ values of 6.81 ± 1.08 μM and 2.48 ± 0.88 μM, respectively. MT-A, MT-C, NOA-3, and NOA-8 displayed IC_50_ values above 300 μM in PBMCs, indicating favorable selectivity. MT-A and MT-C induced significant apoptosis in K562 cells, while kinase assays revealed moderate ABL TK inhibition by these compounds. Docking, MM/GBSA, and MD analyses supported stable binding of MT-A and MT-C within the ABL ATP-binding pocket. **Conclusions:** MT-A, MT-C, NOA-3, and particularly NOA-8 emerged as promising anti-leukemic lead compounds with potent antiproliferative activity and favorable selectivity profiles, warranting further investigation for leukemia therapy.

## 1. Introduction

Leukemia is generally categorized into two principal groups, namely lymphoid and myeloid leukemias. Lymphoid malignancies include acute lymphoblastic leukemia (ALL) and chronic lymphocytic leukemia (CLL), which originate from lymphoid precursor cells that subsequently develop into T and B lymphocytes involved in immune responses. In contrast, acute myeloid leukemia (AML) and chronic myeloid leukemia (CML) arise from myeloid progenitor cells responsible for the generation of erythrocytes, platelets, and several classes of leukocytes [1,2].

The identification of new therapeutic agents and complementary treatment strategies remains an important objective in CML research. Natural products derived from plants have long been recognized as valuable sources of anticancer agents due to their structural diversity and multi-target biological activities. Many plant-derived compounds exhibit significant anticancer effects with relatively low toxicity and fewer side effects compared with synthetic drugs, making them promising candidates for long-term therapeutic applications [3]. Several natural compounds have demonstrated potential anti-CML activity. Emodin, a natural anthraquinone derivative isolated from *Rheum palmatum* L., has been reported to exhibit antibacterial, anti-inflammatory, and anticancer activities. Studies have shown that emodin induces apoptosis in K562 leukemia cells both in vitro and in vivo, indicating its potential as a low-toxicity anticancer agent [4]. Furthermore, emodin has been shown to sensitize imatinib-resistant K562/G01 cells to imatinib treatment by promoting apoptosis [5]. Similarly, oridonin, a diterpenoid compound isolated from *Isodon rubescens*, has demonstrated promising antileukemic activity [6]. In addition, berberine, a plant-derived alkaloid, has been reported to induce autophagic degradation in imatinib-resistant leukemia cells, thereby suppressing the development of drug-resistant mutations [7]. Curcumin has also been shown to exert cytotoxic effects against CML cell lines by inducing apoptosis, causing cell-cycle arrest, and inhibiting several oncogenic signaling pathways [8]. Resveratrol, on the other hand, has been reported to inhibit HSF1 transcriptional activity in K562 CML cells, leading to reduced Hsp70 levels and enhanced apoptosis, thereby potentiating chemotherapeutic efficacy [9].

Among plant-derived bioactive molecules, pentacyclic triterpenoids represent an important class of secondary metabolites widely distributed in nature. These compounds possess a 30-carbon skeleton composed of five interconnected rings and have attracted considerable attention due to their diverse biological activities, including antiviral, anti-inflammatory, antioxidant, and anticancer effects [10]. Based on their structural features, triterpenoids are commonly classified into subclasses such as oleanane, ursane, and lupane. However, despite their promising pharmacological activities, pentacyclic triterpenoids often exhibit poor aqueous solubility and limited pharmacokinetic properties, which restrict their clinical applications. Therefore, the development of semisynthetic derivatives has become an effective strategy to improve their pharmacological properties, including bioavailability, efficacy, and target specificity [11]. In this regard, compounds such as oleanolic acid (OA), ursolic acid, and betulinic acid have shown potent antitumor activities in various cancers while exhibiting selective toxicity toward cancer cells and minimal effects on normal cells [12]. In a previous study, benzyl ester derivatives of several pentacyclic triterpenoids, including asiatic acid, betulinic acid, glycyrrhetinic acid, hederagenin, OA, ursolic acid, and gypsogenin were synthesized and evaluated for their anticancer activities. These derivatization studies demonstrated enhanced inhibitory activity against ABL tyrosine kinase (TK) and CML cells, with the most potent activity observed for derivatives based on the OA scaffold [13]. OA and its derivatives possess significant anticancer potential, and their chemical modification and formulation-based optimization provide promising opportunities to enhance efficacy, improve bioavailability, and support targeted therapeutic applications [14].

OA is a naturally occurring triterpenoid found in fruits and medicinal plants and is known for its broad range of biological activities. Structural modifications of OA can enhance its biological efficacy, and several OA derivatives have been reported to exhibit greater cytotoxicity than the parent compound [15]. In recent years, significant progress has been made in triterpenoid research, particularly in the development of synthetic OA derivatives. CDDO and its derivatives, including CDDO-Me and CDDO-Im, have demonstrated potent anti-inflammatory and antitumor effects. In addition, di-CDDO and various amide derivatives, such as CDDO-MA, CDDO-EA, and CDDO-TFEA, exhibit antitumor properties by affecting multiple intracellular processes, including inhibition of tumor cell proliferation, induction of apoptosis, and suppression of pro-inflammatory cytokine signaling [16]. OA has also been reported to induce apoptosis in human leukemia cells through caspase activation and cleavage of poly(ADP-ribose) polymerase (PARP), highlighting its potential as an anticancer agent [17]. Furthermore, previous studies have shown that structurally modified OA derivatives can exhibit potent antiproliferative effects and induce apoptosis in the human K562 CML cell line [18].

Although numerous OA derivatives have been developed through structural modification, further optimization of their biological activities and a deeper understanding of their structure-activity relationship (SAR) remain important objectives in OA-based drug discovery. Recent reviews have highlighted that the C-3 hydroxyl group and the C-28 carboxyl group represent the principal modification sites of OA, and structural alterations at these positions can markedly influence the pharmacological properties and biological activities of the resulting derivatives [19,20]. Based on these considerations, the substituents employed in the present study were selected according to a rational molecular design strategy. Halogen-substituted benzyl groups were introduced at the C-28 position to preserve the benzyl ester scaffold while providing structural diversity. The aromatic benzyl moiety was selected to potentially facilitate aromatic (π-related) interactions, whereas the halogen substituents (F, Cl, and I) were incorporated to modulate the electronic properties of the molecules and potentially promote favorable non-covalent interactions with amino acid residues. Halogen substitution is a well-established strategy in medicinal chemistry because it can influence molecular recognition, binding affinity, and physicochemical properties through electronic effects and halogen-associated non-covalent interactions [21,22]. In addition, aromatic acyl substituents were introduced at the C-3 position to investigate the influence of C-3 structural modification on the biological activity of the synthesized derivatives, as modifications at this position have been reported to significantly affect the biological properties of OA derivatives [19,20].

Building on these observations, the present study describes the rational design, synthesis, and comprehensive biological evaluation of a series of C-28 benzyl ester and C-3 aromatic acyl derivatives of OA. Their anti-leukemic potential was investigated through cytotoxicity, apoptosis, ABL TK inhibition, and complementary computational studies.

## 2. Materials and Methods

### 2.1. Chemistry

Melting points (m.p.) of all compounds were determined using a Gallenkamp electrothermal melting point apparatus and are uncorrected (±0.1 °C). ^1^H NMR (600 and 500 MHz) and ^13^C NMR spectra were recorded on a Bruker spectrometer (Bruker Avance III HD and Bruker, Billerica, MA, USA). Column chromatography was performed using silica gel (60 Å, Merck 7734, Merck KGaA, Darmstadt, Germany), while thin-layer chromatography (TLC) analyses were carried out on silica gel 60 Å F254 aluminum plates (Merck 5554). High-resolution mass spectrometry (HRMS) analyses were conducted using an Agilent 6530 Accurate-Mass Q-TOF LC/MS instrument (Agilent Technologies, Santa Clara, CA, USA), whereas LC/MS measurements were performed on a JEOL JMS-700 Station/JMS-BU-20-GCmate system (JEOL, Akishima, Tokyo, Japan).

#### 2.1.1. Synthesis of MT-A-D

OA benzyl ester derivatives were synthesized via esterification of the C-28 carboxylic acid using substituted benzyl bromides in the presence of K_2_CO_3_ in dimethylformamide (DMF), following a previously reported method [23]. Briefly, OA (1 eq) was dissolved in anhydrous DMF (5 mL), and K_2_CO_3_ (1.5 eq) was added. The reaction mixture was stirred at room temperature (r.t.) for 30 min, followed by the addition of the appropriate substituted benzyl bromide derivatives (1.2 eq). The reaction was continued at r.t. for 6 h and monitored by TLC. Upon completion, brine was added, and the mixture was extracted with ethyl acetate. The combined organic layers were washed with water until neutral pH, dried over anhydrous MgSO_4_, filtered, and concentrated under reduced pressure. The crude product was purified by column chromatography using appropriate eluents to afford the desired benzyl ester derivatives.

4-Chlorobenzyl (4aS,6aS,6bR,8aR,10S,12aR,12bR,14bS)-10-hydroxy-2,2,6a,6b,9,9,12a-heptamethyl-1,3,4,5,6,6a,6b,7,8,8a,9,10,11,12,12a,12b,13,14b-octadecahydropicene-4a(2H)-carboxylate (MT-A)

Yield: 60%. M.p. 178–179 °C. ^1^H NMR (500 MHz, CDCl_3_): 0.57 (s, 3H), 0.78 (s, 4H), 0.88–1.05 (m, 14H), 1.13–1.24 (m, 6H), 1.31–1.42 (m, 3H), 1.48–1.72 (m, 11H), 1.84 (dd, *J* = 2.5, 3.1 Hz, 2H), 1.98 (dt, *J* = 6.9, 4.9 Hz, 1H), 2.88 (dd, *J* = 4.9, 5.6 Hz, 1H), 3.20 (t, *J* = 4.9 Hz, 1H), 5.03 (d, *J* = 12.4, 1H), 5.20 (d, *J* = 13.0 Hz, 1H), 5.27 (t, *J* = 3.7 Hz, 1H), 7.27 (d, *J* = 10.5 Hz, 2H), 7.32 (d, *J* = 8.7 Hz, 2H). ^13^C NMR (125 MHz, DMSO-*d_6_*): 15.3 (CH_3_), 15.6 (CH_3_), 17.1 (CH_3_), 18.5 (CH_3_), 22.9 (CH_3_), 23.4 (CH_3_), 23.7 (CH_3_), 26.0 (CH_2_), 27.2 (CH_2_), 27.8 (CH_2_), 28.3 (CH_2_), 30.7 (CH_2_), 32.5 (CH_2_), 32.8 (CH_2_), 33.2 (CH_2_), 34.0 (CH_2_), 37.1 (CH_2_), 38.5 (CH), 38.7 (CH), 39.2 (CH), 41.3 (CH), 41.7 (C), 45.7 (C), 46.9 (C), 47.7 (C), 55.2 (C), 65.2 (CH_2_), 79.1 (C), 122.8 (CH), 128.5 (2CH), 129.6 (2CH), 135.1 (2C), 143.7 (C), 177.3 (C). HRMS (ESI) calcd. for C_37_H_53_ClO_3_ [M + H]^+^: m/z = 580.36832; found: 580.36686. (Spectral Data: Supplementary Information Figures S1–S3).

4-Iodobenzyl (4aS,6aS,6bR,8aR,10S,12aR,12bR,14bS)-10-hydroxy-2,2,6a,6b,9,9,12a-heptamethyl-1,3,4,5,6,6a,6b,7,8,8a,9,10,11,12,12a,12b,13,14b-octadecahydropicene-4a(2H)-carboxylate (MT-B)

Yield: 55%. M.p. 170–171 °C. ^1^H NMR (500 MHz, CDCl_3_): 0.54 (s, 3H), 0.78 (s, 4H), 0.89–1.04 (m, 14H), 1.12–1.24 (m, 6H), 1.28–1.44 (m, 4H), 1.48–1.72 (m, 10H), 1.84 (dd, *J* = 3.5, 3.9 Hz, 2H), 1.97 (dt, *J* = 3.2, 3.7, 4.6 Hz, 1H), 2.87 (dd, *J* = 3.2, 4.6 Hz, 1H), 3.18–3.20 (m, 1H), 4.96 (d, *J* = 13.0 Hz, 1H), 5.03 (d, *J* = 11.0 Hz, 1H), 5.27 (t, *J* = 2.8 Hz, 1H), 7.09 (d, *J* = 8.7 Hz, 2H), 7.67 (d, *J* = 9.6 Hz, 2H). ^13^C NMR (125 MHz, DMSO-*d_6_*): 15.4 (CH_3_), 15.6 (CH_3_), 16.8 (CH_3_), 18.3 (CH_3_), 23.1 (CH_3_), 23.4 (CH_3_), 23.6 (CH_3_), 25.9 (CH_2_), 27.2 (CH_2_), 27.6 (CH_2_), 28.2 (CH_2_), 30.8 (CH_2_), 32.4 (CH_2_), 32.8 (CH_2_), 33.9 (CH_2_), 33.9 (CH_2_), 37.1 (CH_2_), 38.5 (CH), 38.8 (CH), 39.3 (CH), 41.4 (CH), 41.7 (C), 45.9 (C), 46.8 (C), 47.7 (C), 55.3 (C), 65.3 (CH_2_), 78.9 (C), 93.7 (C), 122.6 (CH), 130.2 (2CH), 136.2 (C), 137.6 (2CH), 143.7 (C), 177.4 (C). HRMS (ESI) calcd. for C_37_H_53_lO_3_ [M + H]^+^: m/z = 672.30394; found: 672.30256. (Spectral Data: Supplementary Information Figures S4–S6).

4-Chloro-3-fluorobenzyl (4aS,6aS,6bR,8aR,10S,12aR,12bR,14bS)-10-hydroxy-2,2,6a,6b,9,9,12a-heptamethyl-1,3,4,5,6,6a,6b,7,8,8a,9,10,11,12,12a,12b,13,14b-octadecahydropicene-4a(2H)-carboxylate (MT-C)

Yield: 45%. M.p. 176–177 °C. ^1^H NMR (500 MHz, CDCl_3_): 0.55 (s, 3H), 0.77 (s, 4H), 0.88–1.07 (m, 11H), 1.13–1.43 (m, 12H), 1.46–1.74 (m, 11H), 1.85 (dd, *J* = 3.2, 4.1 Hz, 2H), 1.99 (dt, *J* = 3.8 Hz, 1H), 2.88 (dd, *J* = 4.6, 4.9 Hz, 1H), 3.20 (dd, *J* = 3.8, 5.2 Hz, 1H), 4.96 (d, *J* = 13.0 Hz, 1H), 5.08 (d, *J* = 12.7 Hz, 1H), 5.29 (t, *J* = 2.9 Hz, 1H), 7.05–7.16 (m, 2H), 7.36 (t, *J* = 7.4 Hz, 1H). ^13^C NMR (125 MHz, DMSO-*d_6_*): 15.3 (CH_3_), 15.6 (CH_3_), 17.0 (CH_3_), 18.4 (CH_3_), 23.1 (CH_3_), 23.4 (CH_3_), 23.8 (CH_3_), 25.9 (CH_2_), 27.2 (CH_2_), 27.6 (CH_2_), 28.2 (CH_2_), 30.8 (CH_2_), 32.5 (CH_2_), 32.8 (CH_2_), 33.1 (CH_2_), 33.9 (CH_2_), 37.0 (CH_2_), 38.5 (CH), 38.8 (CH), 39.4 (CH), 41.5 (CH), 41.8 (C), 45.9 (C), 46.9 (C), 47.6 (C), 55.3 (C), 64.6 (CH_2_), 79.0 (C), 116.2 (CH), 116.3 (C), 122.7 (CH), 124.4 (CH), 130.7 (CH), 136.2 (C), 137.4 (C), 143.6 (C), 177.3 (C). HRMS (ESI) calcd. for C_37_H_52_ClFO_3_ [M + H]^+^: m/z = 598.3589; found: 598.35694. (Spectral Data: Supplementary Information Figures S7–S9).

3,4-Dichlorobenzyl (4aS,6aS,6bR,8aR,10S,12aR,12bR,14bS)-10-hydroxy-2,2,6a,6b,9,9,12a-heptamethyl-1,3,4,5,6,6a,6b,7,8,8a,9,10,11,12,12a,12b,13,14b-octadecahydropicene-4a(2H)-carboxylate (MT-D)

Yield: 50%. M.p. 169–170 °C. ^1^H NMR (500 MHz, CDCl_3_): 0.53 (s, 3H), 0.71 (d, *J* = 13.9 Hz, 1H), 0.78 (s, 3H), 0.88 (s, 3H), 0.90 (s, 3H), 0.92 (s, 3H), 0.98 (s, 3H), 1.05 (d, *J* = 17.9 Hz, 1H), 1.13 (s, 3H), 1.17–1.25 (m, 3H), 1.32–1.44 (m, 4H), 1.49–1.74 (m, 11H), 1.85 (dd, *J* = 3.3, 8.8 Hz, 2H), 1.99 (td, *J* = 3.6, 4.1, 4.3 Hz, 1H), 2.88 (dd, *J* = 3.9, 13.9 Hz, 1H), 3.20 (dd, *J* = 4.8, 11.7 Hz, 1H), 4.94 (d, *J* = 13.2 Hz, 1H), 5.08 (d, *J* = 12.1 Hz, 1H), 5.28 (s, 1H), 7.16–7.18 (m, 1H), 7.40 and 7.42 (2s, 1H), 7.45 (s, 1H). ^13^C NMR (125 MHz, CDCl_3_): 15.3 (CH_3_), 15.6 (CH_3_), 16.8 (CH_3_), 18.3 (CH_3_), 23.1 (CH_3_), 23.4 (CH_3_), 23.7 (CH_3_), 25.9 (CH_2_), 27.3 (CH_2_), 27.7 (CH_2_), 28.2 (CH_2_), 30.7 (CH_2_), 32.4 (CH_2_), 32.7 (CH_2_), 33.1 (CH_2_), 33.9 (CH_2_), 37.1 (CH_2_), 38.4 (CH), 38.7 (CH), 39.5 (CH), 41.4 (CH), 41.7 (C), 45.8 (C), 46.9 (C), 47.3 (C), 47.6 (C), 55.2 (C), 64.4 (CH_2_), 78.9 (C), 122.7 (CH), 127.4 (CH), 130.2 (CH), 130.4 (CH), 132.1 (C), 135.6 (C), 143.7 (C), 177.5 (C). HRMS (ESI) calcd. for C_37_H_52_Cl_2_O_3_ [M + H]^+^: m/z = 614.32935; found: 614.3317. (Spectral Data: Supplementary Information Figures S10–S12).

#### 2.1.2. Synthesis of NOA1-16

To a solution of the carboxylic acid-containing compound (1.0 eq) in dichloromethane (DCM), MT-A-D (4.1 eq), 1-ethyl-3-(3-dimethylaminopropyl)carbodiimide (EDCI) (5.3 eq), and dimethylaminopyridine (DMAP) (0.6 eq) were added. The reaction mixture was stirred under reflux in DCM for 4 h and monitored by TLC until completion. The solvent was removed under reduced pressure, and the crude product was purified by silica gel column chromatography (hexane/ethyl acetate, 20:1) to afford the desired NOA-1-16 derivatives [24].

The isolated yields of the synthesized NOA derivatives were generally moderate, ranging from 35% to 47%. No clear relationship was observed between the electronic nature of the aromatic acyl substituents and the isolated yields, suggesting that electronic effects were not the primary factor governing the reaction efficiency under the applied EDCI/DMAP coupling conditions. Instead, moderate yields are more likely attributable to the sterically congested environment surrounding the C-3 hydroxyl group of OA, which may reduce the efficiency of esterification. In addition, partial product losses during silica gel column chromatography may also have contributed to the moderate isolated yields.

4-Chlorobenzyl (4aS,6aS,6bR,8aR,10S,12aR,12bR,14bS)-10-((2,4-dimethoxybenzoyl)oxy)-2,2,6a,6b,9,9,12a-heptamethyl-1,3,4,5,6,6a,6b,7,8,8a,9,10,11,12,12a,12b,13,14b-octadecahydropicene-4a(2H)-carboxylate (NOA-1)

Yield: 44%. M.p. 144–145 °C. ^1^H NMR (600 MHz, CDCl_3_): 0.57 (d, *J* = 9.2 Hz, 3H), 0.78 (s, 1H), 0.87–0.98 (m, 14H), 1.02–1.09 (m, 2H), 1.12 (s, 1H), 1.14 (s, 2H), 1.16–1.25 (m, 4H), 1.31–1.49 (m, 4H), 1.50–1.72 (m, 8H), 1.77–1.80 (m, 1H), 1.83–1.87 (m, 2H), 1.95–2.01 (m, 1H), 2.88 (dd, *J* = 3.2, 13.5 Hz, 1H), 3.84 (s, 3H), 3.88 (s, 3H), 4.69 (dd, *J* = 4.7, 11.7 Hz, 1H), 4.99 (d, *J* = 13.6 Hz, 1H), 5.07 (d, *J* = 11.0 Hz, 1H), 5.28 (s, 1H), 6.47–6.50 (m, 2H), 7.27–7.33 (m, 4H), 7.86 (d, *J* = 7.7 Hz, 1H). ^13^C NMR (125 MHz, CDCl_3_): 15.4 (CH_3_), 15.6 (CH_3_), 16.7 (CH_3_), 16.9 (CH_3_), 17.1 (CH_3_), 18.2 (CH_3_), 23.0 (CH_3_), 23.4 (CH_2_), 23.6 (CH_2_), 25.9 (CH_2_), 27.6 (CH_2_), 30.7 (CH_2_), 32.3 (CH_2_), 32.7 (CH_2_), 33.1 (CH_2_), 33.8 (CH_2_), 36.9 (CH_2_), 38.0 (CH), 38.2 (C), 39.4 (C), 41.4 (C), 41.7 (C), 45.8 (C), 46.8 (C), 47.6 (C), 55.3 (CH), 55.5 (CH_3_), 55.9 (CH_3_), 65.2 (CH_2_), 80.9 (CH), 98.9 (CH), 104.4 (CH), 113.1 (C), 122.5 (CH), 128.5 (2CH), 129.6 (2CH), 133.7 (2C), 134.8 (CH), 143.6 (C), 161.4 (C), 164.2 (C), 165.4 (C), 177.5 (C). HRMS (ESI) calcd. for C_46_H_61_ClO_6_ [M + H]^+^: m/z = 744,41567; found: 744,41594. (Spectral Data: Supplementary Information Figures S13–S15).

4-Chlorobenzyl (4aS,6aS,6bR,8aR,10S,12aR,12bR,14bS)-2,2,6a,6b,9,9,12a-heptamethyl-10-((3,4,5-trimethoxybenzoyl)oxy)-1,3,4,5,6,6a,6b,7,8,8a,9,10,11,12,12a,12b,13,14b-octadecahydropicene-4a(2H)-carboxylate (NOA-2)

Yield: 42%. M.p. 154–155 °C. ^1^H NMR (600 MHz, CDCl_3_): 0.58 (s, 3H), 0.91 (s, 3H), 0.92 (s, 3H), 0.94 (s, 3H), 0.96 (s, 3H), 1.00 (s, 3H), 1.03–1.07 (m, 1H), 1.14 (s, 3H), 1.17–1.27 (m, 3H), 1.32–1.49 (m, 4H), 1.52–1.60 (m, 5H), 1.63–1.68 (m, 4H), 1.70–1.77 (m, 2H), 1.86–1.88 (m, 2H), 1.98 (td, *J* = 4.1, 4.7 Hz, 1H), 2.89 (dd, *J* = 4.9, 14.0 Hz, 1H), 3.91 (s, 9H), 4.72 (dd, *J* = 6.1, 10.4 Hz, 1H), 4.99 (d, *J* = 12.7 Hz, 1H), 5.07 (d, *J* = 12.6 Hz, 1H), 5.29 (s, 1H), 7.27–7.33 (m, 6H). ^13^C NMR (125 MHz, CDCl_3_): 15.4 (CH_3_), 16.8 (CH_3_), 17.1 (CH_3_), 18.2 (CH_3_), 23.1 (CH_3_), 23.5 (CH_3_), 23.7 (CH_3_), 25.9 (CH_2_), 26.8 (CH_2_), 27.6 (CH_2_), 28.3 (CH_2_), 30.8 (CH_2_), 32.3 (CH_2_), 32.7 (CH_2_), 33.1 (CH_2_), 33.8 (CH_2_), 36.9 (CH_2_), 38.1 (2CH), 39.3 (C), 41.5 (C), 45.8 (C), 46.7 (C), 47.6 (C), 55.4 (CH), 56.2 (3CH_3_), 60.9 (C), 65.1 (CH_2_), 81.7 (CH), 106.9 (2CH), 122.5 (CH), 126.0 (C), 128.7 (2CH), 129.7 (2CH), 133.9 (C), 134.8 (C), 142.2 (C), 143.9 (C), 152.9 (2C), 165.7 (C), 177.6 (C). HRMS (ESI) calcd. for C_47_H_63_ClO_7_ [M + H]^+^: m/z = 774.42623; found: 774.4278. (Spectral Data: Supplementary Information Figures S16–S18).

4-Chlorobenzyl (4aS,6aS,6bR,8aR,10S,12aR,12bR,14bS)-10-(benzoyloxy)-2,2,6a,6b,9,9,12a-heptamethyl-1,3,4,5,6,6a,6b,7,8,8a,9,10,11,12,12a,12b,13,14b-octadecahydropicene-4a(2H)-carboxylate (NOA-3)

Yield: 44%. M.p. 136–137 °C. ^1^H NMR (600 MHz, CDCl_3_): 0.58 (s, 3H), 0.91 (s, 3H), 0.92 (s, 3H), 0.94 (s, 3H), 0.96 (s, 3H), 1.02 (s, 3H), 1.04–1.12 (m, 2H), 1.14 (s, 3H), 1.17–1.26 (m, 3H), 1.32–1.49 (m, 3H), 1.52–1.60 (m, 5H), 1.65 (t, *J* = 13.3 Hz, 3H), 1.69–1.79 (m, 3H), 1.86–1.88 (m, 2H), 1.98 (td, *J* = 3.7, 4.1, 4.4 Hz, 1H), 2.89 (dd, *J* = 4.1, 14.2 Hz, 1H), 4.73 (dd, *J* = 4.9, 11.1 Hz, 1H), 4.99 (d, *J* = 12.7 Hz, 1H), 5.07 (d, *J* = 12.4 Hz, 1H), 5.29 (s, 1H), 7.27–7.33 (m, 4H), 7.44 (t, *J* = 7.6 Hz, 2H), 7.55 (t, *J* = 6.9 Hz, 1H), 8.04 (d, *J* = 7.4 Hz, 2H). ^13^C NMR (125 MHz, CDCl_3_): 15.4 (CH_3_), 16.8 (CH_3_), 16.9 (CH_3_), 18.2 (CH_3_), 23.1 (CH_3_), 23.4 (CH_3_), 23.6 (CH_3_), 25.9 (CH_2_), 27.6 (CH_2_), 28.2 (CH_2_), 30.7 (2CH_2_), 32.4 (CH_2_), 32.7 (CH_2_), 33.1 (CH_2_), 33.8 (CH_2_), 36.9 (CH_2_), 38.1 (CH), 38.2 (2CH), 39.3 (C), 41.4 (C), 45.8 (2C), 46.8 (C), 47.5 (C), 55.4 (CH), 65.1 (CH_2_), 81.6 (CH), 122.5 (CH), 128.3 (2CH), 128.6 (2CH), 129.5 (CH), 129.6 (2CH), 130.9 (CH), 132.7 (C), 133.9 (C), 134.9 (C), 143.7 (C), 166.3 (C), 177.4 (C). HRMS (ESI) calcd. for C_44_H_57_ClO_4_ [M + H]^+^: m/z = 684.39454; found: 684.39379. (Spectral Data: Supplementary Information Figures S19–S21).

4-Chlorobenzyl (4aS,6aS,6bR,8aR,10S,12aR,12bR,14bS)-10-((1-naphthoyl)oxy)-2,2,6a,6b,9,9,12a-heptamethyl-1,3,4,5,6,6a,6b,7,8,8a,9,10,11,12,12a,12b,13,14b-octadecahydropicene-4a(2H)-carboxylate (NOA-4)

Yield: 40%. M.p. 156–157 °C. ^1^H NMR (600 MHz, CDCl_3_): 0.59 (s, 3H), 0.88 (t, *J* = 6.4 Hz, 1H), 0.91 (s, 3H), 0.93 (s, 3H), 0.95 (s, 1H), 0.97 (s, 3H), 1.00 (s, 3H), 1.02 (s, 3H), 1.04–1.07 (m, 1H), 1.16 (s, 2H), 1.18–1.21 (m, 2H), 1.25–1.28 (m, 2H), 1.32–1.37 (m, 1H), 1.39–1.43 (m, 1H), 1.45–1.50 (m, 1H), 1.52–1.56 (m, 4H), 1.64–1.73 (m, 4H), 1.78–1.85 (m, 1H), 1.87–1.89 (m, 3H), 1.99 (td, *J* = 3.8, 3.9, 4.1 Hz, 1H), 2.90 (dd, *J* = 4.1, 13.9 Hz, 1H), 4.86 (dd, *J* = 5.2, 12.2 Hz, 1H), 5.00 (d, *J* = 12.2 Hz, 1H), 5.08 (d, *J* = 12.9 Hz, 1H), 5.30 (s, 1H), 7.26–7.33 (m, 4H), 7.49–7.54 (m, 2H), 7.59–7.62 (m, 1H), 7.88 (d, *J* = 7.6 Hz, 1H), 8.00 (d, *J* = 6.9 Hz, 1H), 8.17 (d, *J* = 7.3 Hz, 1H), 8.95 (d, *J* = 10.1 Hz, 1H). ^13^C NMR (125 MHz, CDCl_3_): 15.4 (CH_3_), 16.9 (CH_3_), 23.1 (CH_3_), 23.4 (CH_3_), 23.6 (2CH_3_), 23.7 (CH_3_), 25.9 (CH_3_), 27.6 (CH_2_), 30.7 (2CH_2_), 30.1 (CH_2_), 32.4 (CH_2_), 32.7 (CH_2_), 33.1 (CH_2_), 33.8 (CH_2_), 36.9 (CH_2_), 38.1 (CH_2_), 38.2 (CH), 39.3 (CH), 41.4 (C), 41.7 (2C), 45.8 (C), 46.7 (C), 47.6 (C), 55.4 (CH), 65.1 (CH_2_), 81.7 (CH), 122.5 (CH), 124.5 (C), 125.9 (CH), 126.1 (CH), 127.6 (CH), 127.9 (CH), 128.5 (C), 128.6 (2CH), 129.6 (2CH), 129.8 (C), 131.4 (C), 133.1 (C), 133.9 (C), 134.9 (C), 143.7 (C), 167.3 (C), 177.4 (C). HRMS (ESI) calcd. for C_48_H_59_ClO_4_ [M + H]^+^: m/z = 734.41019; found: 734.41048. (Spectral Data: Supplementary Information Figures S22–S24).

4-Iodobenzyl (4aS,6aS,6bR,8aR,10S,12aR,12bR,14bS)-10-((2,4-dimethoxybenzoyl)oxy)-2,2,6a,6b,9,9,12a-heptamethyl-1,3,4,5,6,6a,6b,7,8,8a,9,10,11,12,12a,12b,13,14b-octadecahydropicene-4a(2H)-carboxylate (NOA-5)

Yield: 45%. M.p. 145–146 °C. ^1^H NMR (600 MHz, CDCl_3_): 0.55 (s, 3H), 0.87–0.89 (m, 1H), 0.90 (s, 3H), 0.92 (s, 3H), 0.95 (s, 6H), 0.97 (s, 3H), 1.02–1.11 (m, 2H), 1.14 (s, 3H), 1.16–1.27 (s, 3H), 1.31–1.47 (m, 3H), 1.51–1.56 (m, 3H), 1.58 (s, 1H), 1.63–1.72 (m, 5H), 1.77–1.87 (m, 2H), 1.98 (td, *J* = 4.0, 4.1, 4.3 Hz, 1H), 2.88 (dd, *J* = 4.3, 13.5 Hz, 1H), 3.85 (s, 3H), 3.88 (s, 3H), 4.69 (dd, *J* = 5.2, 11.2 Hz, 1H), 4.97 (d, *J* = 11.8 Hz, 1H), 5.04 (d, *J* = 13.4 Hz, 1H), 5.28 (s, 1H), 6.47–6.50 (m, 2H), 7.10 (d, *J* = 7.8 Hz, 2H), 7.68 (d, *J* = 9.5 Hz, 2H), 7.86 (d, *J* = 9.5 Hz, 2H). ^13^C NMR (125 MHz, CDCl_3_): 15.5 (2CH_3_), 16.9 (CH_3_), 17.0 (CH_3_), 18.3 (CH_3_), 23.1 (CH_3_), 23.4 (CH_3_), 23.7 (2CH_2_), 25.9 (CH_2_), 27.6 (CH_2_), 28.2 (CH_2_), 30.7 (CH_2_), 32.4 (CH_2_), 32.7 (CH_2_), 33.1 (CH_2_), 33.9 (CH_2_), 36.9 (CH_2_), 38.0 (CH), 38.2 (CH), 39.4 (C), 41.4 (C), 41.7 (C), 45.9 (C), 46.8 (C), 47.6 (C), 55.4 (CH), 55.5 (CH_3_), 55.8 (CH_3_), 65.3 (2C), 80.9 (CH), 93.9 (C), 98.9 (CH), 104.5 (CH), 113.1 (C), 122.5 (CH), 130.1 (CH), 133.7 (CH), 136.1 (CH), 137.5 (CH), 143.7 (CH), 161.4 (C), 164.1 (C), 165.4 (C), 177.4 (C). HRMS (ESI) calcd. for C_46_H_61_IO_6_ [M + H]^+^: m/z = 836.35128; found: 836.35143. (Spectral Data: Supplementary Information Figures S25–S27).

4-Iodobenzyl (4aS,6aS,6bR,8aR,10S,12aR,12bR,14bS)-2,2,6a,6b,9,9,12a-heptamethyl-10-((3,4,5-trimethoxybenzoyl)oxy)-1,3,4,5,6,6a,6b,7,8,8a,9,10,11,12,12a,12b,13,14b-octadecahydropicene-4a(2H)-carboxylate (NOA-6)

Yield: 47%. M.p. 152–153 °C. ^1^H NMR (600 MHz, CDCl_3_): 0.56 (s, 3H), 0.91 (s, 3H), 0.92 (s, 3H), 0.94 (s, 3H), 0.96 (s, 3H), 1.00 (s, 3H), 1.02–1.06 (m, 1H), 1.14 (s, 3H), 1.17–1.26 (m, 3H), 1.31–1.48 (m, 4H), 1.52–1.60 (m, 5H), 1.63–1.77 (m, 6H), 1.85–1.88 (m, 2H), 1.99 (td, *J* = 3.7, 3.9, 4.2 Hz, 1H), 2.88 (dd, *J* = 3.8, 13.9 Hz, 1H), 3.91 (s, 9H), 4.72 (dd, *J* = 6.6, 10.7 Hz, 1H), 4.97 (d, *J* = 12.4 Hz, 1H), 5.05 (d, *J* = 12.7 Hz, 1H), 5.28 (s, 1H), 7.10 (d, *J* = 9.1 Hz, 2H), 7.31 (s, 2H), 7.68 (d, *J* = 8.4 Hz, 2H). ^13^C NMR (125 MHz, CDCl_3_): 15.4 (CH_3_), 16.8 (CH_3_), 17.0 (CH_3_), 18.2 (CH_3_), 23.1 (CH_3_), 23.4 (CH_3_), 23.6 (CH_3_), 25.9 (CH_2_), 27.6 (CH_2_), 28.3 (CH_2_), 30.7 (CH_2_), 32.4 (CH_2_), 32.6 (CH_2_), 33.1 (CH_2_), 33.8 (CH_2_), 36.9 (CH_2_), 38.1 (2CH), 39.3 (CH_2_), 41.4 (C), 41.7 (C), 45.8 (C), 46.7 (C), 47.5 (C), 55.3 (CH), 56.2 (3CH_3_), 60.9 (C), 65.2 (CH_2_), 81.7 (CH), 93.6 (C), 106.8 (2CH), 122.5 (CH), 126.0 (C), 130.1 (2CH), 136.1 (C), 137.5 (2CH), 142.1 (C), 143.7 (C), 152.9 (2C), 165.9 (C), 177.4 (C). HRMS (ESI) calcd. for C_47_H_63_IO_7_ [M + H]^+^: m/z = 866.36185; found: 866.36101. (Spectral Data: Supplementary Information Figures S28–S30).

4-Iodobenzyl (4aS,6aS,6bR,8aR,10S,12aR,12bR,14bS)-10-(benzoyloxy)-2,2,6a,6b,9,9,12a-heptamethyl-1,3,4,5,6,6a,6b,7,8,8a,9,10,11,12,12a,12b,13,14b-octadecahydropicene-4a(2H)-carboxylate (NOA-7)

Yield: 43%. M.p. 146–147 °C. ^1^H NMR (600 MHz, CDCl_3_): 0.56 (s, 3H), 0.87–0.89 (m, 1H), 0.90 (s, 3H), 0.92 (s, 3H), 0.94 (s, 3H), 0.96 (s, 3H), 1.02 (s, 3H), 1.03–1.06 (m, 1H), 1.08–1.12 (m, 1H), 1.14 (s, 3H), 1.18–1.25 (m, 4H), 1.31–1.48 (m, 3H), 1.52–1.59 (m, 4H), 1.62–1.68 (m, 3H), 1.70–1.73 (m, 1H), 1.75–1.77 (m, 1H), 1.85–1.87 (m, 2H), 1.99 (td, *J* = 3.7, 3.8, 4.1 Hz, 1H), 2.88 (dd, *J* = 3.8, 13.7 Hz, 1H), 4.73 (dd, *J* = 5.1, 10.9 Hz, 1H), 4.97 (d, *J* = 13.1 Hz, 1H), 5.04 (d, *J* = 11.8 Hz, 1H), 5.28 (s, 1H), 7.10 (d, *J* = 9.9 Hz, 2H), 7.44 (t, *J* = 8.4 Hz, 2H), 7.55 (t, *J* = 7.7 Hz, 1H), 7.68 (t, *J* = 8.4 Hz, 2H), 8.04 (d, *J* = 7.6 Hz, 2H). ^13^C NMR (125 MHz, CDCl_3_): 15.5 (CH_3_), 16.8 (CH_3_), 17.0 (CH_3_), 18.2 (CH_3_), 23.1 (CH_3_), 23.4 (CH_3_), 23.6 (CH_3_), 23.7 (CH_2_), 25.9 (2CH_2_), 27.6 (CH_2_), 28.2 (CH_2_), 30.7 (2CH_2_), 32.4 (CH_2_), 32.7 (CH_2_), 33.1 (CH_2_), 33.8 (CH_2_), 36.9 (CH), 38.1 (CH), 39.3 (C), 41.4 (C), 41.7 (C), 45.8 (C), 46.8 (C), 47.5 (C), 55.4 (CH), 65.2 (CH_2_), 81.6 (CH), 93.6 (C), 122.5 (CH), 128.3 (2CH), 129.5 (2C), 130.1 (2CH), 132.7 (CH), 136.1 (C), 137.6 (2CH), 143.7 (C), 166.3 (C), 177.4 (C). HRMS (ESI) calcd. for C_44_H_57_IO_4_ [M + H]^+^: m/z = 776.33015; found: 776.32929. (Spectral Data: Supplementary Information Figures S31–S33).

4-Iodobenzyl (4aS,6aS,6bR,8aR,10S,12aR,12bR,14bS)-10-((1-naphthoyl)oxy)-2,2,6a,6b,9,9,12a-heptamethyl-1,3,4,5,6,6a,6b,7,8,8a,9,10,11,12,12a,12b,13,14b-octadecahydropicene-4a(2H)-carboxylate (NOA-8)

Yield: 35%. M.p. 155–156 °C. ^1^H NMR (600 MHz, CDCl_3_): 0.59 (s, 3H), 0.91 (t, *J* = 7.1 Hz, 1H), 0.94 (s, 3H), 0.95 (s, 3H), 0.98 (s, 1H), 1.01 (s, 3H), 1.03 (s, 3H), 1.05 (s, 3H), 1.07–1.09 (m, 1H), 1.18 (s, 3H), 1.21–1.23 (m, 2H), 1.27–1.32 (m, 2H), 1.34–1.41 (m, 1H), 1.43–1.53 (m, 2H), 1.55–1.64 (m, 4H), 1.66–1.76 (m, 3H), 1.81–1.88 (m, 1H), 1.89–1.92 (m, 3H), 2.02 (td, *J* = 3.8 Hz, 1H), 2.92 (dd, *J* = 4.0, 14.2 Hz, 1H), 4.89 (dd, *J* = 8.1, 11.6 Hz, 1H), 5.00 (d, *J* = 12.6 Hz, 1H), 5.07 (d, *J* = 12.4 Hz, 1H), 5.32 (s, 1H), 7.13 (d, *J* = 7.7 Hz, 2H), 7.51–7.57 (m, 2H), 7.63 (t, *J* = 7.7 Hz, 1H), 7.71 (d, *J* = 7.2 Hz, 2H), 7.91 (d, *J* = 9.7 Hz, 1H), 8.04 (d, *J* = 7.3 Hz, 1H), 8.20 (d, *J* = 7.2 Hz, 1H), 8.98 (d, *J* = 7.7 Hz, 1H). ^13^C NMR (125 MHz, CDCl_3_): 15.4 (CH_3_), 16.8 (CH_3_), 17.2 (CH_3_), 18.2 (CH_3_), 22.7 (CH_3_), 23.1 (CH_3_), 23.5 (CH_3_), 23.7 (CH_2_), 25.9 (CH_2_), 27.6 (CH_2_), 28.3 (CH_2_), 30.7 (CH_2_), 31.6 (CH_2_), 32.4 (CH_2_), 32.7 (CH_2_), 33.1 (CH_2_), 33.9 (CH_2_), 37.0 (CH), 38.2 (CH), 39.4 (C), 41.4 (C), 41.7 (C), 45.8 (C), 46.8 (C), 47.5 (C), 55.5 (CH), 65.2 (CH_2_), 81.8 (CH), 93.7 (C), 122.7 (CH), 124.7 (CH), 125.9 (CH), 126.2 (CH), 127.6 (CH), 127.9 (C), 128.4 (CH), 129.9 (CH), 130.2 (2CH), 131.5 (C), 133.1 (CH), 133.9 (C), 135.9 (C), 137.6 (2CH), 143.7 (C), 167.2 (C), 177.4 (C). HRMS (ESI) calcd. for C_48_H_59_IO_4_ [M + H]^+^: m/z = 826.3458; found: 826.34622. (Spectral Data: Supplementary Information Figures S34–S36).

4-Chloro-3-fluorobenzyl (4aS,6aS,6bR,8aR,10S,12aR,12bR,14bS)-10-((2,4-dimethoxybenzoyl)oxy)-2,2,6a,6b,9,9,12a-heptamethyl-1,3,4,5,6,6a,6b,7,8,8a,9,10,11,12,12a,12b,13,14b-octadecahydropicene-4a(2H)-carboxylate (NOA-9)

Yield: 42%. M.p. 139–140 °C. ^1^H NMR (600 MHz, CDCl_3_): 0.57 (s, 3H), 0.87–0.89 (m, 1H), 0.91 (s, 3H), 0.93 (s, 3H), 0.94 (s, 3H), 0.95 (s, 3H), 0.97 (s, 3H), 1.05–1.13 (m, 2H), 1.15 (s, 3H), 1.18–1.25 (m, 3H), 1.32–1.49 (m, 3H), 1.53–1.59 (m, 4H), 1.64–1.73 (m, 5H), 1.78–1.80 (m, 1H), 1.86–1.88 (m, 2H), 2.00 (td, *J* = 3.1, 4.3 Hz, 1H), 2.89 (dd, *J* = 3.4, 14.1 Hz, 1H), 3.85 (s, 3H), 3.88 (s, 3H), 4.69 (dd, *J* = 4.3, 11.2 Hz, 1H), 4.97 (d, *J* = 13.4 Hz, 1H), 5.09 (d, *J* = 13.0 Hz, 1H), 5.30 (s, 1H), 6.47–6.50 (m, 2H), 7.07 (d, *J* = 8.4 Hz, 1H), 7.16 (d, *J* = 9.5 Hz, 1H), 7.37 (t, *J* = 8.4 Hz, 1H), 7.86 (d, *J* = 9.0 Hz, 1H). ^13^C NMR (125 MHz, DMSO-*d_6_*): 15.4 (CH_3_), 16.8 (CH_3_), 17.0 (CH_3_), 18.2 (CH_3_), 23.1 (CH_3_), 23.4 (CH_3_), 23.6 (CH_3_), 25.9 (CH_2_), 27.6 (CH_2_), 28.2 (CH_2_), 30.7 (CH_2_), 32.4 (CH_2_), 32.7 (CH_2_), 33.1 (CH_2_), 33.8 (CH_2_), 36.9 (CH_2_), 38.0 (CH_2_), 38.2 (CH_2_), 39.3 (CH), 41.4 (C), 41.7 (C), 45.8 (C), 46.8 (C), 47.4 (C), 55.4 (CH), 55.5 (CH_3_), 55.8 (CH_3_), 64.5 (C), 80.9 (CH), 98.9 (CH), 104.4 (2CH), 113.1 (C), 116.3 (d, *J* = 21.1 Hz, CH), 122.6 (CH), 124.3 (CH), 130.6 (2CH), 133.7 (C), 137.3 (C), 143.6 (C), 157.2 (C), 161.4 (C), 164.1 (C), 165.4 (C), 177.3 (C). HRMS (ESI) calcd. for C_46_H_60_ClFO_6_ [M + H]^+^: m/z = 762.40625; found: 762.40533. (Spectral Data: Supplementary Information Figures S37–S39).

4-Chloro-3-fluorobenzyl (4aS,6aS,6bR,8aR,10S,12aR,12bR,14bS)-2,2,6a,6b,9,9,12a-heptamethyl-10-((3,4,5-trimethoxybenzoyl)oxy)-1,3,4,5,6,6a,6b,7,8,8a,9,10,11,12,12a,12b,13,14b-octadecahydropicene-4a(2H)-carboxylate (NOA-10)

Yield: 40%. M.p. 157–158 °C. ^1^H NMR (600 MHz, CDCl_3_): 0.57 (s, 3H), 0.89 (s, 1H), 0.92 (s, 3H), 0.93 (s, 3H), 0.94 (s, 3H), 0.96 (s, 3H), 1.00 (s, 3H), 1.03–1.13 (m, 2H), 1.15 (s, 3H), 1.18–1.26 (m, 3H), 1.32–1.49 (m, 3H), 1.53–1.61 (m, 4H), 1.64–1.77 (m, 6H), 1.87–1.89 (m, 2H), 2.00 (td, *J* = 3.7, 4.1, 4.4 Hz, 1H), 2.89 (dd, *J* = 3.9, 13.7 Hz, 1H), 3.91 (s, 9H), 4.72 (dd, *J* = 6.0, 10.5 Hz, 1H), 4.97 (d, *J* = 12.6 Hz, 1H), 5.09 (d, *J* = 12.6 Hz, 1H), 5.31 (s, 1H), 7.08 (d, *J* = 9.9 Hz, 1H), 7.16 (d, *J* = 10.5 Hz, 1H), 7.31 (s, 2H), 7.37 (d, *J* = 9.3 Hz, 1H). ^13^C NMR (125 MHz, DMSO-*d_6_*): 15.3 (CH_3_), 16.8 (CH_3_), 17.0 (CH_3_), 18.2 (CH_3_), 23.1 (CH_3_), 23.4 (CH_3_), 23.4 (CH_3_), 23.6 (2CH_2_), 25.9 (CH_2_), 27.6 (CH_2_), 28.3 (CH_2_), 30.7 (CH_2_), 32.4 (CH_2_), 32.7 (CH_2_), 33.1 (CH_2_), 33.8 (CH_2_), 36.9 (CH), 38.1 (CH), 39.3 (C), 41.4 (C), 41.7 (C), 45.8 (C), 46.8 (C), 47.5 (C), 55.3 (CH), 56.2 (3CH_3_), 60.9 (C), 64.5 (CH_2_), 81.6 (CH), 106.8 (2CH), 116.3 (d, *J* = 21.6 Hz, CH), 122.6 (C), 124.3 (CH), 126.0 (C), 130.6 (2CH), 137.3 (C), 142.1 (C), 143.6 (C), 152.9 (2C), 165.9 (C), 177.3 (C). MS (FAB) m/z = 816.0 (M + Na)^+^ (C_47_H_62_ClFO_7_) (Spectral Data: Supplementary Information Figures S40–S42).

4-Chloro-3-fluorobenzyl (4aS,6aS,6bR,8aR,10S,12aR,12bR,14bS)-10-(benzoyloxy)-2,2,6a,6b,9,9,12a-heptamethyl-1,3,4,5,6,6a,6b,7,8,8a,9,10,11,12,12a,12b,13,14b-octadecahydropicene-4a(2H)-carboxylate (NOA-11)

Yield: 42%. M.p. 143–144 °C. ^1^H NMR (600 MHz, CDCl_3_): 0.57 (s, 3H), 0.89 (s, 1H), 0.91 (s, 3H), 0.93 (s, 3H), 0.94 (s, 3H), 0.96 (s, 3H), 1.02 (s, 3H), 1.03–1.12 (m, 2H), 1.15 (s, 3H), 1.18–1.26 (m, 3H), 1.32–1.49 (m, 3H), 1.53–1.60 (m, 4H), 1.64–1.69 (m, 3H), 1.71–1.79 (m, 3H), 1.86–1.88 (m, 2H), 2.00 (td, *J* = 3.7, 3.8, 4.4 Hz, 1H), 2.89 (dd, *J* = 4.0, 13.3 Hz, 1H), 4.73 (dd, *J* = 4.2, 15.5 Hz, 1H), 4.97 (d, *J* = 13.2 Hz, 1H), 5.09 (d, *J* = 13.2 Hz, 1H), 5.30 (s, 1H), 7.07 (d, *J* = 7.1 Hz, 1H), 7.16 (d, *J* = 9.8 Hz, 1H), 7.37 (t, *J* = 8.4 Hz, 1H), 7.44 (t, *J* = 8.7 Hz, 2H), 7.55 (t, *J* = 7.3 Hz, 1H), 8.04 (d, *J* = 7.1 Hz, 2H). ^13^C NMR (125 MHz, CDCl_3_): 15.4 (CH_3_), 16.9 (CH_3_), 17.0 (CH_3_), 18.3 (CH_3_), 23.1 (CH_3_), 23.4 (CH_3_), 23.6 (CH_3_), 23.7 (CH_2_), 25.9 (CH_2_), 27.6 (CH_2_), 28.3 (CH_2_), 30.8 (2CH_2_), 32.4 (CH_2_), 32.7 (CH_2_), 33.2 (CH_2_), 33.7 (CH_2_), 36.9 (CH), 38.1 (CH), 39.3 (C), 41.5 (C), 41.8 (C), 45.9 (C), 46.9 (C), 47.6 (C), 55.5 (CH), 64.6 (CH_2_), 81.7 (CH), 116.3 (d, *J* = 28.6 Hz, CH), 122.6 (C), 124.4 (CH), 128.4 (3CH), 129.6 (2CH), 130.6 (CH), 131.1 (C), 132.8 (CH), 137.3 (C), 143.8 (C), 157.6 (C), 166.2 (C), 177.4(C). HRMS (ESI) calcd. for C_44_H_56_ClFO_4_ [M + H]^+^: m/z = 702.38512; found: 702.38395. (Spectral Data: Supplementary Information Figures S43–S45).

4-Chloro-3-fluorobenzyl (4aS,6aS,6bR,8aR,10S,12aR,12bR,14bS)-10-((1-naphthoyl)oxy)-2,2,6a,6b,9,9,12a-heptamethyl-1,3,4,5,6,6a,6b,7,8,8a,9,10,11,12,12a,12b,13,14b-octadecahydropicene-4a(2H)-carboxylate (NOA-12)

Yield: 38%. M.p. 159–160 °C. ^1^H NMR (600 MHz, CDCl_3_): 0.60 (s, 3H), 0.94 (s, 3H), 0.96 (s, 3H), 0.98 (s, 1H), 0.99 (s, 3H), 1.03 (s, 3H), 1.05 (s, 3H), 1.07–1.11 (m, 1H), 1.19 (s, 3H), 1.20–1.30 (m, 2H), 1.35–1.53 (m, 4H), 1.55–1.64 (m, 5H), 1.67–1.77 (m, 4H), 1.80–1.88 (m, 1H), 1.89–1.93 (m, 3H), 2.03 (td, *J* = 4.1, 4.3 Hz, 1H), 2.92 (dd, *J* = 4.0, 13.7 Hz, 1H), 4.89 (dd, *J* = 4.3, 10.9 Hz, 1H), 4.99 (d, *J* = 12.7 Hz, 1H), 5.12 (d, *J* = 11.9 Hz, 1H), 5.34 (s, 1H), 7.10 (d, *J* = 8.1 Hz, 1H), 7.19 (d, *J* = 8.8 Hz, 1H), 7.39 (t, *J* = 8.8 Hz, 1H), 7.51–7.57 (m, 2H), 7.63 (t, *J* = 7.7 Hz, 1H), 7.91 (d, *J* = 8.1 Hz, 1H), 8.04 (d, *J* = 8.5 Hz, 1H), 8.20 (d, *J* = 7.4 Hz, 1H), 8.97 (d, *J* = 8.1 Hz, 1H). ^13^C NMR (125 MHz, CDCl_3_): 15.4 (CH_3_), 16.9 (CH_3_), 17.2 (CH_3_), 18.2 (CH_3_), 23.1 (CH_3_), 23.6 (CH_3_), 23.7 (CH_3_), 25.9 (CH_2_), 27.6 (CH_2_), 28.3 (CH_2_), 30.8 (2CH_2_), 32.5 (CH_2_), 32.7 (CH_2_), 33.1 (CH_2_), 33.8 (CH_2_), 37.0 (CH_2_), 38.1 (CH), 38.3 (CH), 39.3 (C), 41.4 (C), 41.7 (C), 45.8 (C), 46.9 (C), 47.6 (C), 55.5 (CH), 64.5 (CH_2_), 81.8 (CH), 116.3 (d, *J* = 26.7 Hz, CH), 122.6 (C), 124.4 (CH), 124.6 (CH), 125.9 (C), 126.2 (CH), 127.6 (CH), 128.0 (CH), 128.5 (CH), 129.8 (CH), 130.7 (2C), 131.5 (CH), 133.2 (CH), 133.9 (CH), 137.4 (C), 143.6 (C), 158.9 (C), 167.3 (C), 177.4 (C). HRMS (ESI) calcd. for C_48_H_58_ClFO_4_ [M + H]^+^: m/z = 752.40077; found: 752.39755. (Spectral Data: Supplementary Information Figures S46–S48).

3,4-Dichlorobenzyl (4aS,6aS,6bR,8aR,10S,12aR,12bR,14bS)-10-((2,4-dimethoxybenzoyl)oxy)-2,2,6a,6b,9,9,12a-heptamethyl-1,3,4,5,6,6a,6b,7,8,8a,9,10,11,12,12a,12b,13,14b-octadecahydropicene-4a(2H)-carboxylate (NOA-13)

Yield: 40%. M.p. 142–143 °C. ^1^H NMR (600 MHz, CDCl_3_): 0.53 (d, *J* = 10.3 Hz, 3H), 0.87–0.89 (m, 2H), 0.90 (s, 1H), 0.91 (s, 2H), 0.92 (s, 3H), 0.93 (s, 2H), 0.95 (s, 2H), 0.97 (s, 2H), 0.98 (s, 1H), 1.02–1.09 (m, 2H), 1.12 (s, 1H), 1.14 (s, 3H), 1.18–1.26 (m, 3H), 1.29–1.46 (m, 3H), 1.49–1.60 (m, 4H), 1.62–1.65 (m, 3H), 1.68–1.73 (m, 2H), 1.77–1.81 (m, 1H), 1.84–1.88 (m, 2H), 1.96–2.02 (m, 1H), 2.88 (d, *J* = 14.6 Hz, 1H), 3.84 (s, 3H), 3.88 (s, 3H), 4.69 (dd, *J* = 4.9, 11.9 Hz, 1H), 4.95 (d, *J* = 12.6 Hz, 1H), 5.10 (d, *J* = 12.9 Hz, 1H), 5.30 (s, 1H), 6.47–6.50 (m, 2H), 7.17–7.19 (m, 1H), 7.41 (d, *J* = 7.9 Hz, 1H), 7.45–7.46 (m, 1H), 7.86 (d, *J* = 8.3 Hz, 1H). ^13^C NMR (125 MHz, CDCl_3_): 15.3 (CH_3_), 15.4 (CH_3_), 16.8 (CH_3_), 17.0 (CH_3_), 18.2 (CH_3_), 23.1 (CH_3_), 23.4 (CH_3_), 23.6 (CH_2_), 25.9 (CH_2_), 27.5 (CH_2_), 28.2 (CH_2_), 30.7 (CH_2_), 32.4 (CH_2_), 32.7 (CH_2_), 33.1 (CH_2_), 33.8 (CH_2_), 36.9 (CH_2_), 38.0 (2CH), 38.8 (CH), 39.3 (CH), 41.4 (C), 41.7 (C), 45.8 (C), 46.8 (C), 47.5 (C), 55.4 (CH), 55.5 (CH_3_), 55.8 (CH_3_), 64.4 (C), 80.9 (CH), 98.9 (CH), 104.4 (CH), 113.1 (C), 122.6 (CH), 127.4 (CH), 130.2 (CH), 130.4 (CH), 132.6 (CH), 133.7 (C), 136.6 (C), 143.6 (C), 161.4 (C), 164.1 (C), 165.4 (C), 177.3 (C). HRMS (ESI) calcd. for C_46_H_60_Cl_2_O_6_ [M + H]^+^: m/z = 778.3767; found: 778.37646. (Spectral Data: Supplementary Information Figures S49–S51).

3,4-Dichlorobenzyl (4aS,6aS,6bR,8aR,10S,12aR,12bR,14bS)-2,2,6a,6b,9,9,12a-heptamethyl-10-((3,4,5-trimethoxybenzoyl)oxy)-1,3,4,5,6,6a,6b,7,8,8a,9,10,11,12,12a,12b,13,14b-octadecahydropicene-4a(2H)-carboxylate (NOA-14)

Yield: 42%. M.p. 159–160 °C. ^1^H NMR (600 MHz, CDCl_3_): 0.54 (s, 3H), 0.88–0.90 (m, 1H), 0.91 (s, 3H), 0.93 (s, 3H), 0.94 (s, 3H), 0.96 (s, 3H), 1.01 (s, 3H), 1.04–1.13 (m, 2H), 1.15 (s, 3H), 1.18–1.26 (m, 3H), 1.33–1.48 (m, 3H), 1.52–1.59 (m, 3H), 1.62–1.70 (m, 4H), 1.71–1.77 (m, 3H), 1.88 (dd, *J* = 3.2, 8.7 Hz, 2H), 2.00 (td, *J* = 3.7, 4.1, 4.4 Hz, 1H), 2.89 (dd, *J* = 4.3, 13.6 Hz, 1H), 3.91 (s, 9H), 4.72 (dd, *J* = 6.1, 9.5 Hz, 1H), 4.95 (d, *J* = 13.4 Hz, 1H), 5.09 (d, *J* = 12.8 Hz, 1H), 5.30 (s, 1H), 7.18–7.19 (m, 1H), 7.31 (s, 2H), 7.42 (d, *J* = 8.6 Hz, 1H), 7.47 (s, 1H). ^13^C NMR (125 MHz, CDCl_3_): 15.3 (CH_3_), 16.8 (CH_3_), 17.0 (CH_3_), 18.2 (CH_3_), 23.1 (CH_3_), 23.4 (CH_3_), 23.6 (CH_3_), 25.9 (CH_2_), 27.5 (CH_2_), 28.3 (CH_2_), 30.7 (CH_2_), 32.4 (CH_2_), 32.6 (CH_2_), 33.1 (CH_2_), 33.8 (CH_2_), 36.9 (C), 38.1 (2CH_2_), 39.3 (CH), 41.4 (CH), 41.7 (C), 45.8 (C), 46.8 (C), 47.6 (C), 55.3 (CH), 56.2 (3CH_3_), 60.9 (C), 64.4 (CH_2_), 81.6 (CH), 106.8 (2CH), 122.6 (CH), 126.0 (C), 127.5 (CH), 130.2 (CH), 130.4 (CH), 132.1 (C), 132.6 (C), 136.6 (C), 142.1 (C), 143.7 (C), 152.9 (2C), 165.9 (C), 177.3 (C). HRMS (ESI) calcd. for C_47_H_62_Cl_2_O_7_ [M + H]^+^: m/z = 808.38726; found: 808.38739. (Spectral Data: Supplementary Information Figures S52–S54).

3,4-Dichlorobenzyl (4aS,6aS,6bR,8aR,10S,12aR,12bR,14bS)-10-(benzoyloxy)-2,2,6a,6b,9,9,12a-heptamethyl-1,3,4,5,6,6a,6b,7,8,8a,9,10,11,12,12a,12b,13,14b-octadecahydropicene-4a(2H)-carboxylate (NOA-15)

Yield: 35%. M.p. 141–142 °C. ^1^H NMR (600 MHz, CDCl_3_): 0.54 (s, 3H), 0.89 (s, 1H), 0.91 (s, 3H), 0.93 (s, 3H), 0.94 (s, 3H), 0.95 (s, 3H), 1.01 (s, 3H), 1.04–1.12 (m, 2H), 1.14 (s, 3H), 1.18–1.26 (m, 3H), 1.32–1.48 (m, 3H), 1.52–1.59 (m, 4H), 1.63–1.79 (m, 6H), 1.88 (dd, *J* = 3.7, 8.6 Hz, 2H), 2.00 (td, *J* = 3.6, 4.3, 4.6 Hz, 1H), 2.89 (dd, *J* = 4.6, 14.2 Hz, 1H), 4.73 (dd, *J* = 4.5, 10.8 Hz, 1H), 4.95 (d, *J* = 12.9 Hz, 1H), 5.08 (d, *J* = 12.3 Hz, 1H), 5.30 (s, 1H), 7.18 (d, *J* = 8.3 Hz, 1H), 7.41–7.47 (m, 4H), 7.55 (t, *J* = 7.9 Hz, 1H), 8.04 (d, *J* = 7. Hz, 2H). ^13^C NMR (125 MHz, CDCl_3_): 15.3 (CH_3_), 16.8 (CH_3_), 16.9 (CH_3_), 18.2 (CH_3_), 23.1 (CH_3_), 23.4 (CH_3_), 23.5 (CH_3_), 23.6 (CH_2_), 25.9 (CH_2_), 27.5 (CH_2_), 28.2 (2CH_2_), 30.7 (CH_2_), 32.4 (CH_2_), 32.6 (CH_2_), 33.1 (CH_2_), 33.8 (CH_2_), 36.9 (CH), 38.1 (CH), 39.3 (CH), 41.4 (C), 41.7 (C), 45.8 (C), 46.8 (C), 47.5 (C), 55.4 (CH), 64.4 (CH_2_), 81.5 (CH), 122.6 (CH), 127.5 (3CH), 128.3 (CH), 129.5 (CH), 130.2 (CH), 130.4 (CH), 130.9, (C), 132.1 (C), 132.7 (C), 134.1 (C), 136.6 (C), 143.6 (C), 166.3 (C), 177.3 (C). HRMS (ESI) calcd. for C_44_H_56_Cl_2_O_4_ [M + H]^+^: m/z = 718.35557; found: 718.35412. (Spectral Data: Supplementary Information Figures S55–S57).

3,4-Dichlorobenzyl (4aS,6aS,6bR,8aR,10S,12aR,12bR,14bS)-10-((1-naphthoyl)oxy)-2,2,6a,6b,9,9,12a-heptamethyl-1,3,4,5,6,6a,6b,7,8,8a,9,10,11,12,12a,12b,13,14b-octadecahydropicene-4a(2H)-carboxylate (NOA-16)

Yield: 30%. M.p. 150–151 °C. ^1^H NMR (600 MHz, CDCl_3_): 0.55 (s, 3H), 0.92 (s, 3H), 0.94 (s, 3H), 0.95 (s, 1H), 0.97 (s, 3H), 1.01 (s, 3H), 1.02 (s, 3H), 1.04–1.14 (m, 2H), 1.16 (s, 3H), 1.19–1.27 (m, 4H), 1.33–1.50 (m, 3H), 1.53–1.61 (m, 4H), 1.64–1.71 (m, 3H), 1.74–1.85 (m, 1H), 1.87–1.91 (m, 3H), 2.00 (td, *J* = 3.8, 4.3, 4.6 Hz, 1H), 2.89 (dd, *J* = 3.8, 13.6 Hz, 1H), 4.86 (dd, *J* = 4.7, 11.7 Hz, 1H), 4.95 (d, *J* = 12.7 Hz, 1H), 5.09 (d, *J* = 12.2 Hz, 1H), 5.31 (s, 1H), 7.18 (d, *J* = 7.6 Hz, 1H), 7.42 (d, *J* = 9.3 Hz, 1H), 7.47–7.54 (m, 3H), 7.61 (t, *J* = 7.6 Hz, 1H), 7.88 (d, *J* = 8.3 Hz, 1H), 8.01 (d, *J* = 7.9 Hz, 1H), 8.17 (d, *J* = 8.2 Hz, 1H), 8.95 (d, *J* = 8.8 Hz, 1H). ^13^C NMR (125 MHz, CDCl_3_): 15.4 (CH_3_), 16.8 (CH_3_), 17.2 (CH_3_), 18.2 (CH_3_), 23.1 (CH_3_), 23.5 (CH_3_), 23.8 (CH_3_), 25.9 (CH_2_), 27.6 (CH_2_), 28.4 (CH_2_), 30.7 (CH_2_), 32.4 (CH_2_), 32.7 (CH_2_), 33.1 (CH_2_), 33.9 (CH_2_), 37.1 (CH_2_), 38.1 (CH_2_), 38.3 (CH), 39.4 (CH), 41.5 (C), 41.8 (C), 42.5 (C), 45.8 (C), 46.9 (C), 47.6 (C), 55.5 (CH), 64.5 (CH_2_), 81.7 (CH), 122.6 (CH), 124.6 (CH), 125.9 (C), 126.2 (CH), 127.5 (2CH), 127.7 (CH), 128.5 (CH), 129.8 (CH), 130.2 (CH), 130.4 (CH), 131.3 (CH), 131.5 (C), 132.6 (C), 133.1 (C), 133.9 (C), 136.7 (C), 143.6 (C), 167.5 (C), 177.1 (C). MS (FAB) m/z = 791.4 (M + Na)^+^ (C_48_H_58_Cl_2_O_4_) (Spectral Data: Supplementary Information Figures S58–S60).

### 2.2. Biological Activity

#### 2.2.1. Cell Lines and Culture Conditions

K562 (ATCC^®^ CCL-243™, Manassas, VA, USA), HL-60 (ATCC^®^ CCL-240™, Manassas, VA, USA), MT-2 (ATCC^®^ CRL-1582™, Manassas, VA, USA), Jurkat (ATCC^®^ TIB-152™, Manassas, VA, USA) cells and human peripheral blood mononuclear cells (PBMCs) (Precision Bioservices, Frederick, MD, USA) were cultured in RPMI 1640 medium. All culture media were supplemented with 5% fetal bovine serum (FBS), 89 μg/mL streptomycin, and 89 μg/mL fungizone, and maintained at 37 °C in a humidified atmosphere containing 95% air and 5% CO_2_. Actively growing cancer cells were seeded into 24-well microtiter tissue culture plates at a density of 4 × 10^4^ cells/mL for K562, HL-60, MT-2, and Jurkat cell lines, while PBMCs were seeded into 96-well plates at a density of 1 × 10^6^ cells/mL. After the addition of the test compounds, cells were incubated for 48 h under standard culture conditions. Appropriate cell numbers and incubation times for cytotoxicity assays were determined prior to the experiments. Stock solutions of the synthesized compounds and imatinib (0.1, 0.3, 1, 3, and 10 mM) were prepared in DMSO and subsequently diluted with fresh culture medium. The final concentration of DMSO in the culture medium did not exceed 1% [24,25,26].

#### 2.2.2. Cell Viability Assay

The cellular reduction capacity of 3-(4,5-dimethylthiazol-2-yl)-2,5-diphenyltetrazolium bromide (MTT) was determined with minor modifications according to previously described methods [24,25,26,27]. Cells were incubated with various concentrations of the tested compounds for 48 h. After the incubation period, MTT solution was added to the cells, and the plates were further incubated at 37 °C for 4 h. Following removal of the MTT solution, the resulting formazan crystals were dissolved in 100 µL of DMSO, and the absorbance was measured at 570 nm using an Infinite M1000 multimode microplate reader (Tecan, Grödig, Austria). Each concentration was tested in triplicate. IC_50_ values were defined as the concentrations of the compounds that reduced the absorbance by 50%. The Abl-1 inhibitor, the anticancer drug imatinib, was used as a positive control.

#### 2.2.3. Apoptosis and Necrosis Analysis

Selected compounds were applied at their IC_50_ concentrations to K562 cells seeded in 24-well culture plates at a density of 4 × 10^4^ cells per well. After 12 h of incubation, the culture medium was removed and the cells were washed twice with phosphate-buffered saline (PBS). Subsequently, 50 µL of binding buffer, 4 µL of FITC-Annexin V solution, 4 µL of ethidium homodimer III solution, and 4 µL of Hoechst 33342 solution were sequentially added to the cells. The cells were incubated for 20 min at r.t. in the dark. Following incubation, the cells were washed with binding buffer and analyzed using fluorescence microscopy [24,25,26]. Annexin V-based staining was used to detect apoptotic cells according to previously described methods [28,29]. Cells stained green with Annexin V but not red with ethidium homodimer III were considered apoptotic. Cells stained only red with ethidium homodimer III were classified as necrotic. Cells exhibiting both green and red fluorescence were evaluated as late apoptotic or secondary necrotic cells.

#### 2.2.4. ABL TK Inhibition Assay

The inhibitory effects of MT-A, MT-C, NOA-3, and NOA-8 on ABL TK activity were investigated and compared with the reference inhibitor imatinib using the Promega ABL TK kinase assay system (Promega V1901). Kinase reactions were carried out in a reaction medium consisting of 40 mM Tris buffer (pH 7.5), 20 mM MgCl_2_, and 0.1 mg/mL BSA, supplemented with 10 mM ATP and 2 mM DTT. ABL TK enzyme stock solution was prepared in 2.5× kinase buffer and purified water to obtain a final concentration of 4 ng/µL. The Abltide peptide substrate (EAIYAAPFAKKK) was prepared at a final concentration of 0.2 µg/µL in the presence of 25 µM ATP using kinase buffer and ATP stock solution. For kinase reactions, 4 µL of enzyme solution and 4 µL of ATP/substrate mixture were combined with 2 µL of compound solution or 5% DMSO as the control in 384-well plates. The reaction mixtures were incubated for 2 h at r.t. Following incubation, kinase activity was quantified using the ADP-Glo™ Kinase Assay kit (Promega Corporation, Madison, WI, USA) according to the manufacturer’s protocol. Briefly, ADP-Glo reagent (5 µL) was added to terminate the kinase reaction and eliminate residual ATP, followed by incubation at r.t. for 40 min. Subsequently, 10 µL of Kinase Detection Reagent was introduced into each well and incubated for an additional 30 min to convert generated ADP into ATP and produce the luminescent signal. Dose-dependent inhibition studies were performed for MT-A, MT-C, NOA-3, and NOA-8 and imatinib at concentrations of 100, 30, 10, 3, 1, and 0.1 µM. Luminescence measurements were recorded using an Infinite M1000 multimode microplate reader (Tecan, Grödig, Austria), and the percentage inhibition of ABL TK activity was calculated accordingly [24,25,26].

### 2.3. In Silico Evaluation

#### 2.3.1. Protein and Ligand Preparation

The crystal structures of ABL kinase (PDB ID: 1IEP) [30] and LCK kinase (PDB ID: 3LCK) [31] were obtained from the Protein Data Bank (PDB). Protein structures were prepared using the Protein Preparation Wizard implemented in Maestro (Schrödinger Release 2025-1) [32]. Missing hydrogen atoms were added, bond orders were assigned, and incomplete side chains were reconstructed when necessary. Water molecules located outside the binding region were removed, whereas structurally important water molecules involved in ligand recognition were retained. Protonation states of ionizable residues were assigned at physiological pH (7.4 ± 2.0), followed by optimization of the hydrogen-bonding network and restrained energy minimization using the OPLS4 force field. The chemical structures of the synthesized compounds were prepared with LigPrep (Schrödinger Release 2025-1) [32]. Ionization and tautomeric states were generated using Epik at pH 7.4 ± 2.0, and the resulting structures were energy-minimized with the OPLS4 force field before docking calculations [33].

#### 2.3.2. Receptor Grid Generation and Molecular Docking

Docking grids were generated by centering the receptor grid on the co-crystallized ligand of each kinase structure. The docking region was defined to fully encompass the ATP-binding pocket. Molecular docking was carried out using Glide in Extra Precision (XP) mode with a rigid receptor and fully flexible ligands. The highest-ranked binding poses were selected according to the GlideScore and subsequently analyzed for key protein–ligand interactions [33].

#### 2.3.3. Prime MM-GBSA Binding Energy Calculations

The best docking poses were further evaluated using Prime MM-GBSA (Schrödinger Release 2025-1) to estimate the relative binding free energies. Calculations were performed with the OPLS4 force field and the VSGB 2.0 implicit solvent model. Binding free energies (ΔG_bind_) were calculated from the energies of the protein–ligand complex, the isolated protein, and the free ligand according to the standard MM-GBSA protocol.

$$\Delta G_{\mathrm{bind}}=E_{\mathrm{complex}}-\left(E_{\mathrm{protein}}+E_{\mathrm{ligand}}\right)$$

#### 2.3.4. Molecular Dynamics (MD) Simulations

The stability of the selected protein–ligand complexes was investigated by 100 ns MD simulations using Desmond (Schrödinger Release 2025-1). Each complex was solvated in an orthorhombic TIP3P water box and neutralized with appropriate counterions. Simulations were carried out under NPT conditions (300 K, 1 atm) using the OPLS4 force field. Trajectories were analyzed in terms of backbone RMSD, residue RMSF, protein–ligand contacts, and interaction persistence to evaluate the dynamic stability of the predicted binding modes [33,34].

#### 2.3.5. In Silico Pharmacokinetic Prediction

The pharmacokinetic characteristics and drug-likeness profiles of the two most active ABL-targeting compounds, MT-A and MT-C, were evaluated using the SwissADME web server [35]. Predicted parameters included physicochemical properties, lipophilicity, aqueous solubility, gastrointestinal absorption, P-glycoprotein substrate status, cytochrome P450 inhibition, skin permeability, oral bioavailability, and compliance with commonly used drug-likeness filters (Lipinski, Ghose, Veber, Egan, and Muegge). These predictions were performed to obtain a preliminary assessment of the pharmacokinetic behavior and developability of the selected lead compounds.

## 3. Results

### 3.1. Chemistry

**Scheme 1:**
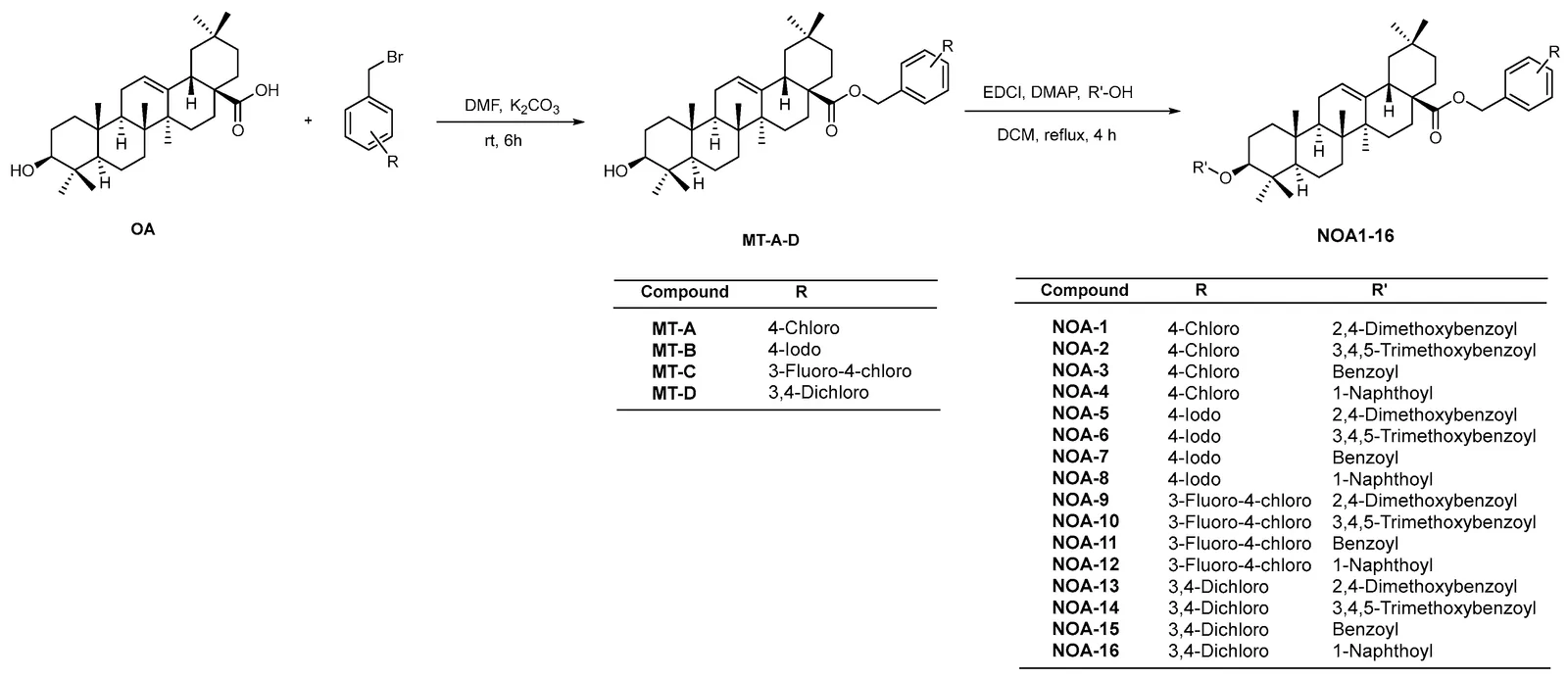
The synthetic route of MT-A-D and NOA1-16.

OA was selected as the starting scaffold for semi-synthetic modification. As shown in Scheme 1, esterification of the C-28 carboxylic acid group with four halogenated benzyl bromides afforded the MT-A-D derivatives. Further modification at the C-3 position with various aromatic acyl moieties yielded sixteen final derivatives (NOA-1-16). All synthesized compounds were successfully obtained and their structures were confirmed by ^1^H NMR, ^13^C NMR, LC-MS/HRMS analyses. The synthesized derivatives were subsequently evaluated for their antiproliferative activities against leukemia cell lines, and their biological properties were further investigated through apoptosis, kinase inhibition, and in silico studies.

### 3.2. Antiproliferative Activity

The antiproliferative activities of the synthesized compounds were evaluated against K562, HL-60, Jurkat, and MT-2 leukemia cell lines using the MTT assay.

**Figure 1:**
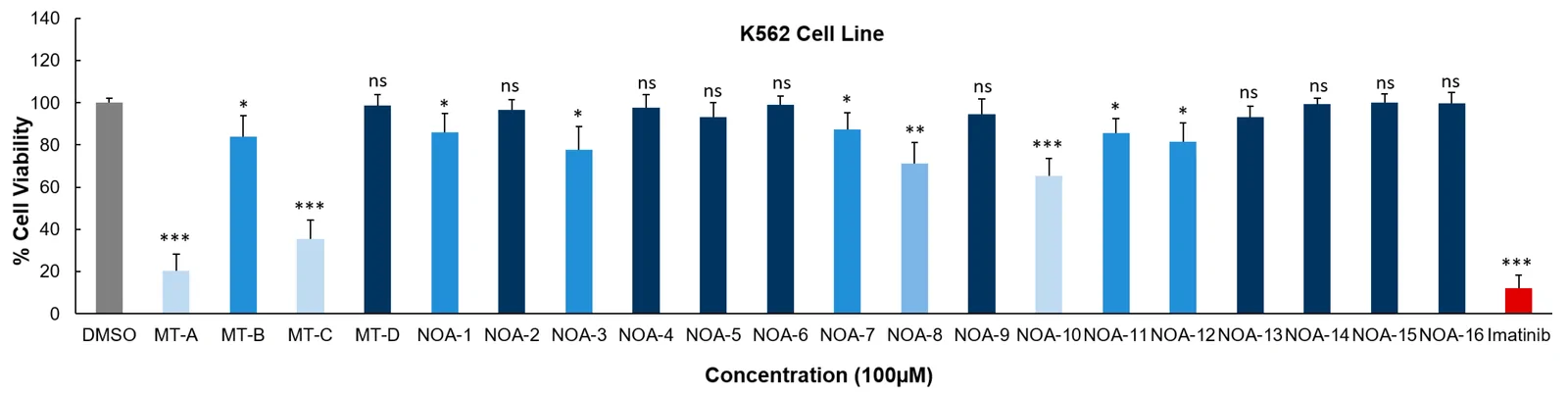
Cytotoxic activity of MT-A-D and NOA1-16 derivatives against K562 CML cells at 100 µM. Cell viability was determined using the MTT assay after 48 h of treatment. Data are presented as mean ± SD of three independent experiments (*n* = 3). Statistical significance was determined using one-way ANOVA followed by Dunnett’s multiple comparisons test. ns, not significant; * *p* < 0.05; ** *p* < 0.01; *** *p* < 0.001 versus the DMSO control group.

The synthesized compounds were initially screened against K562 cells at 100 µM concentration (Figure 1 and Table 1). Among the MT derivatives, MT-A and MT-C showed the most pronounced antiproliferative effects, reducing cell viability to 20.33% and 35.55%, respectively. In contrast, MT-B and MT-D displayed limited activity, with cell viability values of 83.87% and 98.72%, respectively.

Evaluation of the NOA derivatives revealed generally weaker activity against K562 cells. Most compounds-maintained cell viability above 80%, although NOA-10 (65.5%), NOA-8 (71.09%), and NOA-3 (77.81%) demonstrated relatively stronger inhibition than the remaining derivatives. Nevertheless, imatinib remained the most potent compound under identical conditions, reducing cell viability to 12.26%. IC_50_ analyses further supported these findings, with MT-A and MT-C exhibiting IC_50_ values of 80.03 ± 10.26 µM and 72.60 ± 8.73 µM, respectively, whereas most NOA derivatives showed IC_50_ values above 100 µM (Table 1).

**Figure 2:**
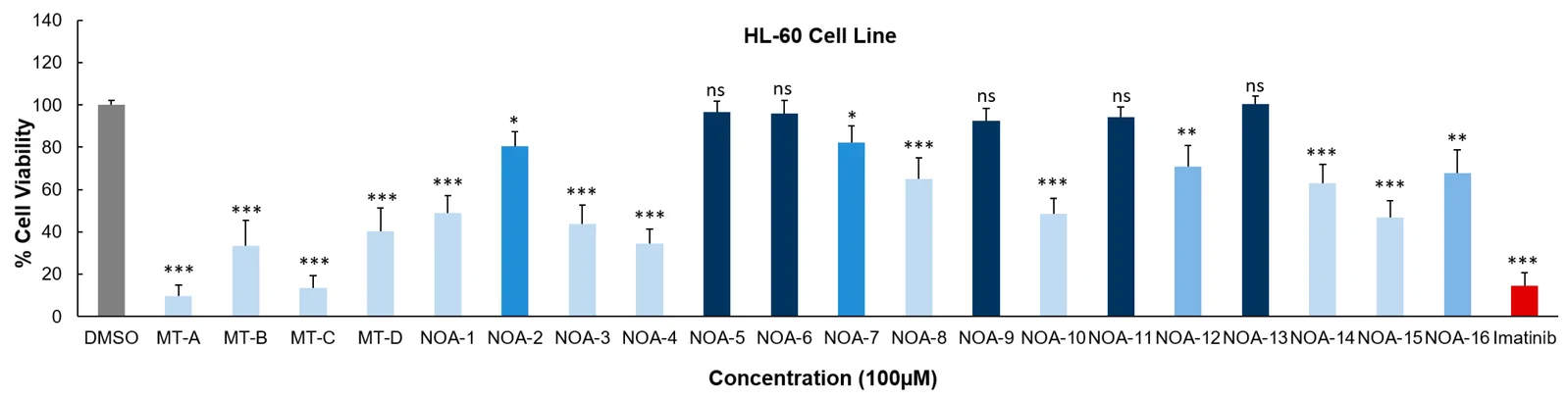
Cytotoxic activity of MT-A-D and NOA1-16 derivatives against HL-60 cells at 100 µM. Cell viability was determined using the MTT assay after 48 h of treatment. Data are presented as mean ± SD of three independent experiments (*n* = 3). Statistical significance was determined using one-way ANOVA followed by Dunnett’s multiple comparisons test. ns, not significant; * *p* < 0.05; ** *p* < 0.01; *** *p* < 0.001 versus the DMSO control group.

To further investigate their anti-leukemic potential, the compounds were evaluated in HL-60, Jurkat, and MT-2 leukemia cell lines. In HL-60 cells, the MT derivatives demonstrated substantially stronger activity than the NOA series (Figure 2). MT-A exhibited the highest potency with an IC_50_ value of 4.17 ± 0.65 µM, surpassing imatinib (13.34 ± 1.92 µM). MT-B also displayed notable activity (9.74 ± 1.08 µM), while MT-C showed moderate inhibition comparable to imatinib (16.36 ± 2.41 µM). By contrast, most NOA derivatives showed weak or negligible activity, with only NOA-3 demonstrating moderate cytotoxicity (45.10 ± 4.16 µM).

**Figure 3:**
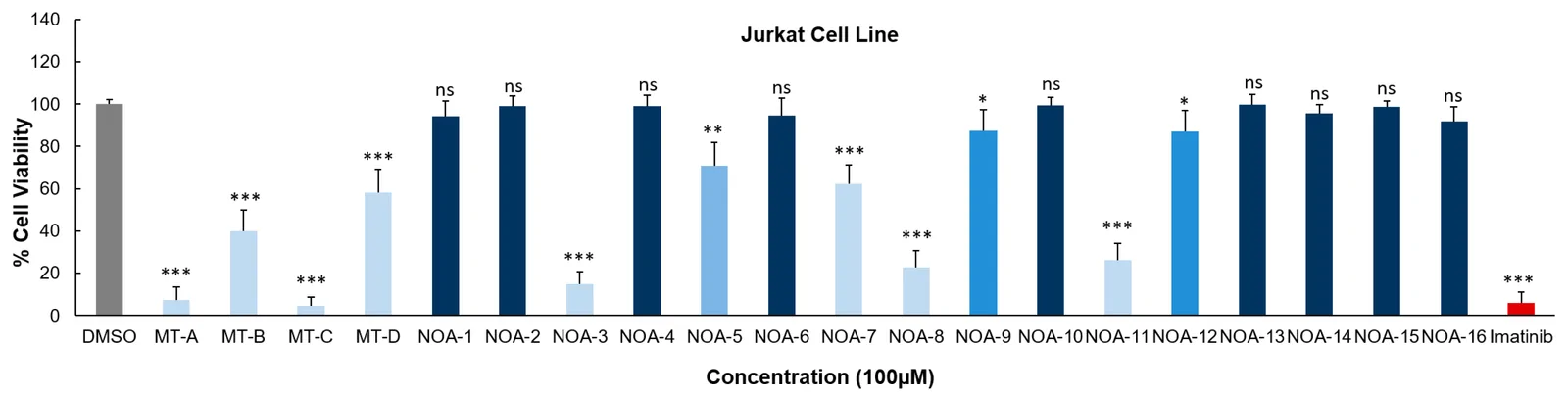
Cytotoxic activity of MT-A-D and NOA1-16 derivatives against Jurkat cells at 100 µM. Cell viability was determined using the MTT assay after 48 h of treatment. Data are presented as mean ± SD of three independent experiments (*n* = 3). Statistical significance was determined using one-way ANOVA followed by Dunnett’s multiple comparisons test. ns, not significant; * *p* < 0.05; ** *p* < 0.01; *** *p* < 0.001 versus the DMSO control group.

A distinct activity profile was observed in Jurkat cells (Figure 3). MT-A and MT-C retained moderate antiproliferative activity, whereas several NOA derivatives exhibited markedly improved potency. Among them, NOA-8 emerged as the most active compound with an IC_50_ value of 6.81 ± 1.08 µM, showing stronger activity than imatinib (12.53 ± 1.85 µM). NOA-3 and NOA-11 also demonstrated considerable cytotoxicity, with IC_50_ values of 14.85 ± 1.50 µM and 15.99 ± 1.49 µM, respectively. In contrast, NOA-1, NOA-4, NOA-7, NOA-9, and NOA-10 were essentially inactive.

**Figure 4:**
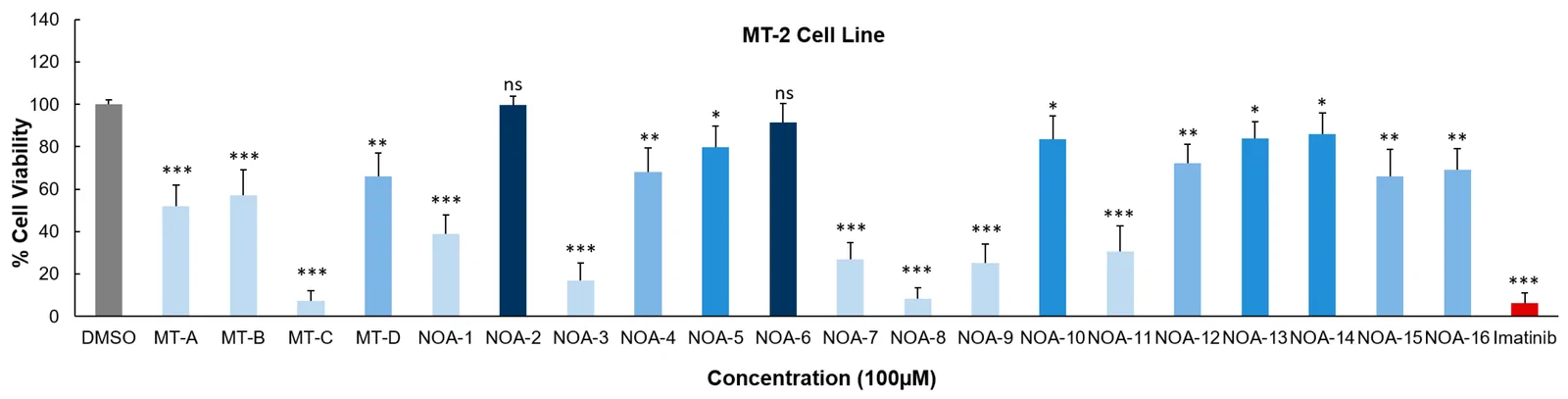
Cytotoxic activity of MT-A-D and NOA1-16 derivatives against MT-2 cells at 100 µM. Cell viability was determined using the MTT assay after 48 h of treatment. Data are presented as mean ± SD of three independent experiments (*n* = 3). Statistical significance was determined using one-way ANOVA followed by Dunnett’s multiple comparisons test. ns, not significant; * *p* < 0.05; ** *p* < 0.01; *** *p* < 0.001 versus the DMSO control group.

Similarly, evaluation in MT-2 cells demonstrated that the NOA derivatives possessed superior activity compared with the MT series (Figure 4). NOA-8 again showed the strongest cytotoxicity with an IC_50_ value of 2.48 ± 0.88 µM, representing substantially greater potency than imatinib (20.98 ± 3.22 µM). NOA-3, NOA-7, and NOA-11 also exhibited significant activity, with IC_50_ values of 11.60 ± 1.76 µM, 10.66 ± 1.25 µM, and 16.69 ± 1.62 µM, respectively. Among the MT derivatives, only MT-C displayed moderate inhibition (45.28 ± 6.38 µM), whereas MT-A, MT-B, and MT-D remained largely inactive.

To evaluate selectivity toward cancer cells, the most active compounds, MT-A, MT-C, NOA-3, and NOA-8, were further tested against healthy PBMCs. As shown in Table 1, all selected compounds exhibited IC_50_ values above 300 µM, indicating very low toxicity toward normal cells. In contrast, imatinib showed a significantly lower IC_50_ value against PBMCs (33.00 ± 5.08 µM).

### 3.3. Apoptosis Analysis

**Figure 5:**
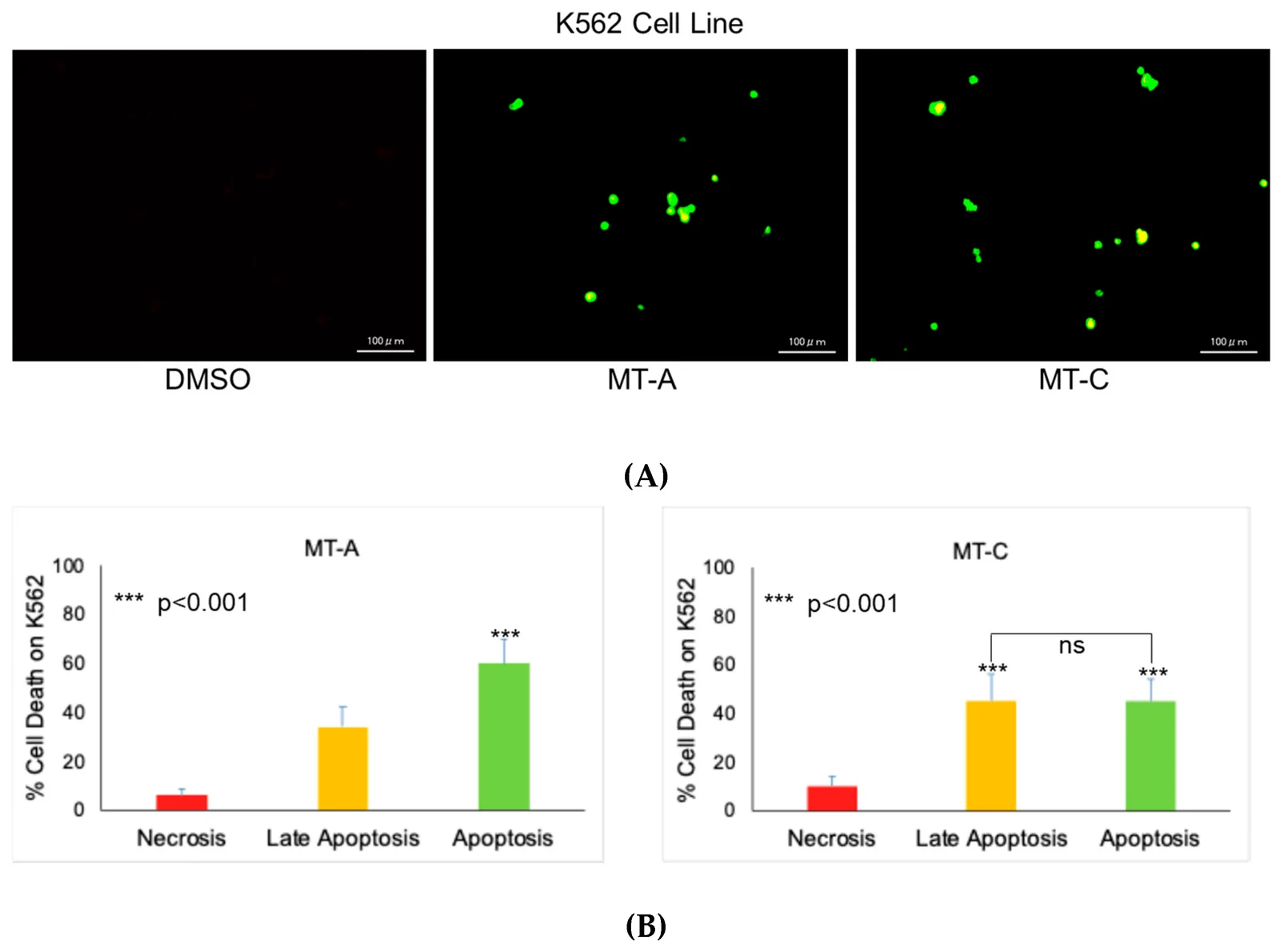
Apoptotic effects of MT-A and MT-C in K562 cells after 12 h exposure. (**A**) Representative fluorescence microscopy images illustrating early apoptotic (green), late apoptotic (yellow), and necrotic (red) cell populations. (**B**) Statistical evaluation of early apoptosis, late apoptosis, and necrosis percentages. Results are expressed as mean ± SD. *** *p* < 0.001 versus the control group, ns: non-significant.

To further investigate the mechanism underlying the observed cytotoxicity, apoptosis analyses were performed in K562 cells following 12 h treatment with MT-A and MT-C at their respective IC_50_ concentrations (Figure 5). Both compounds significantly induced apoptotic cell death. MT-A caused approximately 60% apoptosis together with 34% late apoptosis, whereas MT-C induced 45% apoptosis and 45% late apoptosis.

### 3.4. ABL TK Inhibition

**Figure 6:**
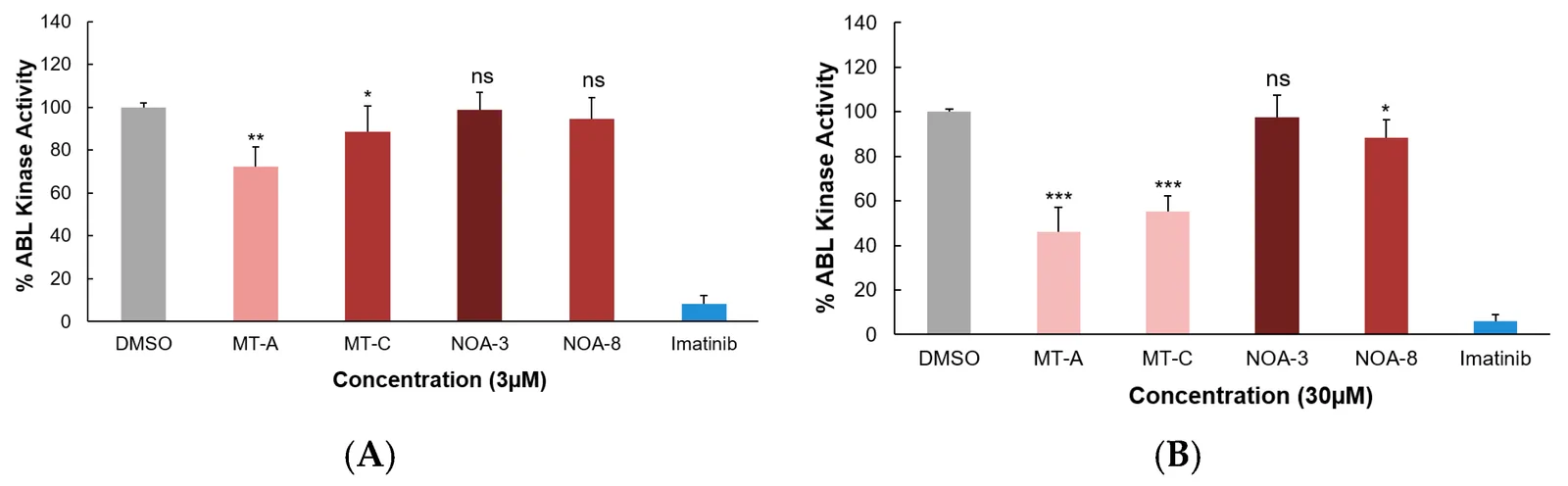
Effects of MT-A, MT-C, NOA-3, NOA-8, and imatinib on ABL kinase activity at 3 µM (**A**) and 30 µM (**B**). ABL kinase activity is expressed as a percentage relative to the DMSO control group. Data are presented as mean ± SD of three independent experiments (*n* = 3). Statistical significance was determined using one-way ANOVA followed by Dunnett’s multiple comparisons test. ns, not significant; * *p* < 0.05; ** *p* < 0.01; *** *p* < 0.001 versus the DMSO control group.

The inhibitory effects of the synthesized compounds on ABL TK activity were evaluated at both 3 µM and 30 µM to investigate whether their antiproliferative activities were associated with direct kinase inhibition. At 30 µM (Figure 6B), the inhibitory activities of MT-A and MT-C increased markedly, reducing ABL TK activity by 46.05% and 55.19%, respectively. NOA-3 remained inactive, while NOA-8 produced only a slight reduction in ABL TK activity despite reaching statistical significance. Imatinib exhibited potent inhibition at both concentrations. At 3 µM (Figure 6B), MT-A and MT-C exhibited modest inhibition of ABL TK activity, whereas NOA-3 and NOA-8 showed no significant inhibitory effect compared with the DMSO control. These findings indicate that ABL TK inhibition may contribute to the biological activity of MT-A and MT-C, whereas the pronounced antiproliferative activity of NOA-8 is unlikely to be primarily mediated through direct ABL kinase inhibition.

### 3.5. Molecular Docking Studies

To further investigate the binding modes of the active compounds within the kinase domain, molecular docking studies were performed using the ATP-binding pocket of ABL TK (PDB ID: 1IEP). The docking scores and MM-GBSA binding free energies are summarized in Table 2. Among the investigated ligands, imatinib exhibited the most favorable docking score (−14.792 kcal/mol), followed by MT-C (−5.262 kcal/mol) and MT-A (−4.809 kcal/mol). A similar trend was not observed for the MM-GBSA calculations, where imatinib displayed the lowest binding free energy (ΔG_bind_ = −114.19 kcal/mol), followed by MT-A (−29.30 kcal/mol) and MT-C (−15.94 kcal/mol).

**Figure 7:**
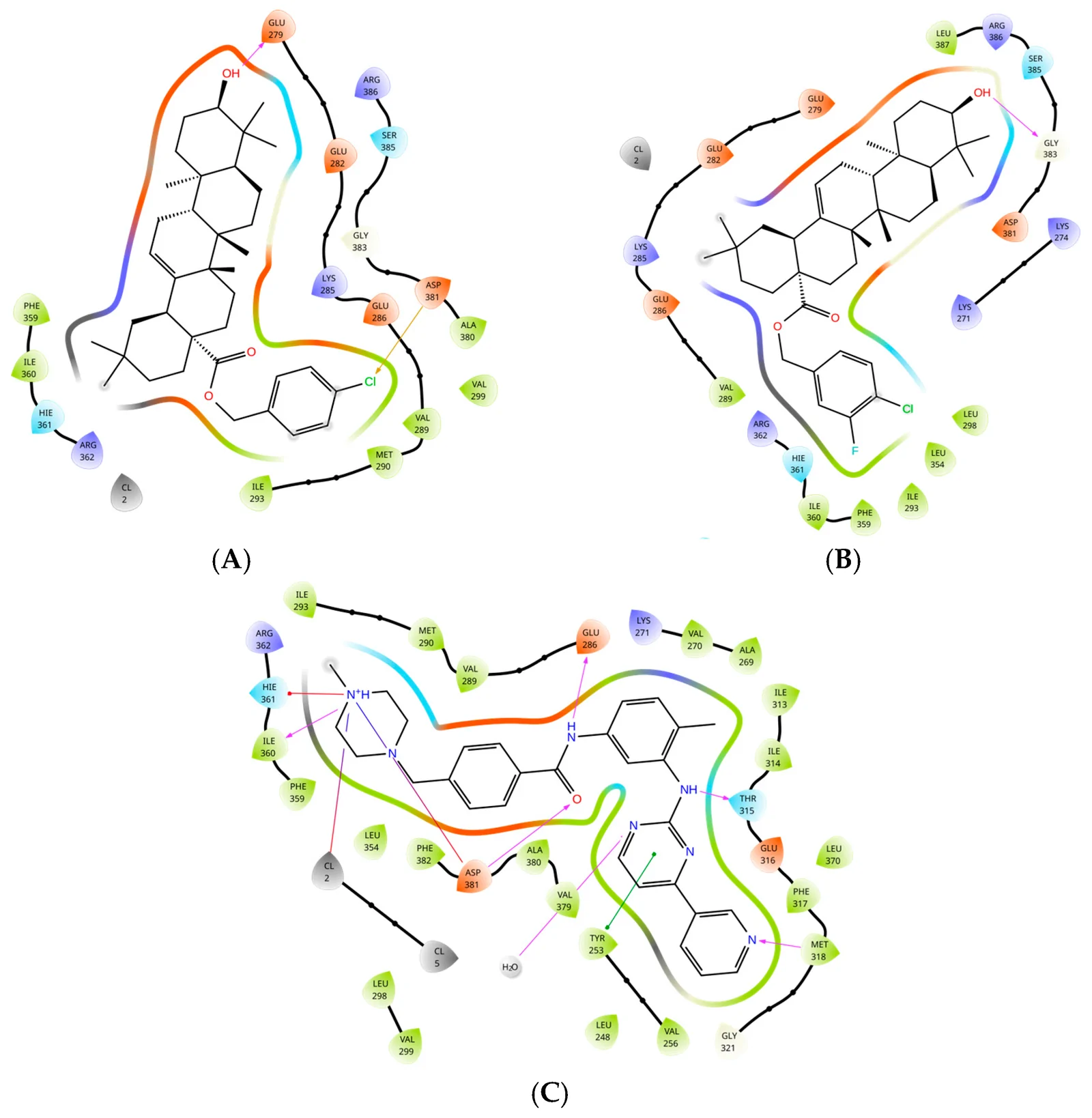
The 2D interaction diagrams of MT-A (**A**) MT-C (**B**) and imatinib (**C**) bound to ABL TK.

The two-dimensional (2D) interaction analysis showed that MT-A (Figure 7A) formed a hydrogen bond with Gly383 and a halogen bond between the chlorine atom and Asp381, while hydrophobic interactions were observed with Val289, Met290, Ala380, Ile293, Ile360, Phe359, and Leu298. MT-C (Figure 7B) established a hydrogen bond with Gly383 and hydrophobic contacts involving Leu387, Leu354, Ile293, Ile360, Phe359, Val289, and Leu298, with His361 and Arg362 located in close proximity to the ligand. On the other hand, imatinib (Figure 7C) established an extensive hydrogen-bonding network with His361, Glu286, Asp381, and Thr315, together with additional interactions involving Tyr253, Met318, Val299, Ile360, Arg362, Lys271, Val256, Val289, and Met290.

**Figure 8:**
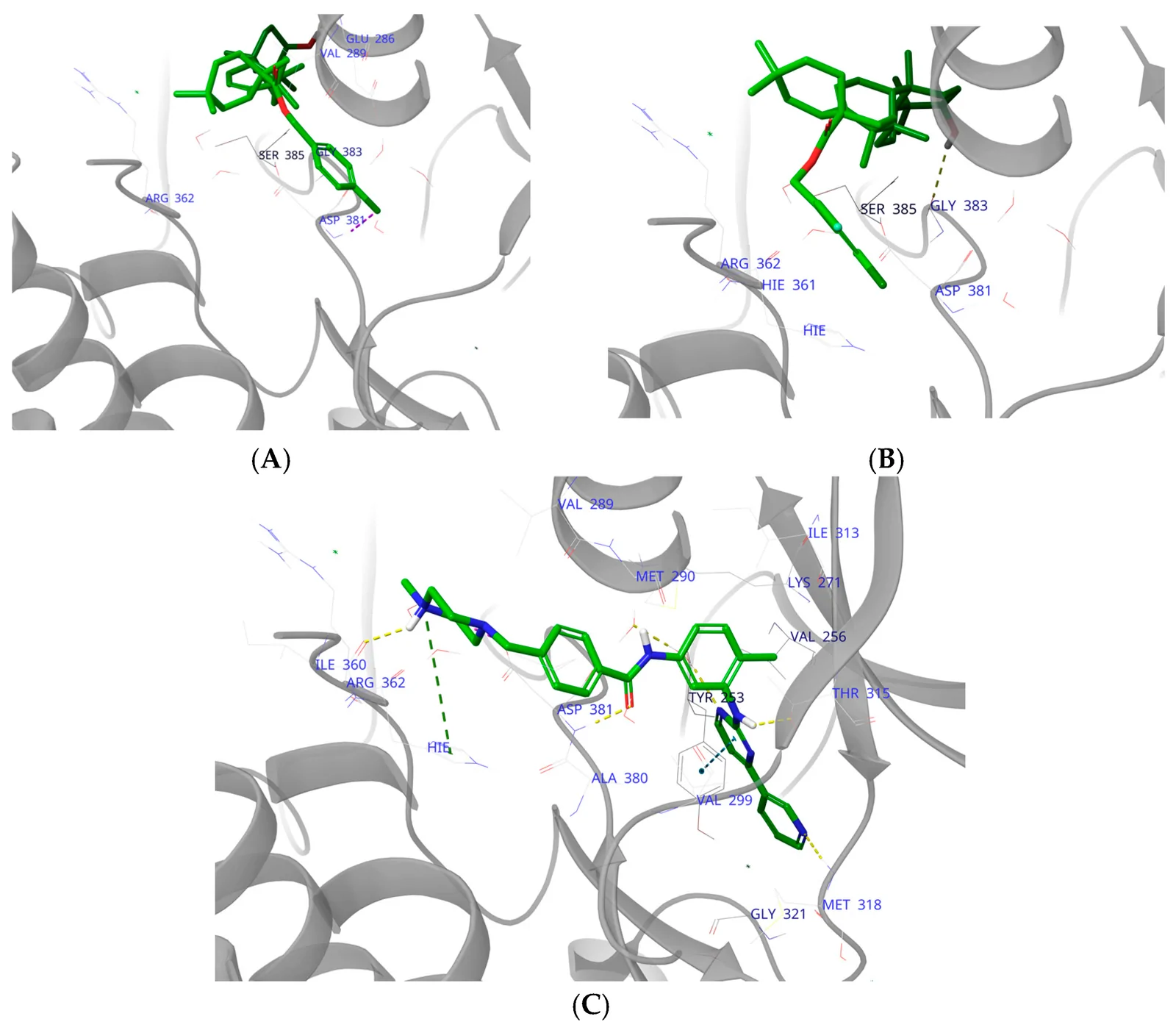
3D representation of the binding orientations of MT-A (**A**), MT-C (**B**), and imatinib (**C**) within the active site of ABL TK.

The three-dimensional (3D) binding poses further demonstrated that all ligands occupied the ATP-binding cavity but adopted distinct orientations. MT-A (Figure 8A) and MT-C (Figure 8B) occupied a more confined region of the pocket while maintaining the interaction patterns identified in the 2D analysis. Imatinib (Figure 8C) extended across the binding cleft, allowing interactions with residues distributed throughout the active site.

To further investigate the stability of the docked complexes, 100 ns MD simulations were performed for MT-A, MT-C, and the reference inhibitor imatinib in complex with ABL TK. The stability of each complex was evaluated by monitoring the protein and ligand root mean square deviation (RMSD), residue fluctuations (RMSF), and protein–ligand interaction profiles throughout the simulation.

**Figure 9:**
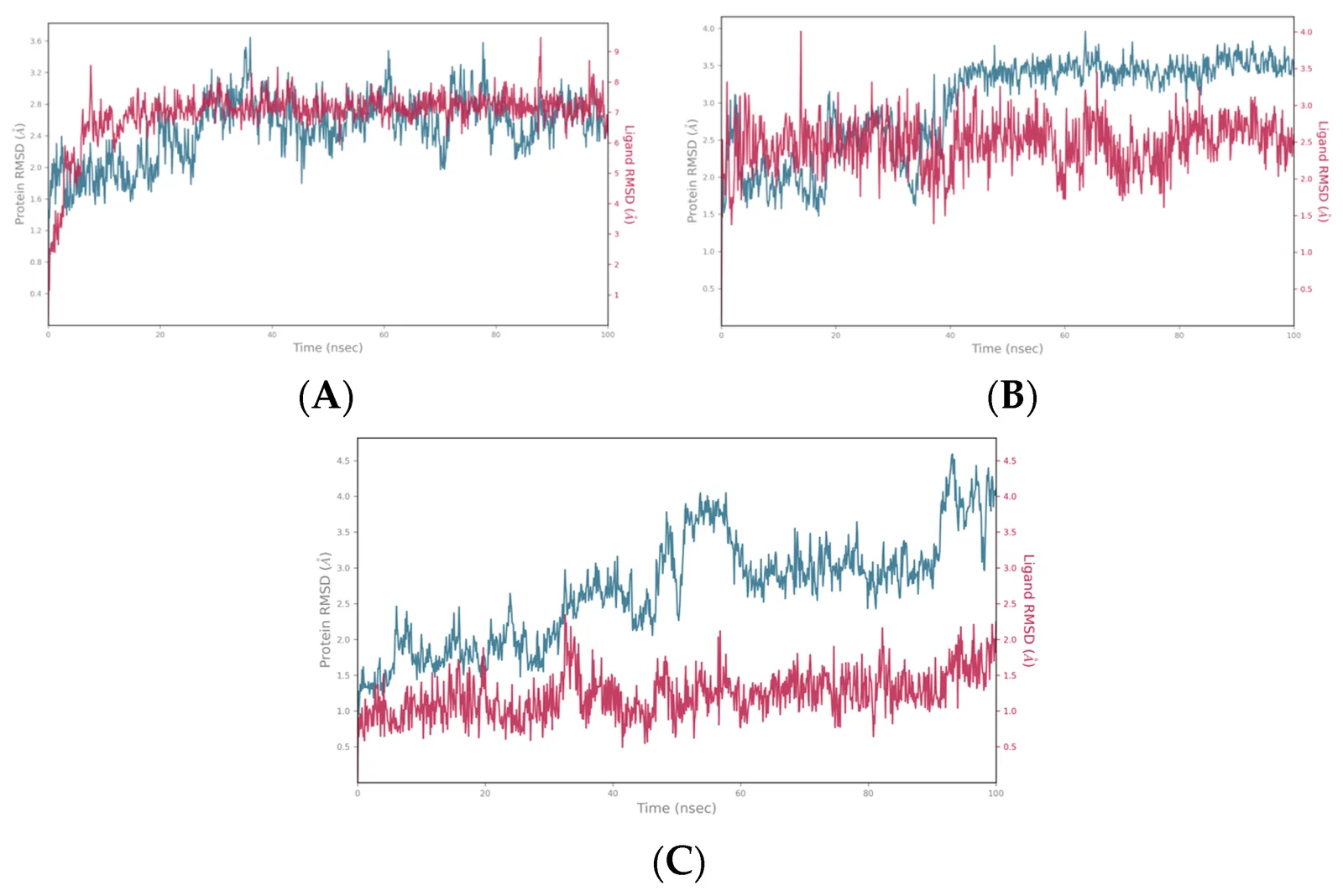
Protein-ligand RMSD plots of MT-A (**A**), MT-C (**B**), and imatinib (**C**) during 100 ns MD simulations.

As shown in Figure 9A–C, all three complexes remained stable during the 100 ns simulations. The protein backbone RMSD reached equilibrium after the initial equilibration period and remained relatively constant thereafter. The ligand RMSD values also remained within an acceptable range, indicating that MT-A, MT-C, and imatinib remained bound within the ATP-binding pocket throughout the simulations.

**Figure 10:**
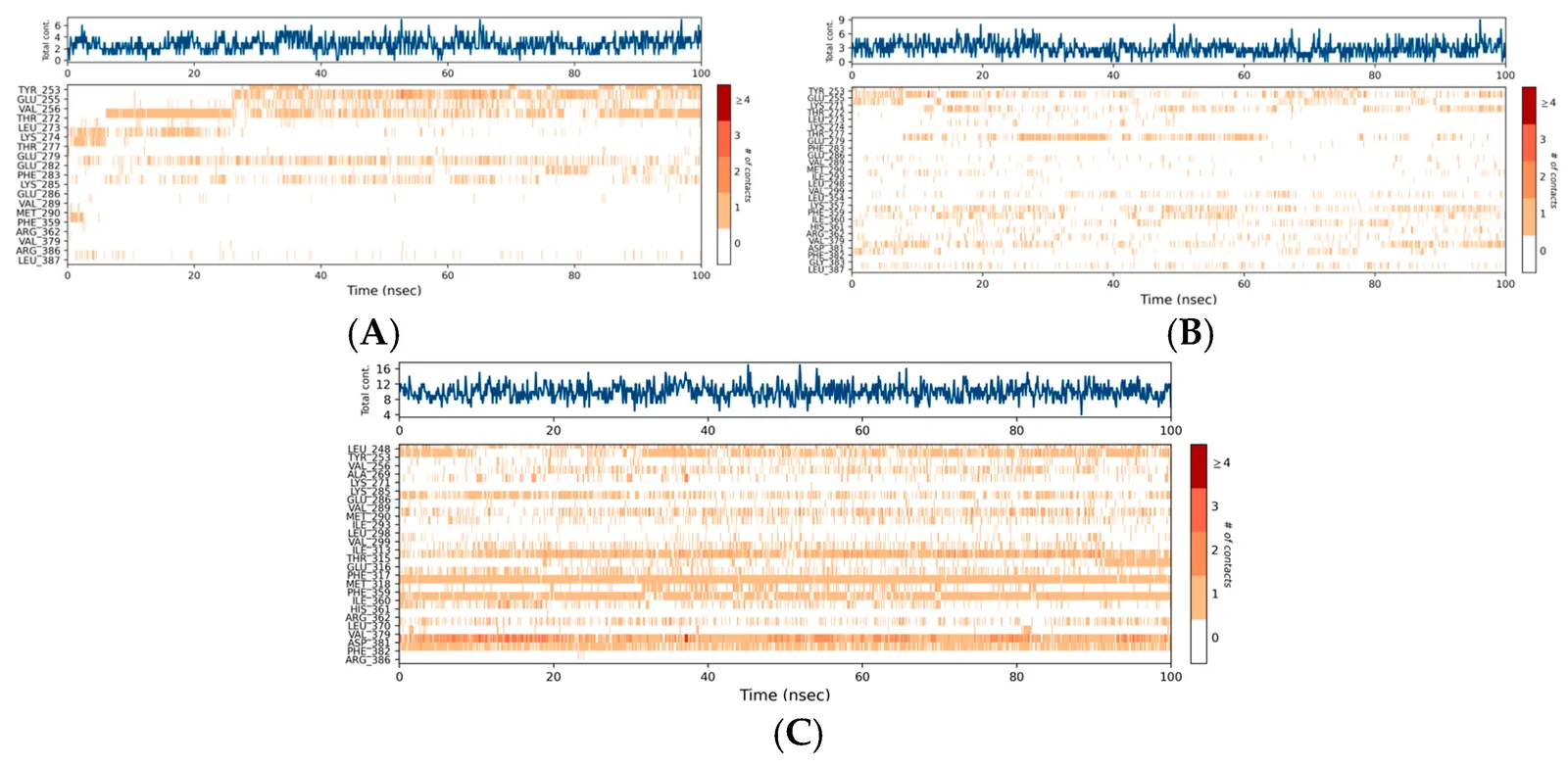
Protein-ligand contact timelines of MT-A (**A**), MT-C (**B**), and imatinib (**C**).

Protein-ligand interaction analysis demonstrated persistent contacts between the ligands and residues lining the ABL TK (Figure 10A–C). For MT-A, the most frequently observed interactions involved Glu255, Thr272, Glu282, Lys285, and Leu387. MT-C exhibited persistent contacts with Glu255, Glu279, Phe359, Ile360, Asp381, Phe382, and Leu387, whereas imatinib maintained interactions with Glu255, Thr272, Phe359, Ile360, Asp381, Phe382, and Leu387 during the simulation.

**Figure 11:**
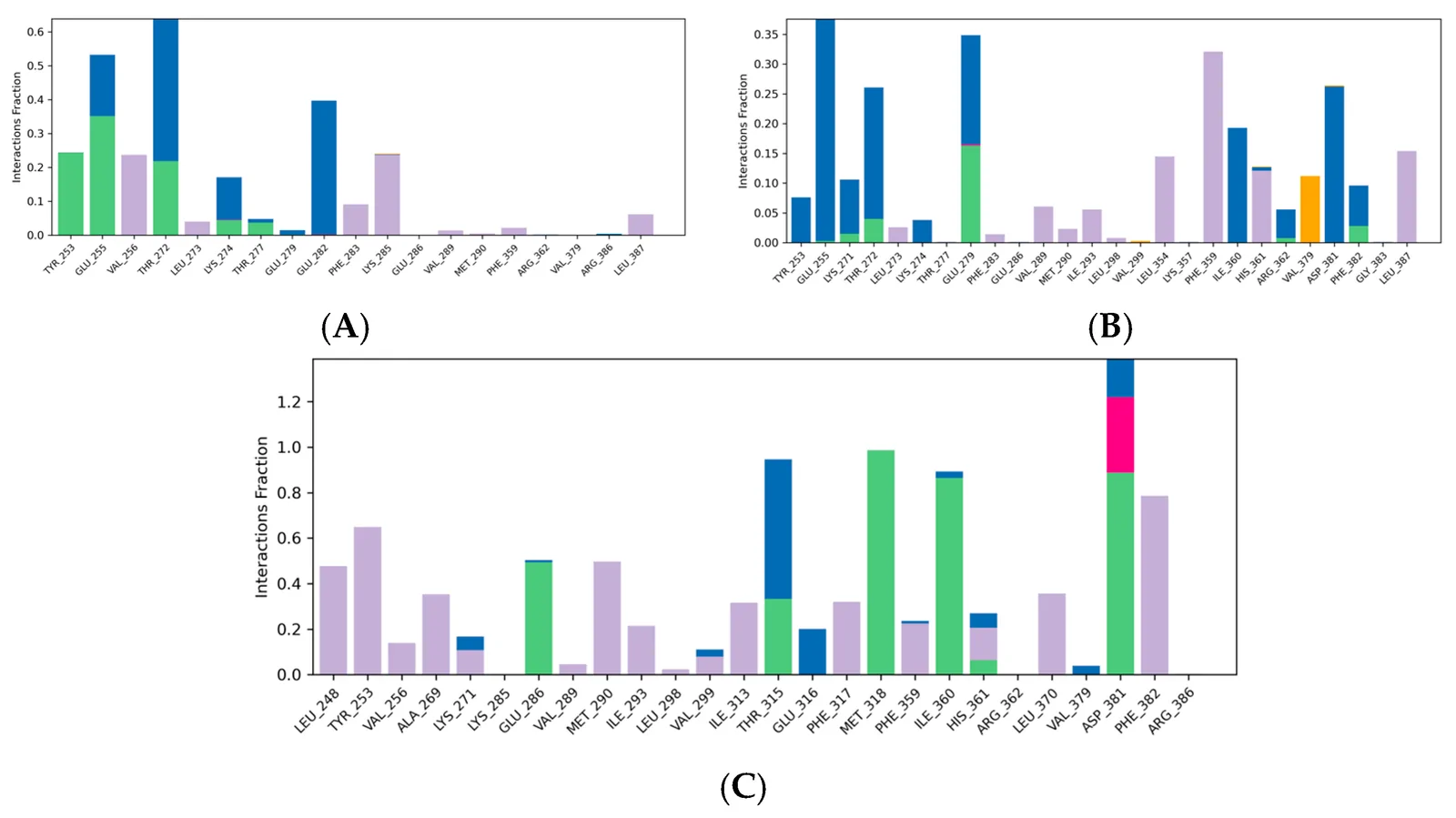
Protein–ligand interaction occupancy histograms of MT-A (**A**), MT-C (**B**), and imatinib (**C**).

The interaction occupancy plots (Figure 11A–C) further confirmed that the dominant contacts observed in the docking models were preserved throughout the MD simulations. In addition to hydrogen bonds, hydrophobic and water-mediated interactions contributed to stabilization of the ligand–protein complexes over the course of the trajectories.

**Figure 12:**
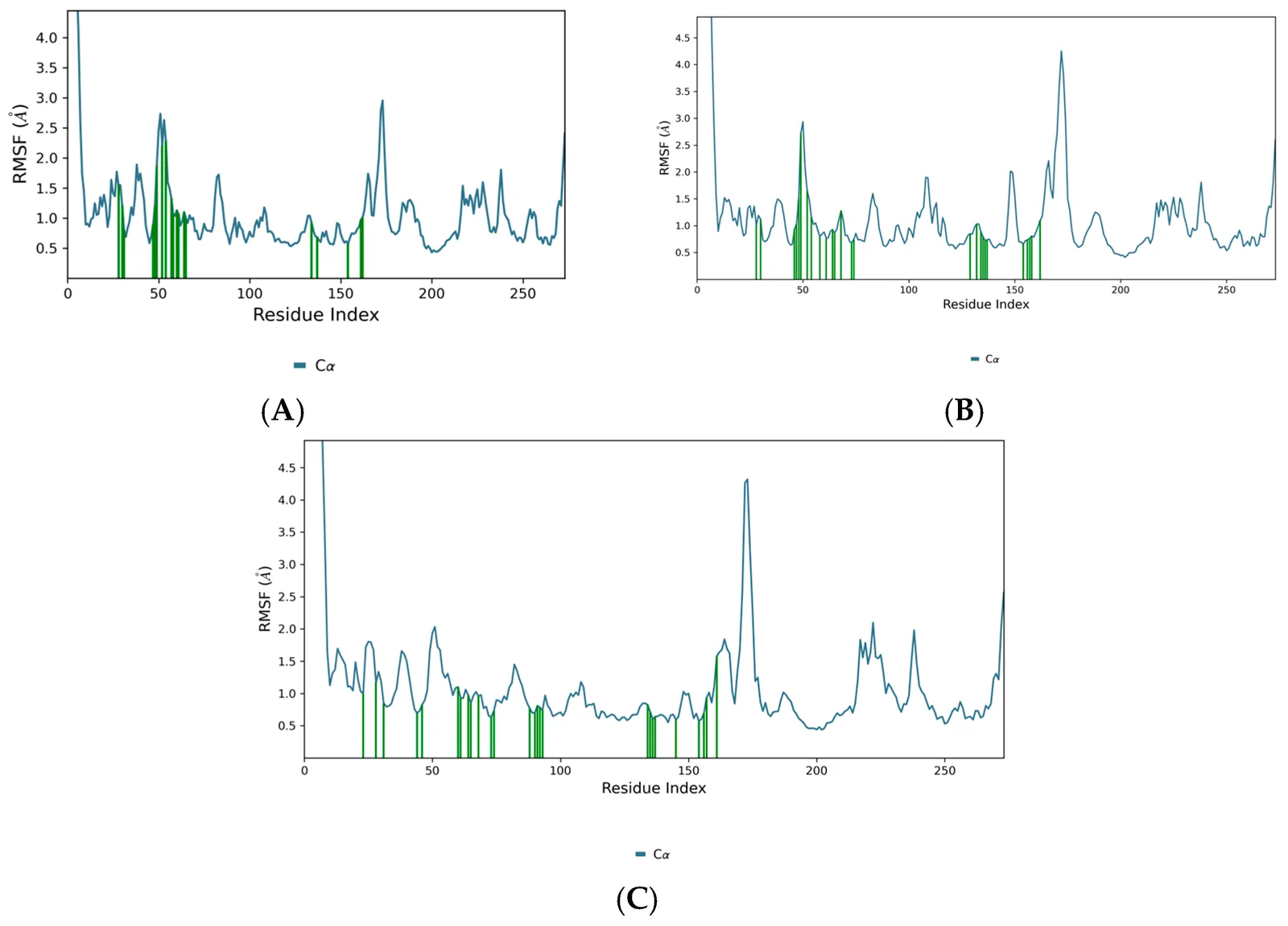
Protein RMSF profiles for the ABL complexes with MT-A (**A**), MT-C (**B**), and imatinib (**C**).

Protein RMSF analysis revealed generally low residue fluctuations throughout the kinase domain, with higher flexibility observed only in terminal and loop regions, while residues constituting the ligand-binding pocket remained comparatively stable during the simulations (Figure 12A–C).

**Figure 13:**
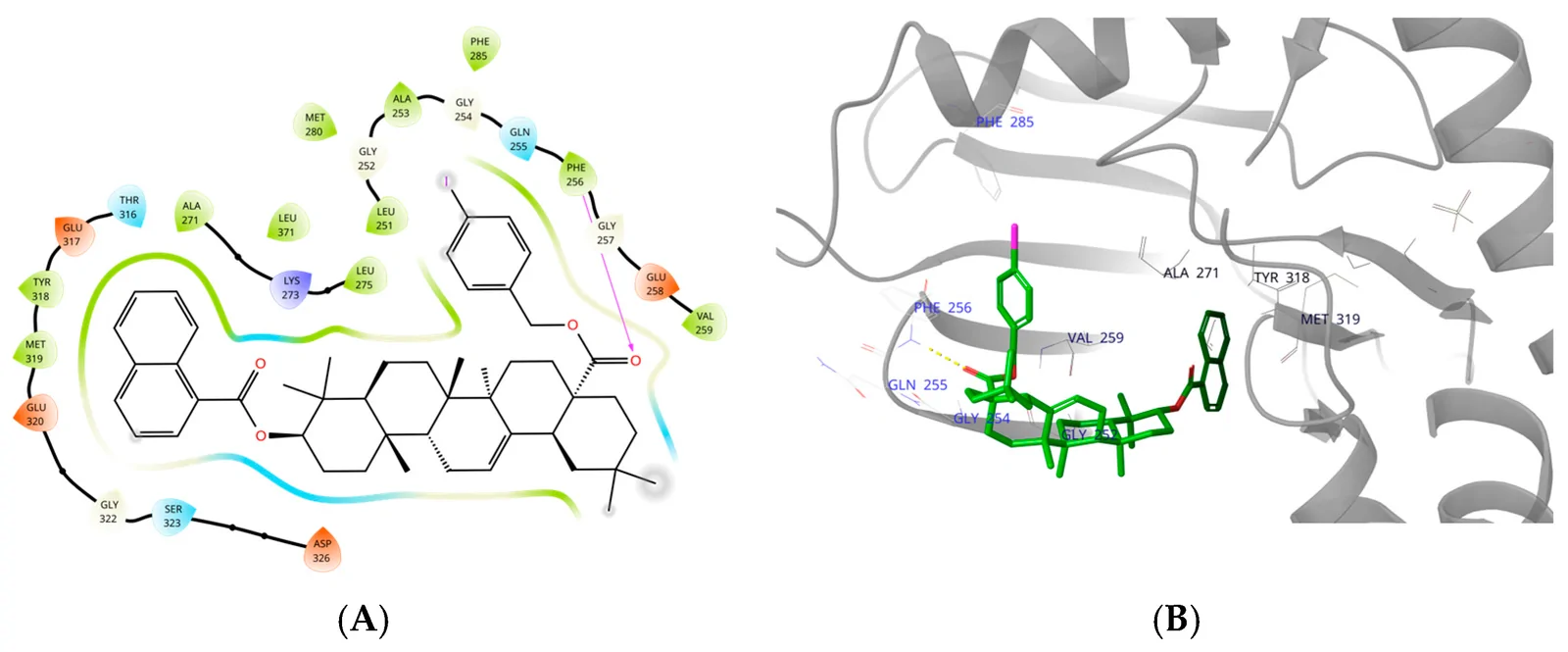
2D (**A**) and 3D (**B**) representation of the binding orientations of NOA-8 within the LCK ATP-binding pocket.

To further investigate the potential mechanism underlying the selective activity of NOA-8 against Jurkat and MT-2 cells, molecular docking was performed against human LCK kinase (PDB ID: 3LCK) [31]. NOA-8 exhibited a Glide docking score of −6.50 kcal/mol and an estimated MM/GBSA binding free energy (ΔG_bind_) of −54.83 kcal/mol (Table 2), suggesting favorable binding affinity toward the LCK ATP-binding pocket. The predicted binding mode revealed a hydrogen bond with Phe256, together with hydrophobic interactions involving Gln255, Val259, Ala271, Met319, and Phe285 (Figure 13A,B).

Preliminary in silico absorption, distribution, metabolism, and elimination (ADME) properties of the two most active ABL-targeting compounds (MT-A and MT-C) were predicted using the SwissADME web server. The calculated physicochemical and pharmacokinetic parameters are summarized in Table 3. Both compounds satisfied the Veber criterion and showed no PAINS alerts, suggesting acceptable molecular flexibility and a low probability of nonspecific assay interference. However, both derivatives exhibited high molecular weight (>500 Da), high lipophilicity (consensus LogP > 8), low predicted gastrointestinal (GI) absorption, and poor aqueous solubility, resulting in two Lipinski rule violations. These findings indicate that although MT-A and MT-C possess promising biological activity, further structural optimization will be required to improve their predicted pharmacokinetic properties.

## 4. Discussion

The MT-A-D derivatives were synthesized through esterification of the C-28 carboxylic acid group of OA with the corresponding halogenated benzyl bromides. In the ^1^H NMR spectra, characteristic singlet and multiplet signals appearing between 0.50–1.85 ppm corresponded to the methyl and methylene protons of the pentacyclic triterpenoid skeleton, indicating preservation of the OA core structure. The olefinic proton at C-12 was detected at 5.27–5.35 ppm in all derivatives, demonstrating that the C12-C13 double bond remained intact during the reaction sequence. Two doublets observed at 4.90–5.30 ppm (J ≈ 11–13 Hz) were assigned to the benzylic –OCH_2_– protons, supporting successful esterification at the C-28 position. Aromatic proton patterns varied according to the halogen substituent. MT-A displayed the characteristic pattern of a para-disubstituted benzene ring, whereas MT-B exhibited downfield aromatic shifts due to the deshielding effect of iodine. In MT-C, fluorine substitution produced more complex splitting patterns as a result of additional spin-spin coupling, while MT-D showed further downfield shifts caused by the electron-withdrawing effect of two chlorine atoms.

The ^13^C NMR spectra further supported the proposed structures. Signals within the 14–47 ppm range corresponded to aliphatic carbons, whereas the benzylic –OCH_2_– carbon resonated at approximately 64–65 ppm. Aromatic and olefinic carbons appeared between 118–145 ppm, and the ester carbonyl carbon was observed at 177–178 ppm. HRMS analyses were fully consistent with the calculated molecular formulas, confirming the successful synthesis of the targeted benzyl ester derivatives.

The observed variations in the aromatic proton and carbon resonances were consistent with the different electronic environments generated by the halogen substituents. Although these changes were relatively small because the substitutions were confined to the benzyl moiety, they confirmed the successful introduction of structurally distinct C-28 benzyl ester derivatives.

For the NOA-1-16 series, the ^1^H NMR spectra similarly demonstrated preservation of the OA skeleton through characteristic methyl proton signals between 0.5–1.2 ppm. The olefinic proton resonance detected at 5.28–5.34 ppm indicated retention of the C12-C13 double bond throughout the synthetic pathway. The benzylic methylene protons of the ester moiety appeared as two distinct doublets at 4.95–5.12 ppm (J ≈ 12–13 Hz), while the oxygenated proton at the C-3 position produced a dd-type signal between 4.6–4.9 ppm. Minor chemical shift variations reflected the electronic properties of the aromatic substituents introduced at this position.

In the ^13^C NMR spectra, the ester carbonyl carbon resonated near 177 ppm in all derivatives, while the aromatic ester carbonyl signals were detected between 165–167 ppm. The oxygenated C-3 carbon consistently appeared at approximately 80–81 ppm. Methoxy-substituted derivatives displayed characteristic signals at 55–58 ppm corresponding to the methoxy carbons, whereas these signals were absent in phenyl- and naphthyl-containing compounds lacking methoxy groups. Distinct aromatic proton patterns were also observed depending on the substitution type; para-disubstituted aromatic systems generated characteristic doublets, while naphthyl derivatives exhibited broader multiplet signals between 7.3–8.2 ppm. Mass data were in full agreement with the proposed molecular structures, confirming successful functionalization at both the C-3 and C-28 positions.

The aromatic acyl substituents generated slightly different electronic environments around the C-3 ester functionality, which was reflected by minor variations in the aromatic and carbonyl resonances in the ^1^H and ^13^C NMR spectra. These variations were consistent with the structural diversity of the introduced aromatic groups, whereas the characteristic resonances of the OA skeleton remained essentially unchanged.

Natural products have long attracted considerable attention because of their structural diversity and anticancer potential [36]. OA, a naturally occurring pentacyclic triterpenoid, exhibits various biological activities; however, its poor aqueous solubility and limited bioavailability restrict its therapeutic applicability. Therefore, structural modifications at the C-3 and C-28 positions have frequently been employed to improve its pharmacological properties and target selectivity [19]. Previous studies demonstrated that OA derivatives can inhibit proliferation and induce apoptosis in K562 CML cells [37]. In our earlier work, OA benzyl ester derivatives showed promising anti-ABL activity compared with imatinib, while gypsogenin-based compounds also exhibited encouraging anti-leukemic potential [38].

Based on these findings, a new series of OA-derived compounds was designed and synthesized. Among the MT derivatives, MT-A and MT-C exhibited the most pronounced antiproliferative activity against K562 cells. In contrast, MT-B and MT-D displayed limited activity. These findings suggest that the 4-chlorobenzyl and 4-chloro-3-fluorobenzyl substituents may contribute favorably to cytotoxic activity, whereas bulkier or less compatible substituents, such as 4-iodobenzyl and 3,4-dichlorobenzyl groups, may reduce biological efficacy [39].

The NOA derivatives generally exhibited weaker activity against K562 cells. While the majority of the NOA derivatives-maintained cell viability above 80%, NOA-10, NOA-8, and NOA-3 displayed relatively stronger inhibitory effects compared with the remaining derivatives. The improved activity observed for these compounds may be associated with the presence of trimethoxybenzoyl, naphthoyl, and benzoyl moieties at the C-3 position, which could enhance intracellular interactions and antiproliferative potential [40]. IC_50_ analyses revealed that imatinib exhibited the highest activity against K562 cells. In contrast, MT-A and MT-C displayed lower IC_50_ values than the other OA derivatives, whereas the majority of the NOA derivatives showed weaker activity. Overall, these findings indicate that the antiproliferative activity of OA derivatives is highly dependent on the nature and position of the introduced substituents, which is consistent with previous SAR studies on triterpenoid derivatives [41,42].

In HL-60 cells, the MT derivatives exhibited markedly higher cytotoxic activity than the NOA series, suggesting that the presence of halogenated benzyl substituents within the MT scaffold plays a key role in enhancing anti-leukemic potency in this cell line. On the other hand, Jurkat cells were more sensitive to the NOA derivatives than to the MT series. Among these compounds, NOA-8 displayed the highest activity, while NOA-3 and NOA-11 also showed considerable cytotoxic effects. Several other NOA derivatives, however, were inactive under the tested conditions. The superior activity of NOA-8, which bears a naphthoyl moiety, suggests that this structural feature may promote favorable molecular interactions contributing to enhanced activity against T-cell leukemia.

A similar trend was observed in MT-2 cells, where the NOA derivatives generally outperformed the MT series. NOA-8 again emerged as the most potent compound, followed by NOA-3, NOA-7, and NOA-11, whereas most MT derivatives exhibited only weak activity. These findings indicate that structural diversification at the C-3 position, particularly through the incorporation of aromatic acyl substituents, substantially influences cytotoxic activity against T-cell leukemia cells. The marked difference between the activities observed in leukemia cells and healthy PBMCs suggests favorable selectivity profiles for these derivatives. Particularly, NOA-3 and NOA-8 demonstrated strong antiproliferative activity in Jurkat and MT-2 cells while remaining essentially non-toxic toward PBMCs. Similarly, MT-A and MT-C displayed potent effects against HL-60 and Jurkat cells without significant toxicity toward healthy cells. These findings indicate that the selected compounds may possess advantageous therapeutic windows and support their potential as promising anti-leukemic candidates.

Although NOA-8 did not exhibit significant ABL TK inhibition under experimental conditions, it demonstrated the strongest antiproliferative activity among the NOA derivatives against Jurkat and MT-2 leukemia cell lines while exhibiting negligible cytotoxicity toward PBMCs. Therefore, NOA-8 was identified as a promising anti-leukemic lead compound based on its potent biological activity and favorable selectivity profile rather than its ABL tyrosine kinase inhibitory activity. These findings suggest that its anti-leukemic activity may involve molecular mechanisms other than direct ABL TK inhibition, consistent with previous reports demonstrating that OA and its derivatives modulate multiple intracellular signaling pathways involved in cancer cell proliferation and apoptosis [14,16]. Further mechanistic studies are warranted to elucidate its precise mode of action.

Previous studies have mainly focused on structural modification of OA at either the C-3 hydroxyl group or the C-28 carboxyl group to improve its anticancer activity and pharmacological properties [14,15,19]. In the present study, OA was first converted into C-28 benzyl ester derivatives (MT series), followed by O-acylation of the C-3 hydroxyl group to generate the NOA series. This design enabled a direct comparison between C-28-modified derivatives and their corresponding C-3/C-28 doubly modified analogues under identical biological evaluation conditions. Therefore, the present study extends previously reported structure–activity relationships by evaluating the influence of sequential C-28 and C-3 modifications on anti-leukemic activity across different leukemia cell lines and provides additional insight for the rational design of OA-based anti-leukemic agents.

The obtained biological data further support previous reports indicating that derivatization of OA at the C-3 and C-28 positions can substantially influence anticancer activity [43,44]. Previous studies have shown that both the site of modification and the chemical nature of the introduced substituent may alter the cytotoxic profile of OA derivatives across different cancer cell lines [15,16,19,43,45]. In the present study, comparison of the two derivative series revealed distinct, cell line-dependent activity patterns. Among the C-28-modified derivatives, MT-A and MT-C, bearing 4-chlorobenzyl and 4-chloro-3-fluorobenzyl groups, respectively, exhibited the most pronounced antiproliferative effects against K562 and HL-60 cells, whereas MT-B and MT-D were considerably less active against K562 cells. These findings indicate that the presence of a halogenated benzyl group alone was not sufficient to enhance activity and that the type, number, and position of the halogen substituents may contribute to the observed biological response [15,19]. Selected C-3 aromatic acyl derivatives showed greater activity against Jurkat and MT-2 cells. NOA-8 exhibited the most favorable antiproliferative profile among the C-3-modified compounds, while NOA-3 and NOA-11 also demonstrated promising activity. Taken together, these results suggest that the antiproliferative effects of the synthesized derivatives were influenced by the modification site, substituent characteristics, and cell type [14,15,19]. Nevertheless, because the present compound library contains a limited number of structurally related derivatives, definitive SAR cannot yet be established. Therefore, these findings should be regarded as preliminary SAR observations that may guide the future design and optimization of OA-based antileukemic agents [19,43]. While increased lipophilicity may improve cellular permeability, excessive hydrophobicity or steric bulk can negatively affect aqueous solubility and bioavailability, ultimately limiting biological efficacy [45]. Therefore, the overall activity of OA derivatives appears to depend on achieving an optimal balance between steric, electronic, and physicochemical properties. In addition, although imatinib exhibited strong antiproliferative activity, it also demonstrated notable toxicity toward PBMCs, which is consistent with clinical reports describing treatment-related adverse effects associated with imatinib therapy [46].

Apoptosis analyses demonstrated that MT-A and MT-C induced pronounced apoptotic cell death in K562 cells. Although MT-A exhibited a higher level of early apoptosis, both compounds induced substantial levels of apoptosis and late apoptosis. Necrotic cell populations remained low in both treatment groups, indicating that the observed cytotoxicity was predominantly associated with apoptosis. These findings suggest that MT-A and MT-C exert their antiproliferative effects mainly through apoptosis-mediated mechanisms.

Constitutive activation of BCR-ABL TK is the primary molecular driver of CML, and inhibition of its kinase activity remains the cornerstone of targeted therapy [47]. ATP-competitive TKIs, including imatinib, nilotinib, dasatinib, bosutinib, and ponatinib, have substantially improved the clinical management of CML by suppressing BCR-ABL-mediated signaling pathways responsible for uncontrolled proliferation, resistance to apoptosis, and enhanced cell survival [47,48,49,50,51,52,53,54,55,56]. More recently, asciminib has introduced an alternative therapeutic strategy by allosterically targeting the myristoyl-binding pocket of ABL kinase rather than the ATP-binding site [48,49,57,58]. Despite these advances, resistance mutations, kinase-independent signaling, and treatment intolerance remain important clinical challenges, highlighting the need for novel chemical scaffolds with distinct mechanisms of action [52,53,54,55,56,57,58].

In the present study, the synthesized compounds were evaluated for their ability to inhibit ABL TK. Among the tested molecules, MT-A and MT-C demonstrated moderate inhibitory activity, whereas NOA-3 and NOA-8 exhibited little or no direct inhibition of ABL TK. Interestingly, the kinase inhibition profile did not fully correlate with the cytotoxicity results. Although NOA-8 was one of the most potent compounds against Jurkat and MT-2 leukemia cells, it showed negligible inhibition of ABL TK. The moderate kinase inhibition observed for MT-A and MT-C may contribute to their biological activity, but it is unlikely to fully account for their cytotoxic effects, indicating that additional molecular mechanisms are likely involved [59].

To gain mechanistic insight into these findings, molecular docking and MD simulations were performed. The computational analyses indicated that MT-A and MT-C can be favorably accommodated within the ABL ATP-binding pocket, suggesting that ABL inhibition may represent one component of their mechanism of action in CML cells. On the other hand, NOA-8 exhibited pronounced antiproliferative activity against the T-cell leukemia cell lines Jurkat and MT-2, despite showing negligible inhibition of ABL. This discrepancy suggested that alternative signaling pathways may be involved in its mechanism of action. Because LCK is a lymphocyte-specific Src family kinase that plays a pivotal role in T-cell receptor signaling, proliferation, and survival, and its aberrant activation has been implicated in T-cell leukemogenesis [60,61,62], LCK was investigated as a potential molecular target. Computational analyses indicated that NOA-8 can be favorably accommodated within the ATP-binding pocket of LCK. These findings suggest that LCK inhibition may represent a plausible mechanism underlying the selective activity of NOA-8 against T-cell leukemia cells. Nevertheless, because this conclusion is currently based solely on computational evidence, direct biochemical kinase assays and cellular target-engagement studies will be necessary to confirm LCK as the molecular target of NOA-8. These findings identify MT-A and MT-C as promising ABL-associated lead compounds and NOA-8 as a potential LCK-targeting scaffold for T-cell leukemia. Although preliminary ADME predictions revealed suboptimal pharmacokinetic properties for MT-A and MT-C, further medicinal chemistry optimization may improve their developability while preserving their favorable biological activities.

## 5. Conclusions

In this study, a series of novel OA derivatives were successfully synthesized and evaluated for their anti-leukemic activities. Biological studies demonstrated that the antiproliferative effects of the compounds were strongly dependent on both the structural modifications and the leukemia cell type. Among the tested compounds, MT-A exhibited remarkable activity against HL-60 cells, whereas NOA-8 showed the most potent effects against Jurkat and MT-2 cells, surpassing imatinib in these models. Moreover, NOA-3 and NOA-11 also demonstrated promising anti-leukemic activity. Importantly, the most active compounds displayed minimal toxicity toward healthy PBMCs, indicating favorable selectivity profiles. Apoptosis and ABL TK inhibition studies suggested that the biological activities of the compounds may involve apoptosis-mediated and multi-target mechanisms rather than strong direct ABL TK inhibition alone. Computational analyses complemented the biological findings by providing mechanistic insights into the observed antiproliferative activities. The combined docking and MD results supported ABL as a relevant target for MT-A and MT-C and identified LCK as a possible alternative target for NOA-8, thereby guiding future biochemical validation and target-based optimization. Overall, these findings highlight OA derivatives, particularly MT-A, MT-C, NOA-3 and NOA-8, as promising lead candidates for the development of new anti-leukemic agents.

## Figures and Tables

**Table 1:** The IC_50_ values of MT-A-D and NOA1-16 against K562, HL-60, Jurkat, and MT-2 cells, and PBMCs.

Compound	IC_50_ Values (µM)				
	K562	HL-60	Jurkat	MT-2	PBMCs
**MT-A**	80.03 ± 10.26	4.17 ± 0.65	38.90 ± 4.26	>100	>300
**MT-B**	>100	9.74 ± 1.08	66.07 ± 8.02	>100	-
**MT-C**	72.60 ± 8.73	16.36 ± 2.41	33.33 ± 3.51	45.28 ± 6.38	>300
**MT-D**	>100	35.02 ± 5.19	>100	>100	-
**NOA-1**	>100	90.54 ± 8.74	>100	26.27 ± 4.41	-
**NOA-2**	>100	>100	>100	>100	-
**NOA-3**	>100	45.10 ± 4.16	14.85 ± 1.50	11.60 ± 1.76	>300
**NOA-4**	>100	83.69 ± 10.03	>100	>100	-
**NOA-5**	>100	>100	>100	>100	-
**NOA-6**	>100	>100	>100	>100	-
**NOA-7**	>100	>100	>100	10.66 ± 1.25	-
**NOA-8**	>100	>100	6.81 ± 1.08	2.48 ± 0.88	>300
**NOA-9**	>100	>100	>100	27.71 ± 4.84	-
**NOA-10**	>100	89.85 ± 9.54	>100	>100	-
**NOA-11**	>100	>100	15.99 ± 1.49	16.69 ± 1.62	-
**NOA-12**	>100	>100	>100	>100	-
**NOA-13**	>100	>100	>100	>100	-
**NOA-14**	>100	>100	>100	>100	-
**NOA-15**	>100	>100	>100	>100	-
**NOA-16**	>100	>100	>100	>100	-
**Imatinib**	7.28 ± 1.64	13.34 ± 1.92	12.53 ± 1.85	20.98 ± 3.22	33.00 ± 5.08

**Table 2:** Docking scores and MM/GBSA binding free energies of MT-A, MT-C, and imatinib with ABL kinase and NOA-8 with LCK kinase.

Compound	ABL		LCK	
	Docking Score (kcal/mol)	MM-GBSA Δ*G*_bind_ (kcal/mol)	Docking Score (kcal/mol)	MM-GBSA Δ*G*_bind_ (kcal/mol)
**MT-A**	−4.809	−29.30	-	-
**MT-C**	−5.262	−15.94	-	-
**NOA-8**	-	-	−6.50	−54.83
**Imatinib**	−14.792	−114.19	-	-

**Table 3:** Predicted ADME properties of MT-A and MT-C calculated using SwissADME.

Property	MT-A	MT-C
**Molecular weight (Da)**	581.27	599.26
**Consensus LogP**	8.78	9.14
**TPSA (Å^2^)**	46.53	46.53
**Water solubility**	Poorly soluble	Poorly soluble
**GI absorption**	Low	Low
**Lipinski violations**	2	2
**Veber rule**	Yes	Yes
**PAINS alerts**	0	0
**Brenk alerts**	1	1
**Bioavailability score**	0.17	0.17

## Data Availability

The data presented in this study are available within the article and the Supplementary Materials.

## Supplementary Materials

The following supporting information can be downloaded at: https://www.mdpi.com/article/10.3390/life16081335/s1, Figures S1–S60: ^1^H NMR, ^13^C NMR, and HRMS spectra of all synthesized compounds.

## References

[1] Liu Y., Yang Z., Zhang Q., Hai P., Zheng Y., Zhang J., Pan X. (2025). Recent Advances in Signaling Pathways and Kinase Inhibitors for Leukemia Chemotherapy. Curr. Med. Chem..

[2] Ponnusamy U., Perumal V. (2026). Comprehensive review on learning models of leukemia detection based on morphological information. Leuk. Lymphoma.

[3] Pechlivani L., Giannakis A., Sioka C., Alexiou G.A., Kyritsis A.P. (2025). Natural Products Targeting BCR-ABL: A Plant-Based Approach to Chronic Myeloid Leukemia Treatment. Molecules.

[4] Wang C.G., Zhong L., Liu Y.L., Shi X.J., Shi L.Q., Zeng L., Liu B.Z. (2017). Emodin Exerts an Antiapoptotic Effect on Human Chronic Myelocytic Leukemia K562 Cell Lines by Targeting the PTEN/PI3K-AKT Signaling Pathway and Deleting BCR-ABL. Integr. Cancer Ther..

[5] Wang X.Y., Sun G.B., Wang Y.J., Yan F. (2020). Emodin Inhibits Resistance to Imatinib by Downregulation of Bcr-Abl and STAT5 and Allosteric Inhibition in Chronic Myeloid Leukemia Cells. Biol. Pharm. Bull..

[6] Huang H., Weng H., Dong B., Zhao P., Zhou H., Qu L. (2017). Oridonin Triggers Chaperon-mediated Proteasomal Degradation of BCR-ABL in Leukemia. Sci. Rep..

[7] Elf S.E. (2020). “All Our Wisdom is Stored in the Trees”—Degrading BCR-ABL with Berberis Vulgaris. Clin. Cancer Res..

[8] Bahari H., Salim Z., Kardanpour L., Kouchaki H., Shoja F., Rahnama I., Faridzadeh A., Zahedani M.R. (2025). The Anti-Leukemic Potential of Curcumin in Chronic Myeloid Leukemia: A Systematic Review of In Vitro Studies. Food Sci. Nutr..

[9] Chakraborty P.K., Mustafi S.B., Ganguly S., Chatterjee M., Raha S. (2008). Resveratrol induces apoptosis in K562 (chronic myelogenous leukemia) cells by targeting a key survival protein, heat shock protein 70. Cancer Sci..

[10] Nistor M., Rugina D., Diaconeasa Z., Socaciu C., Socaciu M.A. (2023). Pentacyclic Triterpenoid Phytochemicals with Anticancer Activity: Updated Studies on Mechanisms and Targeted Delivery. Int. J. Mol. Sci..

[11] Barreto Vianna D.R., Gotardi J., Baggio Gnoatto S.C., Pilger D.A. (2021). Natural and Semisynthetic Pentacyclic Triterpenes for Chronic Myeloid Leukemia Therapy: Reality, Challenges and Perspectives. ChemMedChem.

[12] Teng W., Zhou Z., Cao J., Guo Q. (2024). Recent Advances of Natural Pentacyclic Triterpenoids as Bioactive Delivery System for Synergetic Biological Applications. Foods.

[13] Ciftci H.I., Radwan M.O., Ozturk S.E., Ulusoy N.G., Sozer E., Ellakwa D.E., Ocak Z., Can M., Ali T.F.S., Abd-Alla H.I. (2019). Design, Synthesis and Biological Evaluation of Pentacyclic Triterpene Derivatives: Optimization of Anti-ABL Kinase Activity. Molecules.

[14] Tang Z.Y., Li Y., Tang Y.T., Ma X.D., Tang Z.Y. (2022). Anticancer activity of oleanolic acid and its derivatives: Recent advances in evidence, target profiling and mechanisms of action. BioMed Pharmacother..

[15] Gao C.X., Tang C.H., Wu T.J., Hu Y., Peng Y.L., Liu M.L., Liu Q.W., Chen H.F., Yang Z.H., Zheng X. (2023). Anticancer activity of oleanolic acid and its derivatives modified at A-ring and C-28 position. J. Asian Nat. Prod. Res..

[16] Shanmugam M.K., Dai X., Kumar A.P., Tan B.K., Sethi G., Bishayee A. (2014). Oleanolic acid and its synthetic derivatives for the prevention and therapy of cancer: Preclinical and clinical evidence. Cancer Lett..

[17] Zhang P., Li H., Chen D., Ni J., Kang Y., Wang S. (2007). Oleanolic acid induces apoptosis in human leukemia cells through caspase activation and poly(ADP-ribose) polymerase cleavage. Acta Biochim Biophys. Sin..

[18] Pan S., Hu J., Zheng T., Liu X., Ju Y., Xu C. (2015). Oleanolic acid derivatives induce apoptosis in human leukemia K562 cell involved in inhibition of both Akt1 translocation and pAkt1 expression. Cytotechnology.

[19] Yang H., Deng M., Jia H., Zhang K., Liu Y., Cheng M., Xiao W. (2024). A review of structural modification and biological activities of oleanolic acid. Chin. J. Nat. Med..

[20] Jannus F., Sainz J., Reyes-Zurita F.J. (2024). Principal Bioactive Properties of Oleanolic Acid, Its Derivatives, and Analogues. Molecules.

[21] Wilcken R., Zimmermann M.O., Lange A., Joerger A.C., Boeckler F.M. (2013). Principles and Applications of Halogen Bonding in Medicinal Chemistry and Chemical Biology. J. Med. Chem..

[22] Lu Y., Liu Y., Xu Z., Li H., Liu H., Zhu W. (2012). Halogen bonding for rational drug design and new drug discovery. Expert Opin. Drug Discov..

[23] Chu F., Zhang W., Guo W., Wang Z., Yang Y., Zhang X., Fang K., Yan M., Wang P., Lei H. (2018). Oleanolic Acid-amino Acids Derivatives: Design, Synthesis, and Hepatoprotective Evaluation In Vitro and In Vivo. Molecules.

[24] Ciftci H.I., Bayrak N., Yıldız M., Yıldırım H., Sever B., Tateishi H., Otsuka M., Fujita M., Tuyun A.F. (2021). Design, synthesis and investigation of the mechanism of action underlying anti-leukemic effects of the quinolinequinones as LY83583 analogs. Bioorg Chem..

[25] Ulusoy N.G., Emirdağ S., Sözer E., Radwan M.O., Çiftçi H., Aksel M., Bölükbaşı S.Ş., Özmen A., Yaylı N., Karayıldırım T. (2022). Design, semi-synthesis and examination of new gypsogenin derivatives against leukemia via Abl tyrosine kinase inhibition and apoptosis induction. Int. J. Biol. Macromol..

[26] Sever B., Ciftci H. (2026). Design, Synthesis, and Biological Evaluation of N,N-Diphenylaniline-Based Derivatives as Antiproliferative Agents and ABL TK Inhibitors Against CML. Pharmaceuticals.

[27] Mosmann T. (1983). Rapid colorimetric assay for cellular growth and survival: Application to proliferation and cytotoxicity assays. J. Immunol. Methods.

[28] Wang B., Liu J., Gong Z. (2015). Resveratrol induces apoptosis in K562 cells via the regulation of mitochondrial signaling pathways. Int. J. Clin. Exp. Med..

[29] van Engeland M., Nieland L.J.W., Ramaekers F.C.S., Schutte B., Reutelingsperger C.P.M. (1998). Annexin V-affinity assay: A review on an apoptosis detection system based on phosphatidylserine exposure. Cytometry.

[30] Nagar B., Bornmann W.G., Pellicena P., Schindler T., Veach D.R., Miller W.T., Clarkson B., Kuriyan J. (2002). Crystal structures of the kinase domain of c-Abl in complex with the small molecule inhibitors PD173955 and imatinib (STI-571). Cancer Res..

[31] Yamaguchi H., Hendrickson W.A. (1996). Structural basis for activation of human lymphocyte kinase Lck upon tyrosine phosphorylation. Nature.

[32] Schrödinger, LLC (2025). Schrödinger Release 2025.

[33] Sever B., Türkeş C., Demir Y., Elamin K.M., Osman W., Oral K., Akıncı Genç S., Cantürk Z., Masunaga T., Kishimoto N. (2025). Hydrazonylthiazole Derivatives as Dual EGFR and ALR2 Inhibitors: Design, Synthesis, and Comprehensive In Vitro and In Silico Evaluation for Potential Anticancer Activity. Pharmaceuticals.

[34] Alzain A.A., Almogaddam M.A., Yousif R., Alqarni M.H., Foudah A.I., Osman W., Elamin K.M., Mohamed H.M., Moglad E., Ashour A. (2025). Molecular docking, molecular dynamics simulation, and pharmacophore-based virtual screening unveil natural compounds with TIM-3 inhibitory activity. J. Pharm. Bioallied Sci..

[35] SwissADME. http://www.swissadme.ch.

[36] Huang M., Lu J.J., Ding J. (2021). Natural Products in Cancer Therapy: Past, Present and Future. Nat. Prod. Bioprospect.

[37] Wang S.R., Xu T., Deng K., Wong C.W., Liu J., Fang W.S. (2017). Discovery of Farnesoid X Receptor Antagonists Based on a Library of Oleanolic Acid 3-O-Esters through Diverse Substituent Design and Molecular Docking Methods. Molecules.

[38] Ciftci H.I., Ozturk S.E., Ali T.F.S., Radwan M.O., Tateishi H., Koga R., Ocak Z., Can M., Otsuka M., Fujita M. (2018). The First Pentacyclic Triterpenoid Gypsogenin Derivative Exhibiting Anti-ABL1 Kinase and Anti-chronic Myelogenous Leukemia Activities. Biol. Pharm. Bull..

[39] Triaa N., Znati M., Ben Jannet H., Bouajila J. (2024). Biological Activities of Novel Oleanolic Acid Derivatives from Bioconversion and Semi-Synthesis. Molecules.

[40] Zeng L.R., Pan B.W., Cai J., Liu L.J., Dong Z.C., Zhou Y., Feng T.T., Shi Y. (2024). Construction, structural modification, and bioactivity evaluation of pentacyclic triterpenoid privileged scaffolds in active natural products. RSC Adv..

[41] Günther A., Zalewski P., Sip S., Ruszkowski P., Bednarczyk-Cwynar B. (2024). Oleanolic Acid Dimers with Potential Application in Medicine-Design, Synthesis, Physico-Chemical Characteristics, Cytotoxic and Antioxidant Activity. Int. J. Mol. Sci..

[42] Baer-Dubowska W., Narożna M., Krajka-Kuźniak V. (2021). Anti-Cancer Potential of Synthetic Oleanolic Acid Derivatives and Their Conjugates with NSAIDs. Molecules.

[43] Similie D., Minda D., Bora L., Kroškins V., Lugiņina J., Turks M., Dehelean C.A., Danciu C. (2024). An Update on Pentacyclic Triterpenoids Ursolic and Oleanolic Acids and Related Derivatives as Anticancer Candidates. Antioxidants.

[44] Mdleleni L., Rungqu P., Naki T. (2026). Recent Advances in the Development of Selected Triterpenoid-Based Hybrid Molecules and Their Antimicrobial Activities: A Review. Antibiotics.

[45] D’Mello R.S., Mendon V., Pai P., Das I., Sundara B.K. (2025). Exploring the therapeutic potential of oleanolic acid and its derivatives in cancer treatment: A comprehensive review. 3 Biotech.

[46] Hochhaus A., Larson R.A., Guilhot F., Radich J.P., Branford S., Hughes T.P., Baccarani M., Deininger M.W., Cervantes F., Fujihara S. (2017). IRIS Investigators, Long-Term Outcomes of Imatinib Treatment for Chronic Myeloid Leukemia. N. Engl. J. Med..

[47] Amarante-Mendes G.P., Rana A., Datoguia T.S., Hamerschlak N., Brumatti G. (2022). BCR-ABL1 Tyrosine Kinase Complex Signaling Transduction: Challenges to Overcome Resistance in Chronic Myeloid Leukemia. Pharmaceutics.

[48] Cheng F., Wang H., Li W., Zhang Y. (2024). Clinical pharmacokinetics and drug-drug interactions of tyrosine-kinase inhibitors in chronic myeloid leukemia: A clinical perspective. Crit. Rev. Oncol. Hematol..

[49] Roskoski R. (2022). Targeting BCR-Abl in the treatment of Philadelphia-chromosome positive chronic myelogenous leukemia. Pharmacol. Res..

[50] Putri A., Rinaldi I., Louisa M., Koesnoe S. (2019). The Role of STAT5 in Tyrosine Kinase Inhibitor (IMATINIB) Resistance in CML Patients. Acta Med. Indones..

[51] Jabbour E., Kantarjian H. (2020). Chronic myeloid leukemia: 2020 update on diagnosis, therapy and monitoring. Am. J. Hematol..

[52] Lee H., Basso I.N., Kim D.D.H. (2021). Target spectrum of the BCR-ABL tyrosine kinase inhibitors in chronic myeloid leukemia. Int. J. Hematol..

[53] Oruganti B., Lindahl E., Yang J., Amiri W., Rahimullah R., Friedman R. (2022). Allosteric enhancement of the BCR-Abl1 kinase inhibition activity of nilotinib by cobinding of asciminib. J. Biol. Chem..

[54] Bavaro L., Martelli M., Cavo M., Soverini S. (2019). Mechanisms of Disease Progression and Resistance to Tyrosine Kinase Inhibitor Therapy in Chronic Myeloid Leukemia: An Update. Int. J. Mol. Sci..

[55] Irgit A., Kamıs R., Sever B., Tuyun A.F., Otsuka M., Fujita M., Demirci H., Ciftci H. (2025). Structure and Dynamics of the ABL1 Tyrosine Kinase and Its Important Role in Chronic Myeloid Leukemia. Arch. Pharm..

[56] Andretta E., Costa C., Longobardi C., Damiano S., Giordano A., Pagnini F., Montagnaro S., Quintiliani M., Lauritano C., Ciarcia R. (2021). Potential Approaches Versus Approved or Developing Chronic Myeloid Leukemia Therapy. Front Oncol..

[57] Yurttaş N.Ö., Eşkazan A.E. (2020). Novel therapeutic approaches in chronic myeloid leukemia. Leuk. Res..

[58] Karatekin Arik İ., Gümüş N., Alcitepe İ., Gündüz C., Sever B., Ciftci H.İ., Saydam G., Tezcanli Kaymaz B. (2025). Targeting leukemic stem cells: Enhanced eradication via tivantinib (ARQ197) and asciminib (ABL001) with molecular docking-guided screening of therapeutic derivatives. J. Chemother..

[59] Brauer N.R., Kempen A.L., Hernandez D., Sintim H.O. (2024). Non-kinase off-target inhibitory activities of clinically-relevant kinase inhibitors. Eur. J. Med. Chem..

[60] Palacios E.H., Weiss A. (2004). Function of the Src-family kinases, Lck and Fyn, in T-cell development and activation. Oncogene.

[61] Hermiston M.L., Xu Z., Majeti R., Weiss A. (2002). Reciprocal regulation of lymphocyte activation by tyrosine kinases and phosphatases. J. Clin. Invest..

[62] Bommhardt U., Schraven B., Simeoni L. (2019). Beyond TCR Signaling: Emerging Functions of Lck in Cancer and Immunotherapy. Int. J. Mol. Sci..

