# Supplementary material for: Design, Synthesis and Biological Evaluation of Novel Oleanolic Acid Derivatives as Potential Anti-Leukemic Agents
Source: Life (Basel). 2026 Aug 14;16(8):1335. doi: 10.3390/life16081335 (PMC13514604; doi:10.3390/life16081335)

Supplementary Information

Design, Synthesis and Biological Evaluation of Novel Oleanolic Acid Derivatives as Potential Anti-Leukemic Agents

Nefise Demir [1], Halilibrahim Ciftci [2,3,4], Khaled M. Elamin [5], Mustafa Can [6,*] and Belgin Sever [2,7,*]

[1] Department of Nanoscience and Nanotechnology, Izmir Kâtip Celebi University, Izmir 35620, Türkiye; demir.nefise53@gmail.com
[2] Department of Molecular Biology and Genetics, Burdur Mehmet Akif Ersoy University, Istiklal Campus, Burdur 15200, Türkiye; hciftci@mehmetakif.edu.tr; belginsever@anadolu.edu.tr
[3] Medicinal and Biological Chemistry Science Farm Joint Research Laboratory, Faculty of Life Sciences, Kumamoto University, Kumamoto 862-0973, Japan
[4] Department of Drug Discovery, Science Farm Ltd., Kumamoto 862-0976, Japan
[5] Global Center for Natural Products Research, Faculty of Life Sciences, Kumamoto University, 5-1 Oe-honmachi, Chuo-ku, Kumamoto 862-0973, Japan; khaled@kumamoto-u.ac.jp
[6] Department of Bioengineering Sciences, Faculty of Engineering and Architecture, Izmir Kâtip Celebi University, Izmir 35620, Türkiye; mustafa.can@ikc.edu.tr
[7] Department of Pharmaceutical Chemistry, Faculty of Pharmacy, Anadolu University, Eskisehir 26470, Türkiye
* Correspondence: mustafa.can@ikc.edu.tr; belginsever@anadolu.edu.tr

Supplementary Figures

Figure S1: ^{1}H NMR Spectrum of **MT-A**
Figure S2: ^{13}C NMR Spectrum of **MT-A**
Figure S3: Mass Spectrum of **MT-A**
Figure S4: ^{1}H NMR Spectrum of **MT-B**
Figure S5: ^{13}C NMR Spectrum of **MT-B**
Figure S6: Mass Spectrum of **MT-B**
Figure S7: ^{1}H NMR Spectrum of **MT-C**
Figure S8: ^{13}C NMR Spectrum of **MT-C**
Figure S9: Mass Spectrum of **MT-C**
Figure S10: ^{1}H NMR Spectrum of **MT-D**
Figure S11: ^{13}C NMR Spectrum of **MT-D**
Figure S12: Mass Spectrum of **MT-D**
Figure S13: ^{1}H NMR Spectrum of **NOA-1**
Figure S14: ^{13}C NMR Spectrum of **NOA-1**
Figure S15: Mass Spectrum of **NOA-1**
Figure S16: ^{1}H NMR Spectrum of **NOA-2**
Figure S17: ^{13}C NMR Spectrum of **NOA-2**
Figure S18: Mass Spectrum of **NOA-2**
Figure S19: ^{1}H NMR Spectrum of **NOA-3**
Figure S20: ^{13}C NMR Spectrum of **NOA-3**
Figure S21: Mass Spectrum of **NOA-3**
Figure S22: ^{1}H NMR Spectrum of **NOA-4**
Figure S23: ^{13}C NMR Spectrum of **NOA-4**
Figure S24: Mass Spectrum of **NOA-4**
Figure S25: ^{1}H NMR Spectrum of **NOA-5**
Figure S26: ^{13}C NMR Spectrum of **NOA-5**
Figure S27: Mass Spectrum of **NOA-5**
Figure S28: ^{1}H NMR Spectrum of **NOA-6**
Figure S29: ^{13}C NMR Spectrum of **NOA-6**
Figure S30: Mass Spectrum of **NOA-6**
Figure S31: ^{1}H NMR Spectrum of **NOA-7**
Figure S32: ^{13}C NMR Spectrum of **NOA-7**
Figure S33: Mass Spectrum of **NOA-7**
Figure S34: ^{1}H NMR Spectrum of **NOA-8**
Figure S35: ^{13}C NMR Spectrum of **NOA-8**
Figure S36: Mass Spectrum of **NOA-8**
Figure S37: ^{1}H NMR Spectrum of **NOA-9**
Figure S38: ^{13}C NMR Spectrum of **NOA-9**
Figure S39: Mass Spectrum of **NOA-9**
Figure S40: ^{1}H NMR Spectrum of **NOA-10**
Figure S41: ^{13}C NMR Spectrum of **NOA-10**
Figure S42: Mass Spectrum of **NOA-10**
Figure S43: ^{1}H NMR Spectrum of **NOA-11**
Figure S44: ^{13}C NMR Spectrum of **NOA-11**
Figure S45: Mass Spectrum of **NOA-11**
Figure S46: ^{1}H NMR Spectrum of **NOA-12**

Figure S47: ^{13}C NMR Spectrum of **NOA-12**
Figure S48: Mass Spectrum of **NOA-12**
Figure S49: ^{1}H NMR Spectrum of **NOA-13**
Figure S50: ^{13}C NMR Spectrum of **NOA-13**
Figure S51: Mass Spectrum of **NOA-13**
Figure S52: ^{1}H NMR Spectrum of **NOA-14**
Figure S53: ^{13}C NMR Spectrum of **NOA-14**
Figure S54: Mass Spectrum of **NOA-14**
Figure S55: ^{1}H NMR Spectrum of **NOA-15**
Figure S56: ^{13}C NMR Spectrum of **NOA-15**
Figure S57: Mass Spectrum of **NOA-15**
Figure S58: ^{1}H NMR Spectrum of **NOA-16**
Figure S59: ^{13}C NMR Spectrum of **NOA-16**
Figure S60: Mass Spectrum of **NOA-16**

Figure S1: ^{1}H NMR Spectrum of **MT-A**

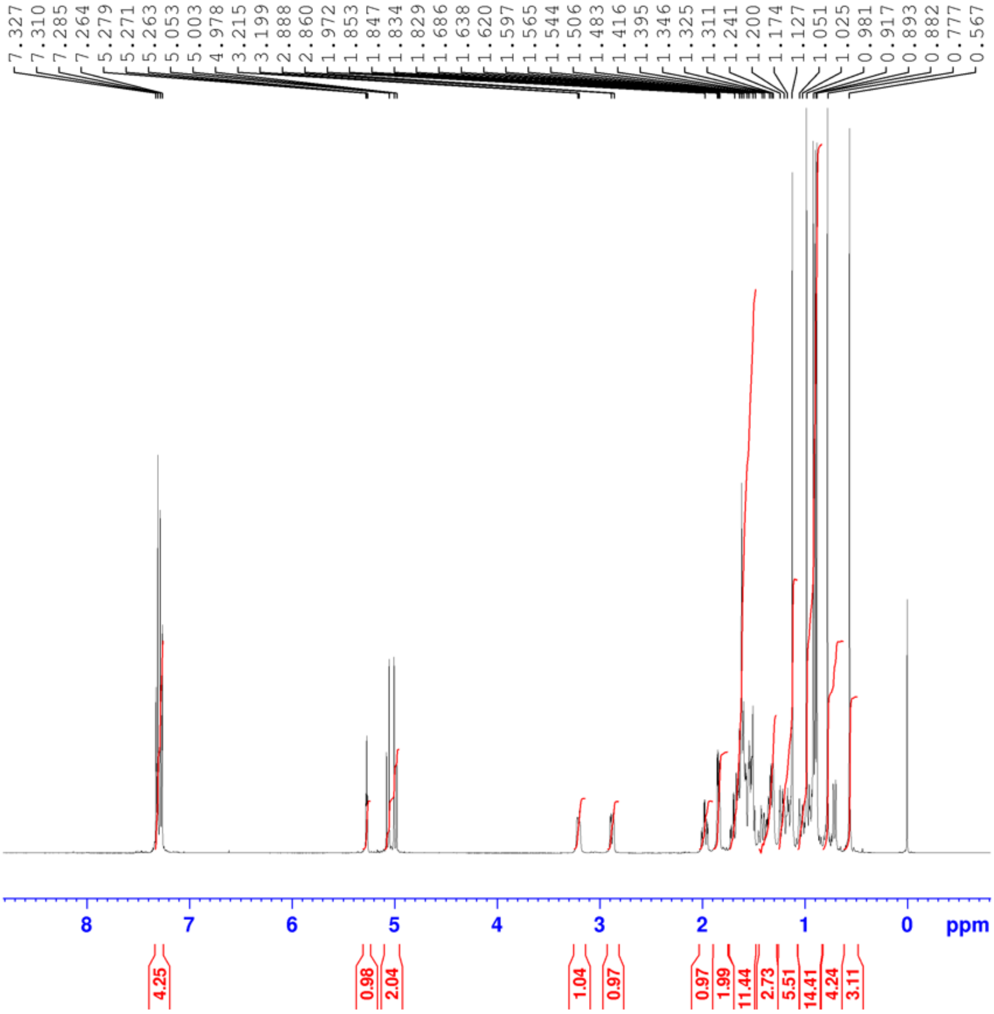

Figure S2: ^{13}C NMR Spectrum of **MT-A**

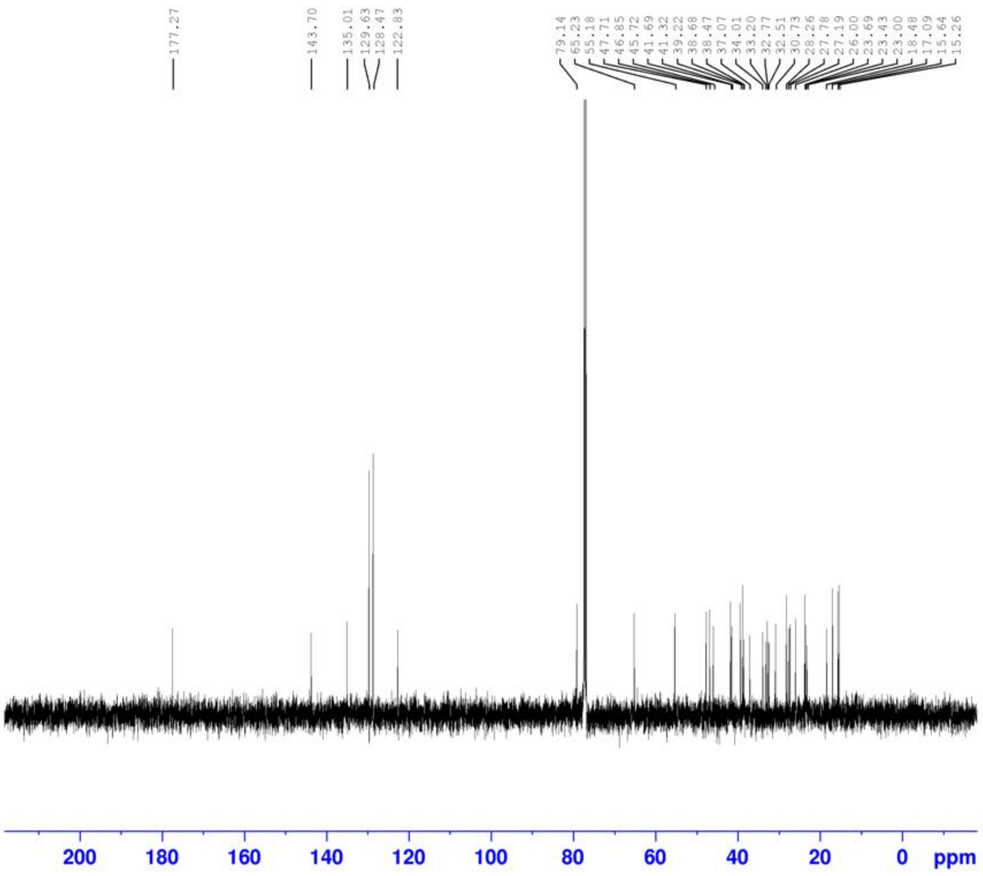

Figure S3: Mass Spectrum of **MT-A**

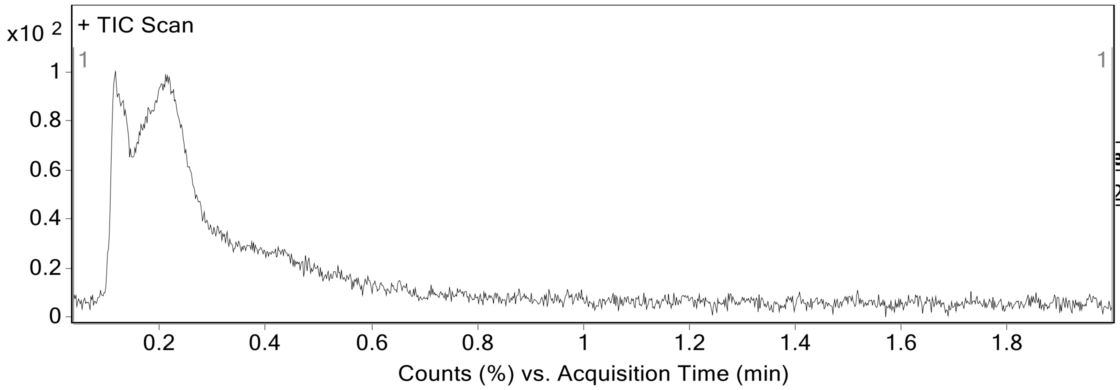

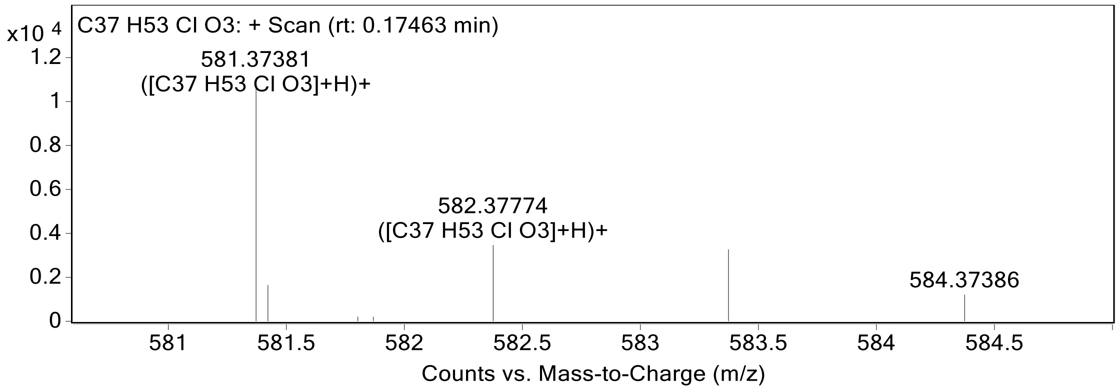

Formula Calculator Results

Formula	Best	Mass	Tgt Mass	Diff (ppm)	Ion Species	Score
C37 H53 Cl O3	TRUE	580,36686	580,36832	-2,52	C37 H54 Cl O3	77,24

Figure S4: ^{1}H NMR Spectrum of **MT-B**

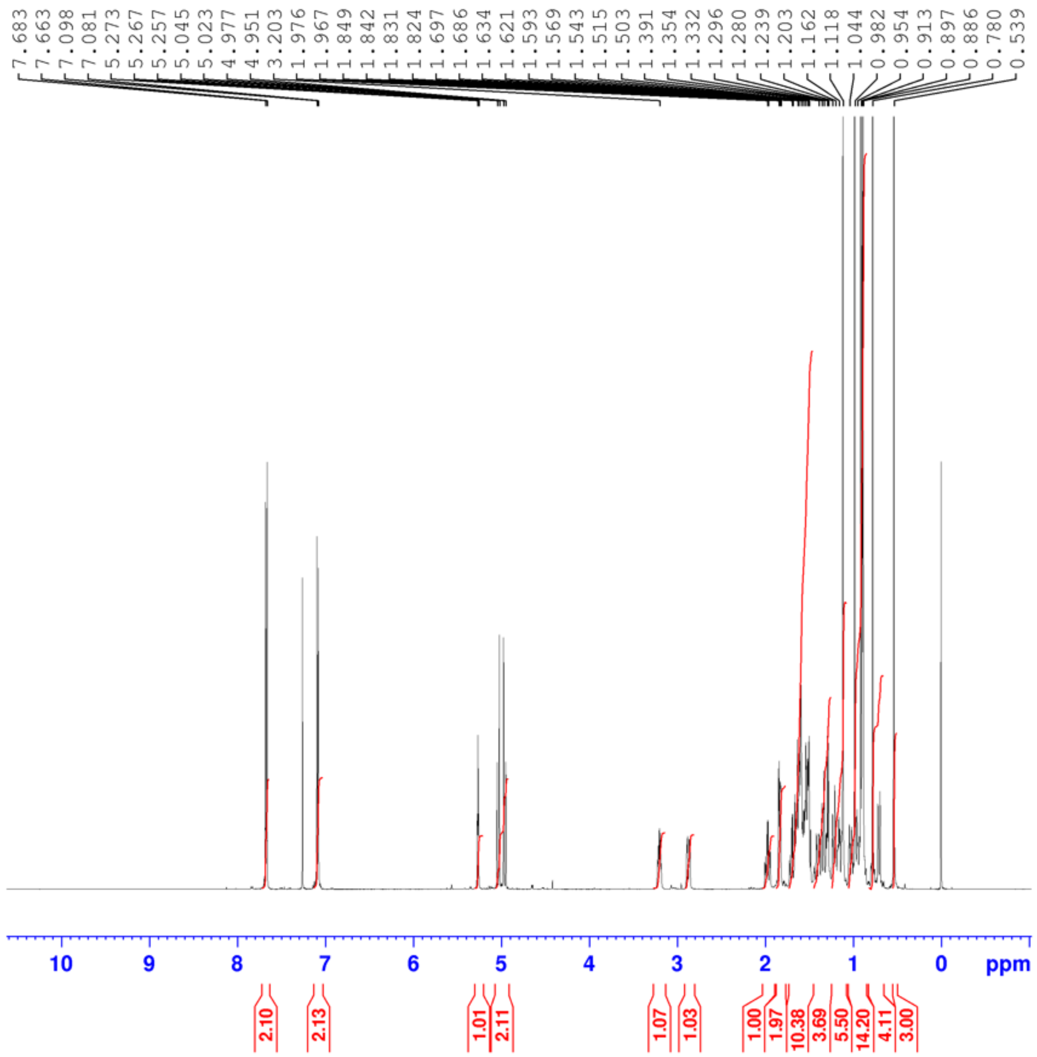

Figure S5: ^{13}C NMR Spectrum of **MT-B**

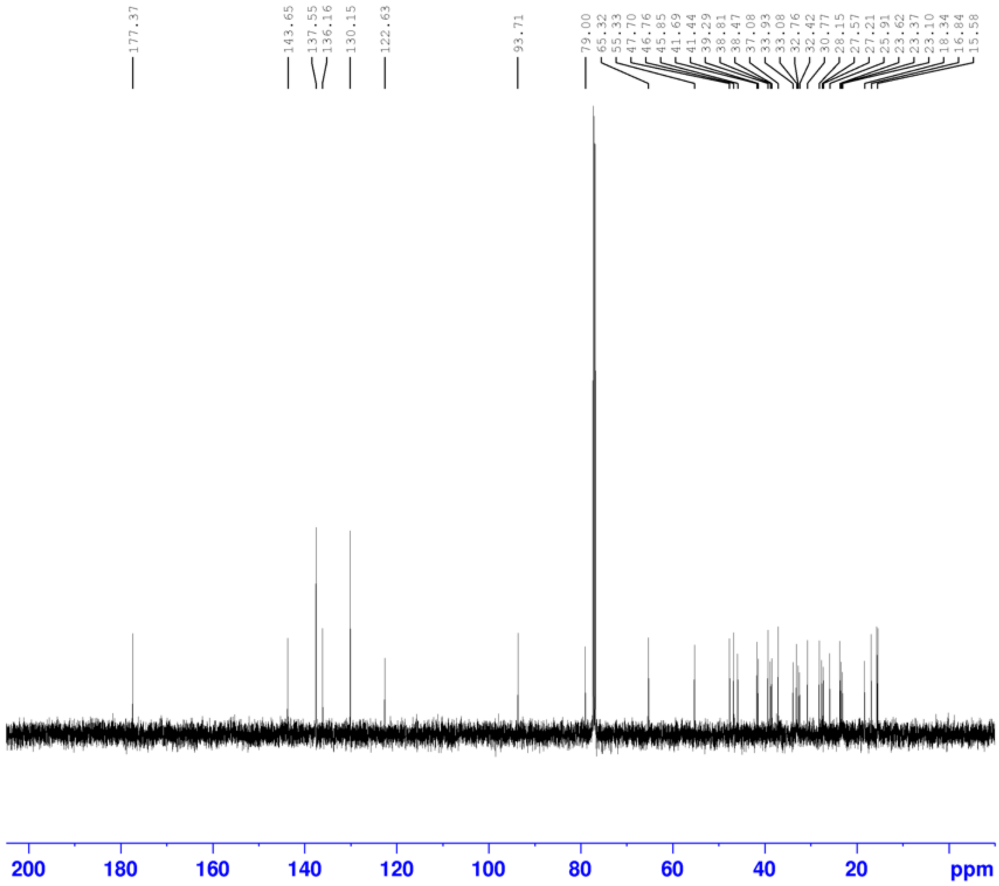

Figure S6: Mass Spectrum of **MT-B**

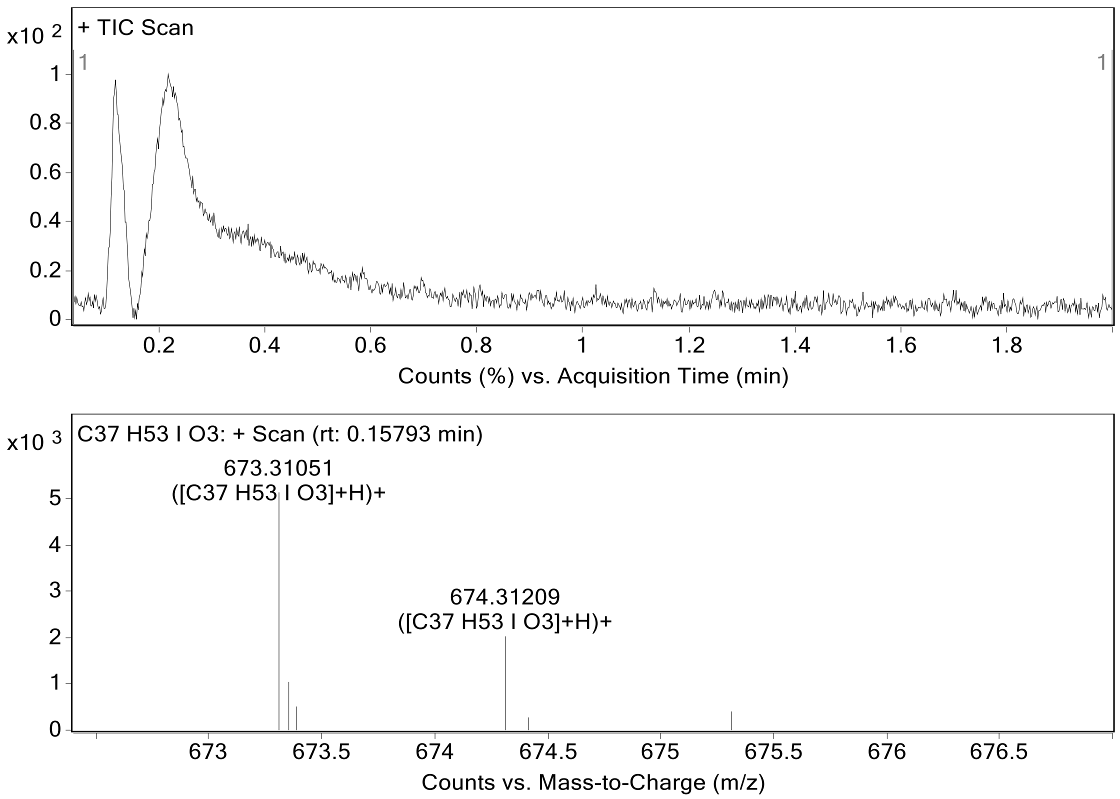

Formula Calculator Results

Formula	Best	Mass	Tgt Mass	Diff (ppm)	Ion Species	Score
C37 H53 I O3	TRUE	672,30256	672,30394	-2,05	C37 H54 I O3	93,31

Figure S7: ^{1}H NMR Spectrum of **MT-C**

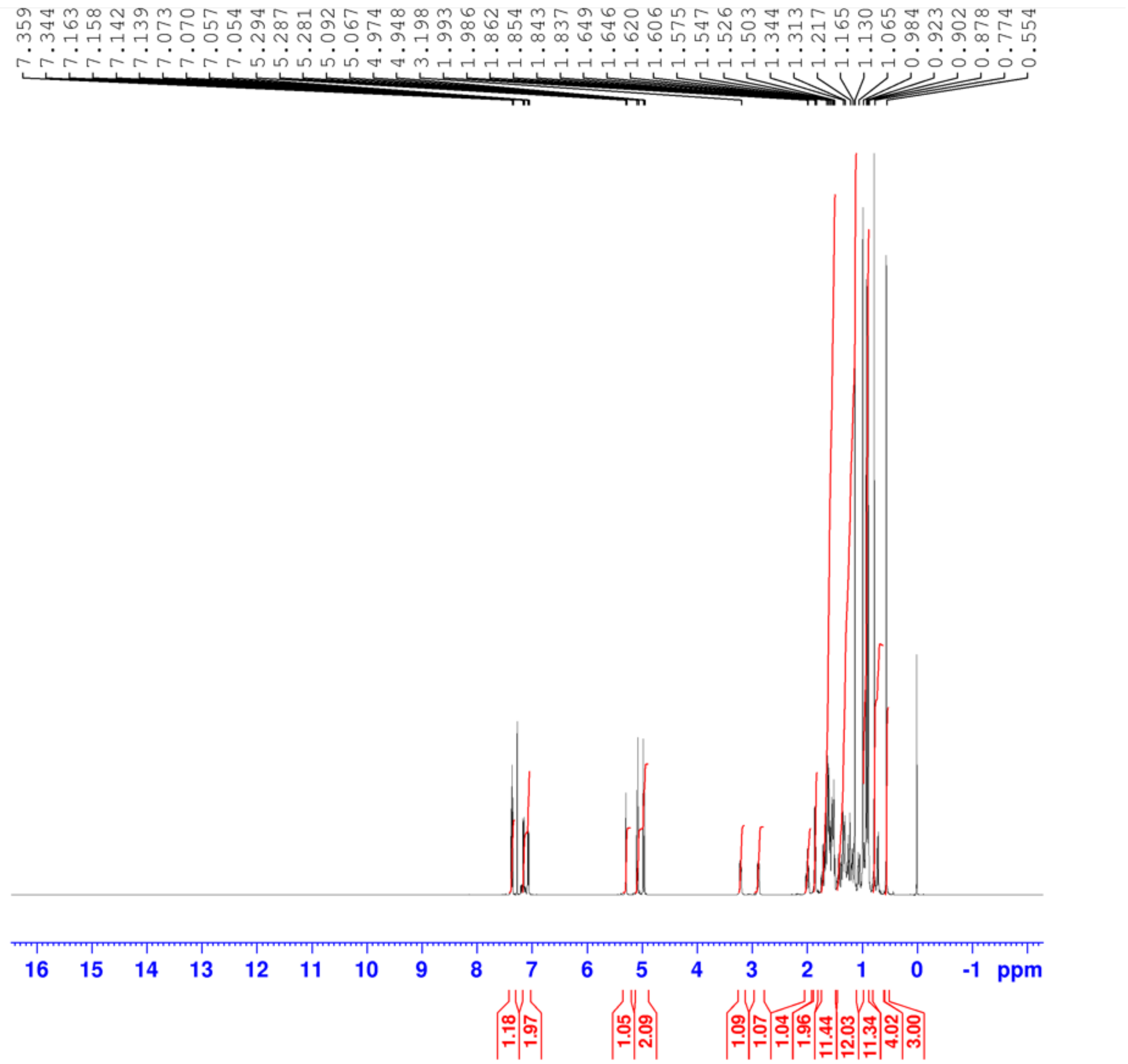

Figure S8: ^{13}C NMR Spectrum of **MT-C**

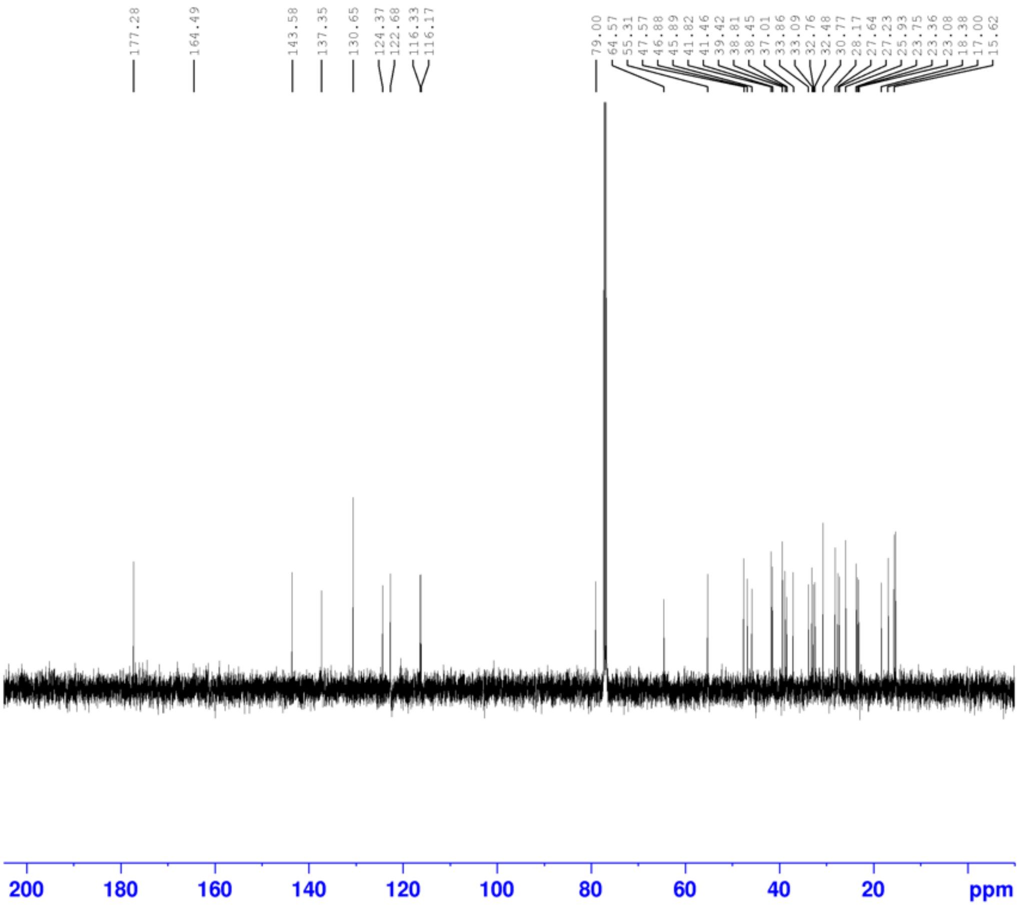

Figure S9: Mass Spectrum of **MT-C**

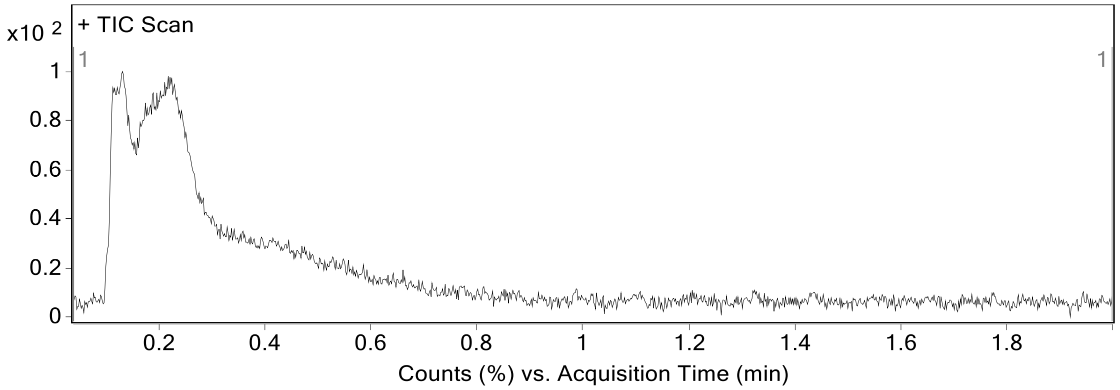

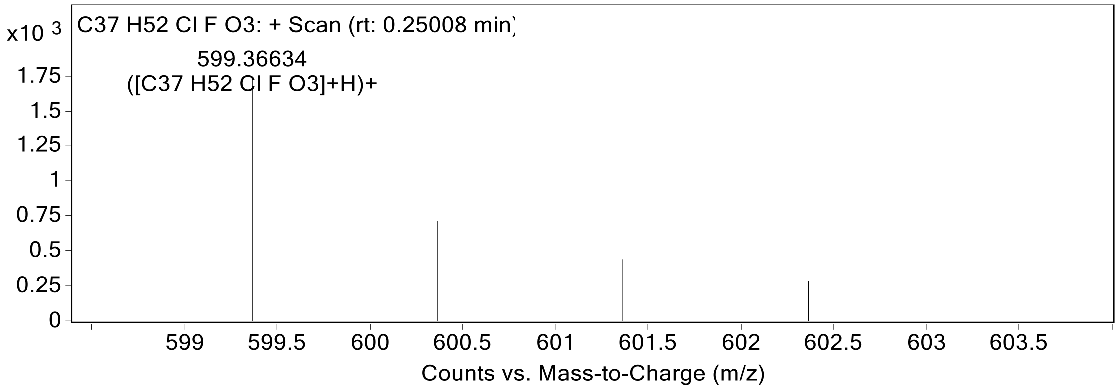

Formula Calculator Results

Formula	Best	Mass	Tgt Mass	Diff (ppm)	Ion Species	Score
C37 H52 Cl F O3	TRUE	598,35694	598,3589	-3,29	C37 H53 Cl F O3	58,46

Figure S10: ^{1}H NMR Spectrum of **MT-D**

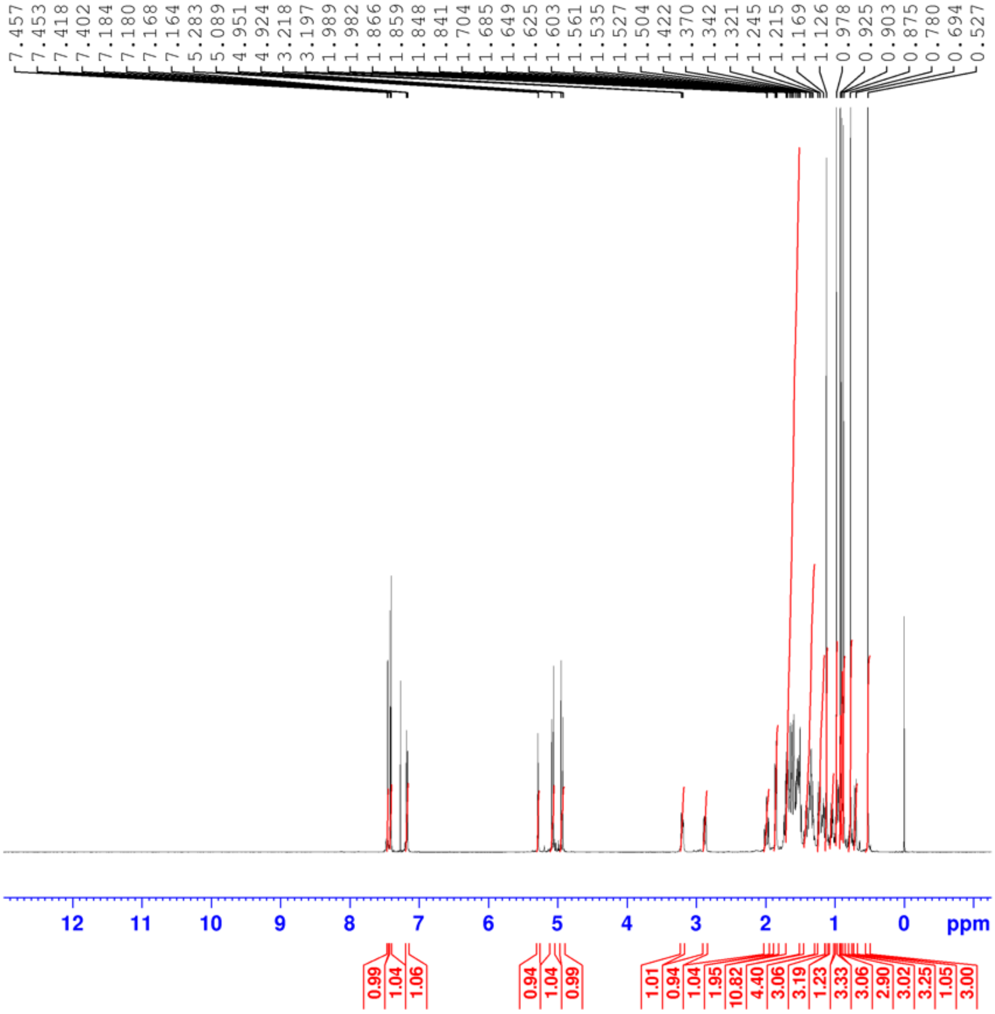

Figure S11: ^{13}C NMR Spectrum of **MT-D**

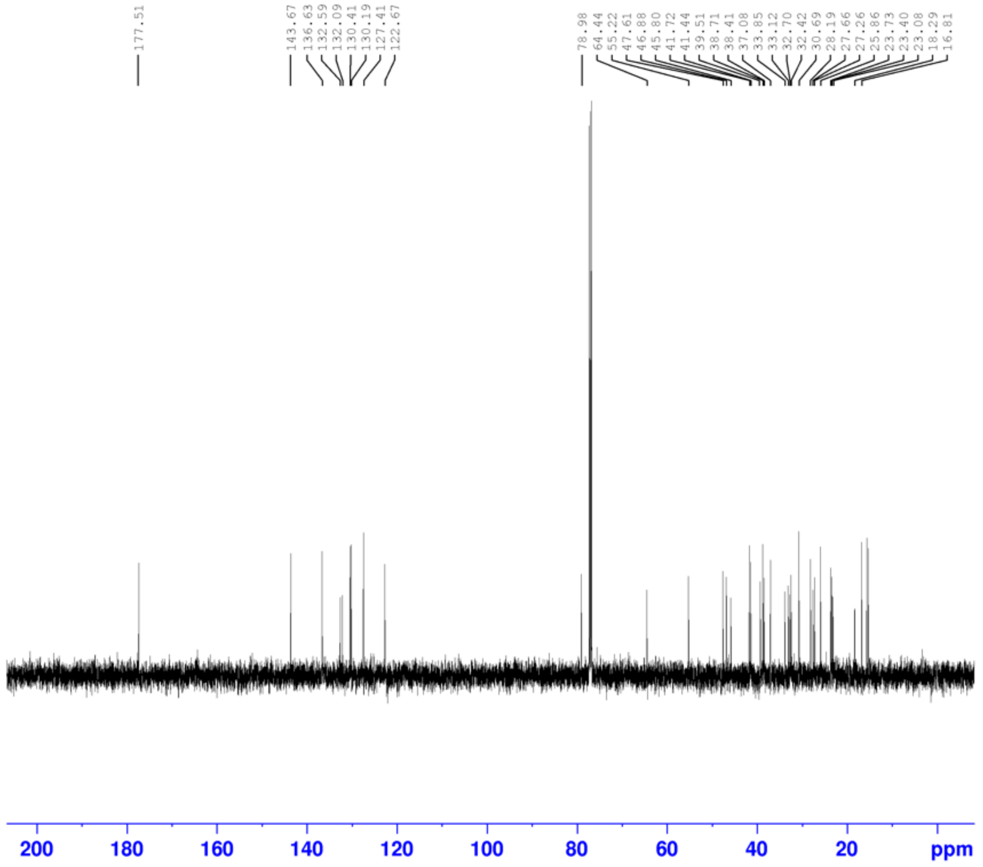

Figure S12: Mass Spectrum of **MT-D**

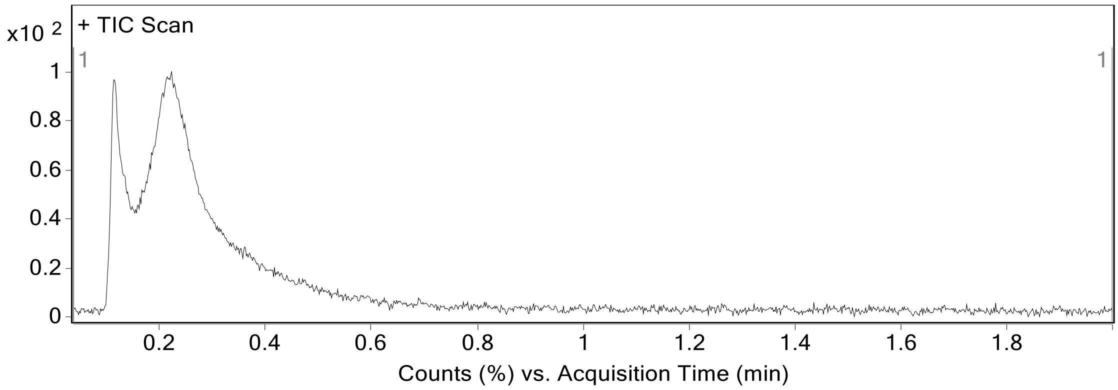

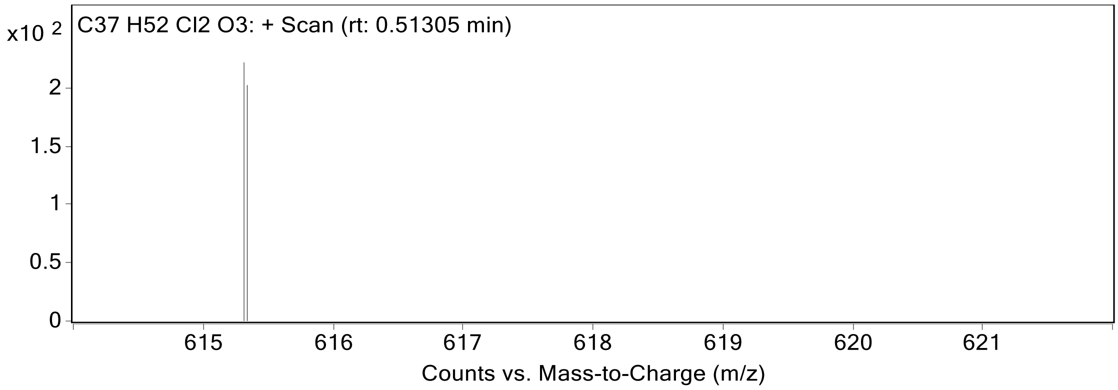

Formula Calculator Results

Formula	Best	Mass	Tgt Mass	Diff (ppm)	Ion Species	Score
C37 H52 Cl2 O3	TRUE	614,3317	614,32935	3,83	C37 H53 Cl2 O3	39,51

Figure S13: ^{1}H NMR Spectrum of **NOA-1**

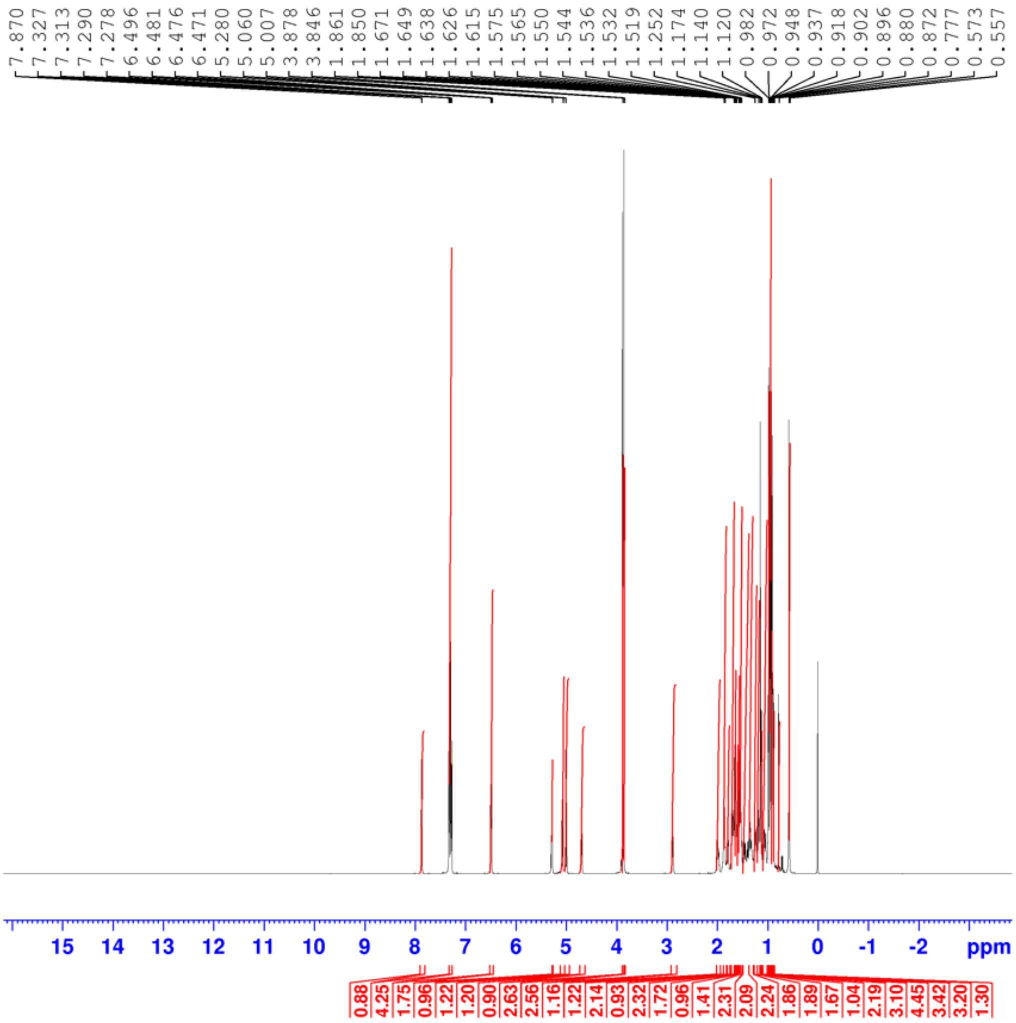

Figure S14: ^{13}C NMR Spectrum of **NOA-1**

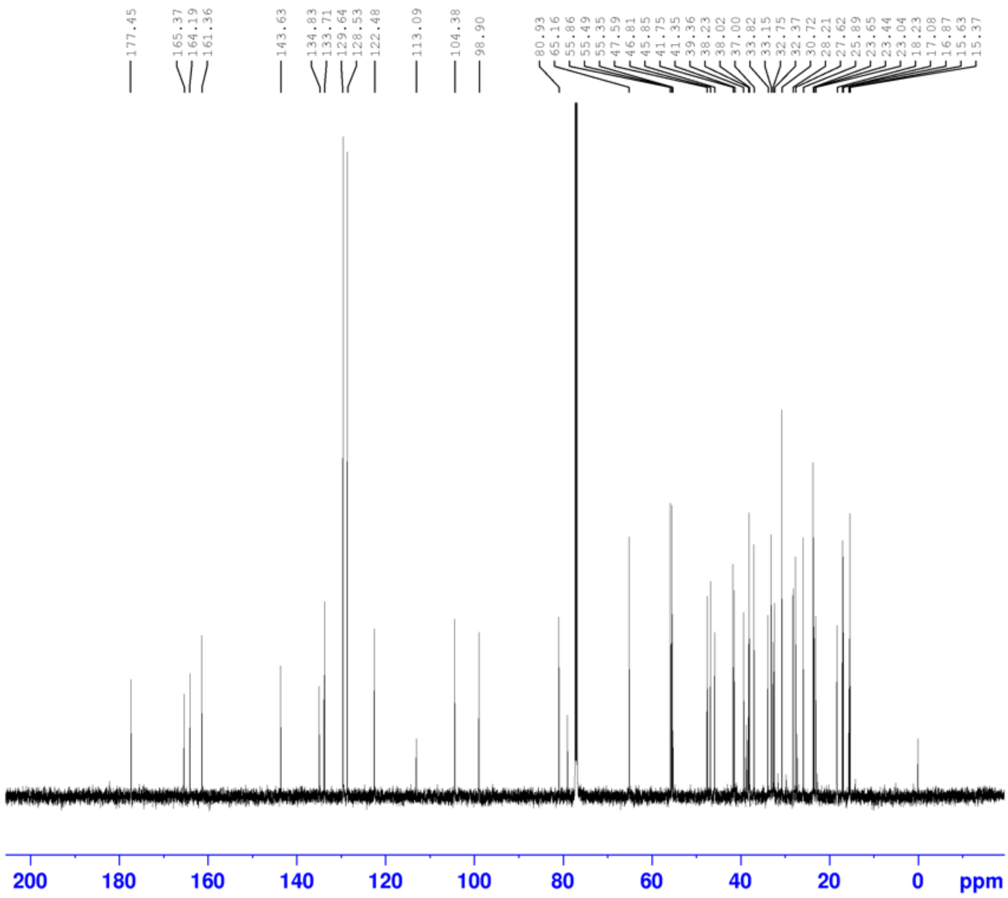

Figure S15: Mass Spectrum of **NOA-1**

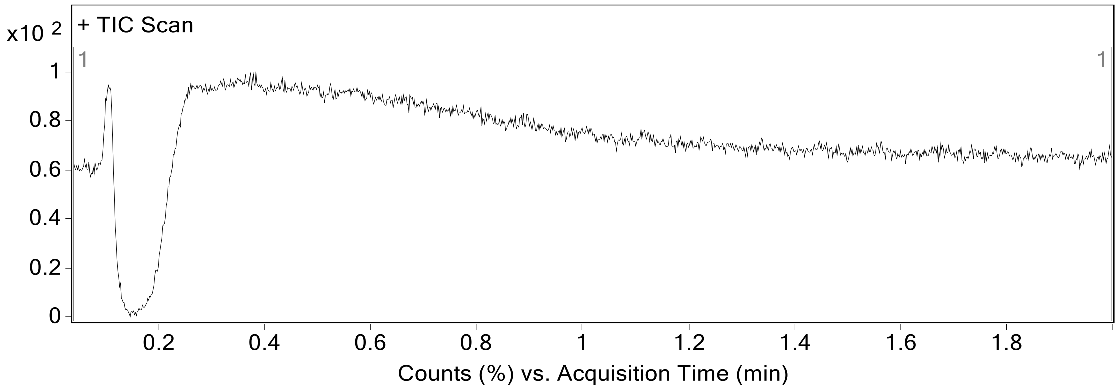

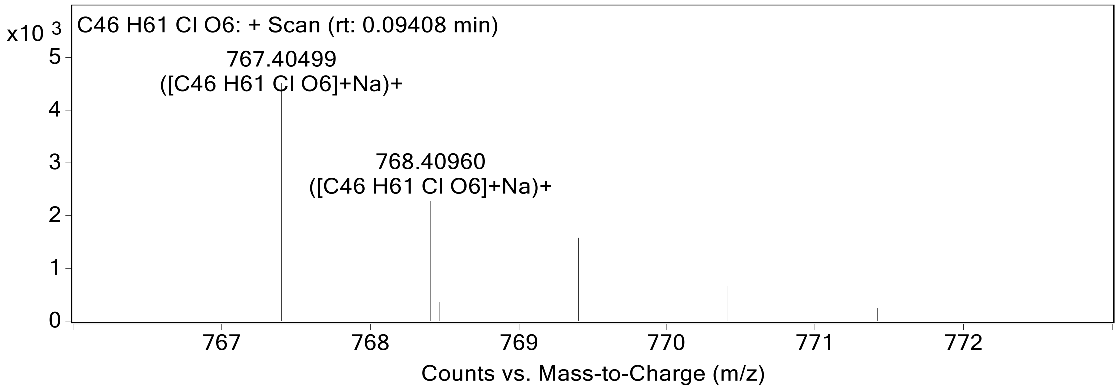

Formula Calculator Results

Formula	Best	Mass	Tgt Mass	Diff (ppm)	Ion Species	Score
C46 H61 Cl O6	TRUE	744,41594	744,41567	0,37	C46 H61 Cl Na O6	75,67

Figure S16: ^{1}H NMR Spectrum of **NOA-2**

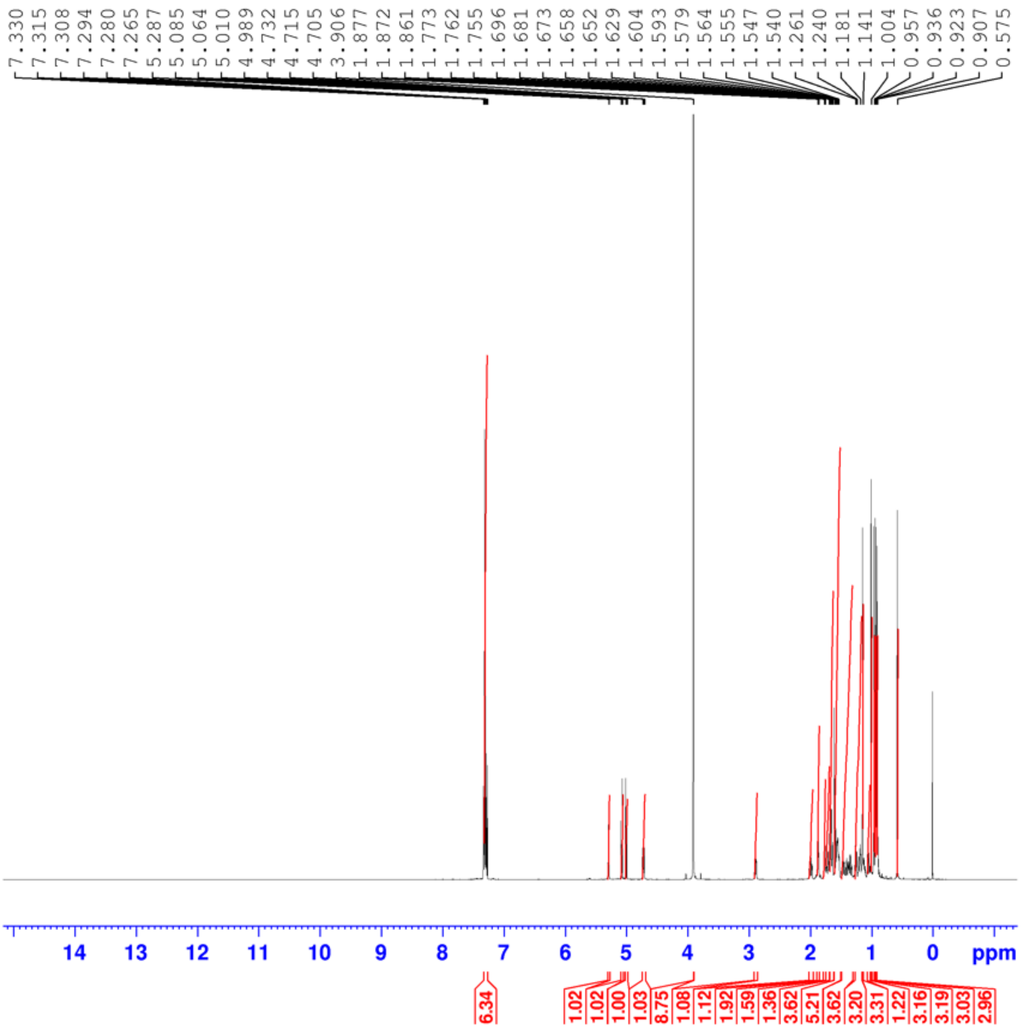

Figure S17: ^{13}C NMR Spectrum of **NOA-2**

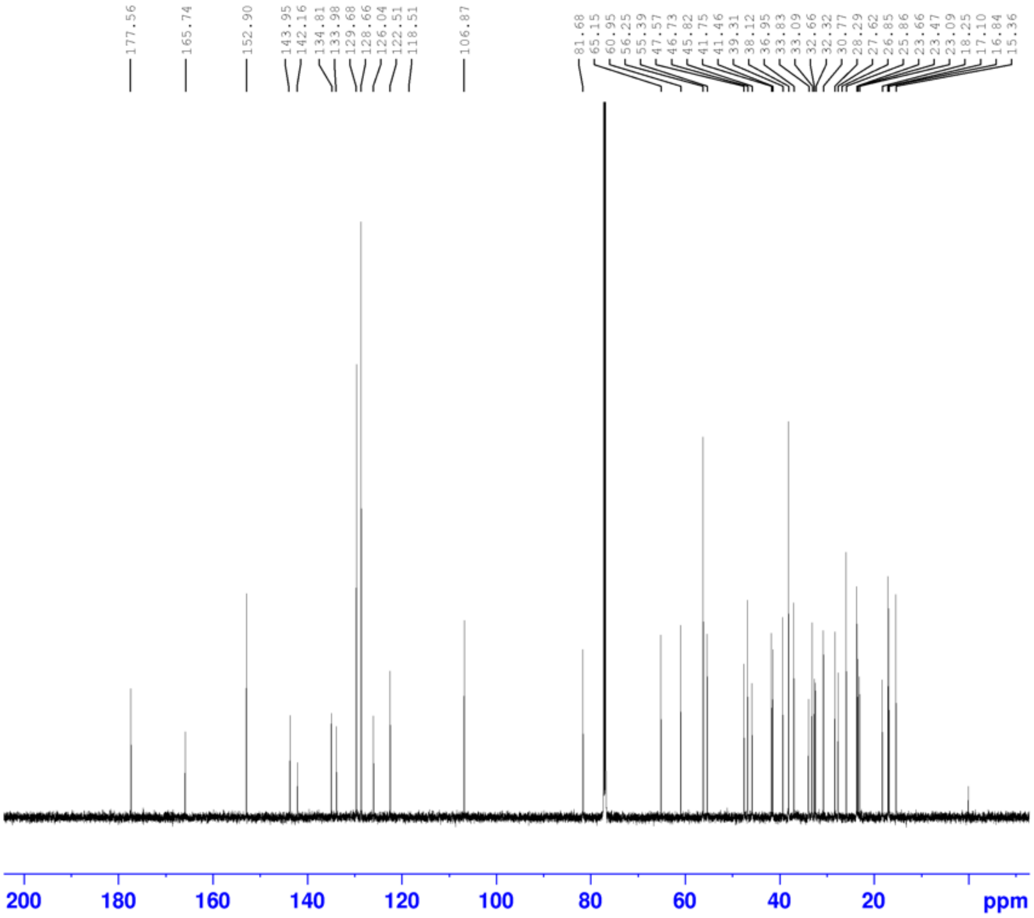

Figure S18: Mass Spectrum of **NOA-2**

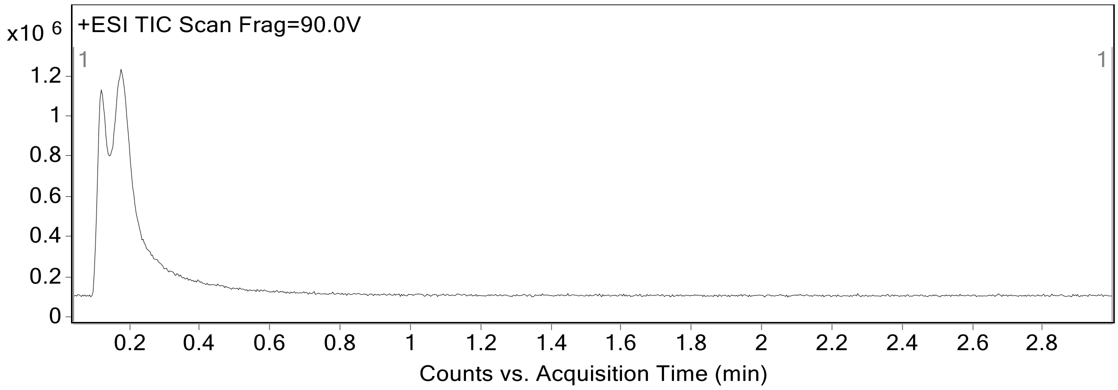

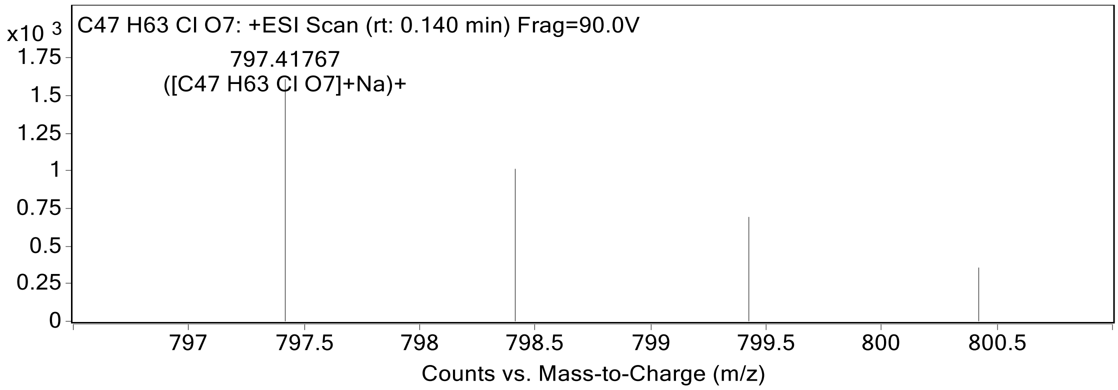

Formula Calculator Results

Formula	Best	Mass	Tgt Mass	Diff (ppm)	Ion Species	Score
C47 H63 Cl O7	TRUE	774,4278	774,42623	2,03	C47 H63 Cl Na O7	55,94

Figure S19: ^{1}H NMR Spectrum of **NOA-3**

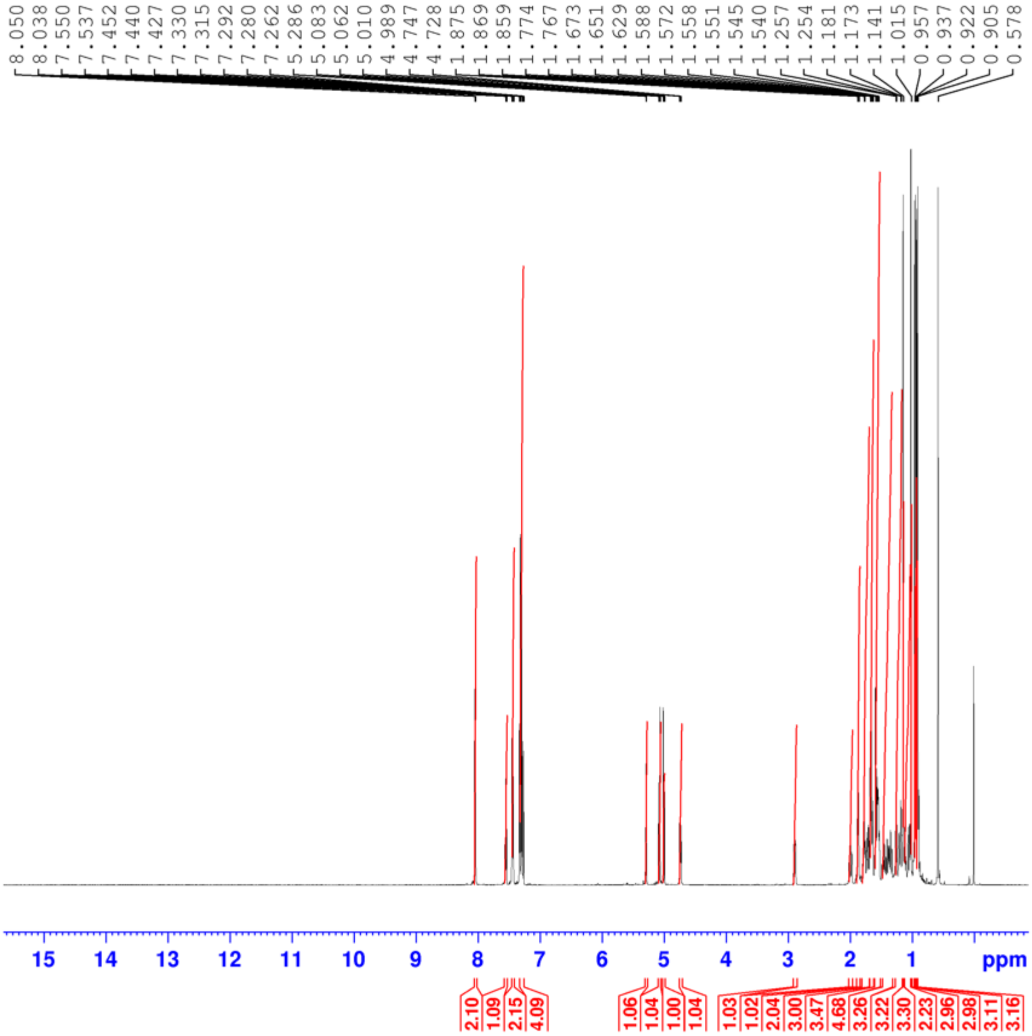

Figure S20: ^{13}C NMR Spectrum of **NOA-3**

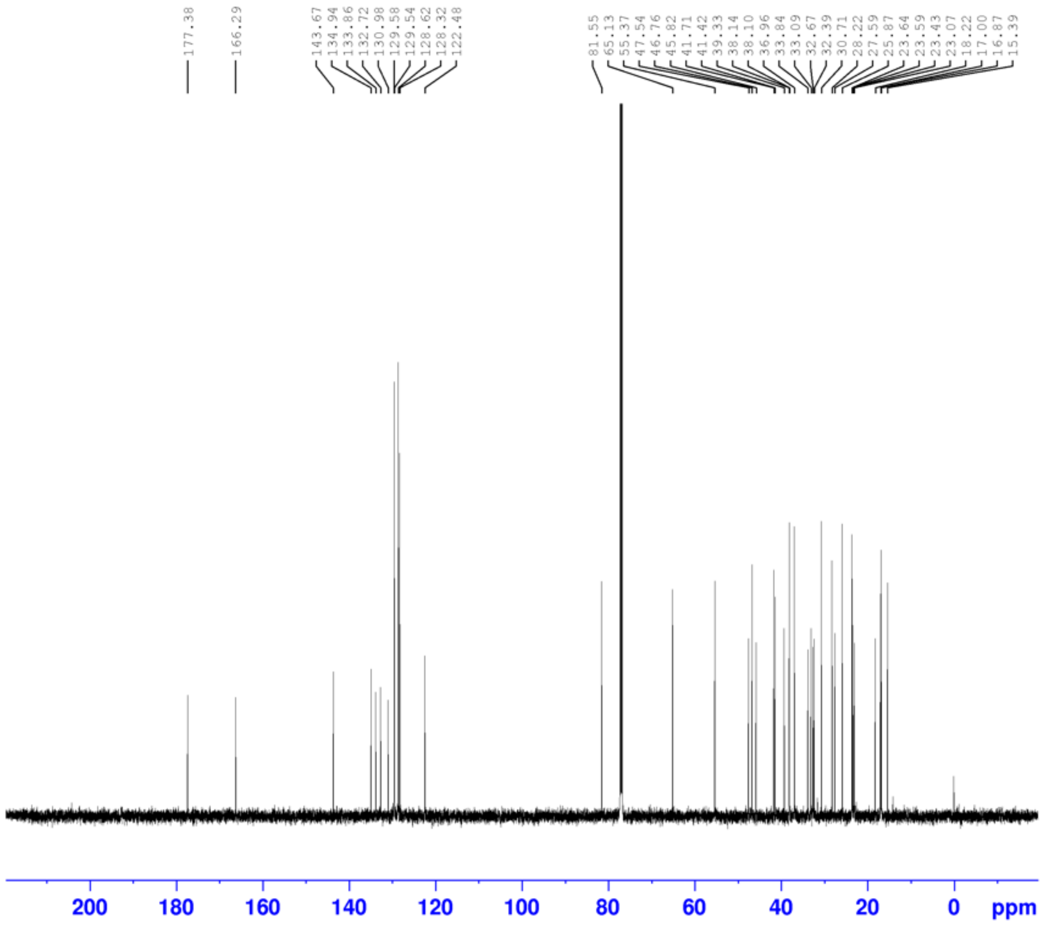

Figure S21: Mass Spectrum of **NOA-3**

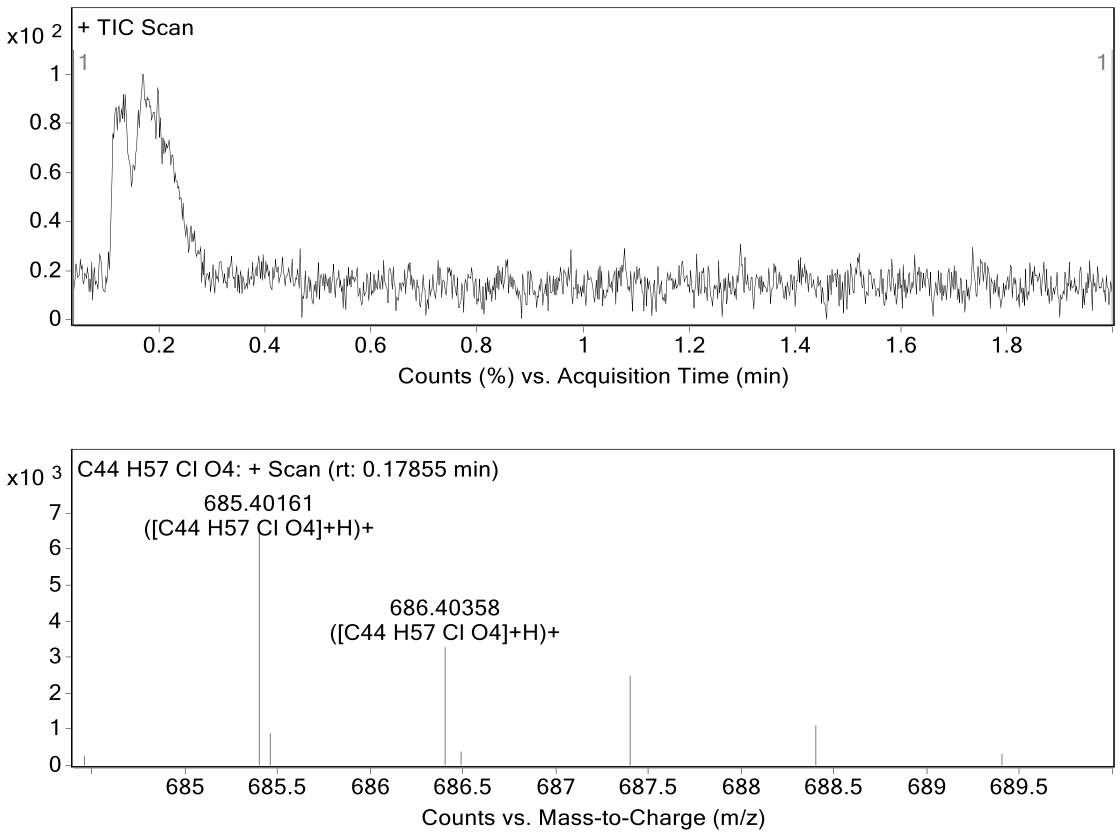

Formula Calculator Results

Formula	Best	Mass	Tgt Mass	Diff (ppm)	Ion Species	Score
C44 H57 Cl O4	TRUE	684,39379	684,39454	-1,09	C44 H58 Cl O4	75,85

Figure S22: ^{1}H NMR Spectrum of **NOA-4**

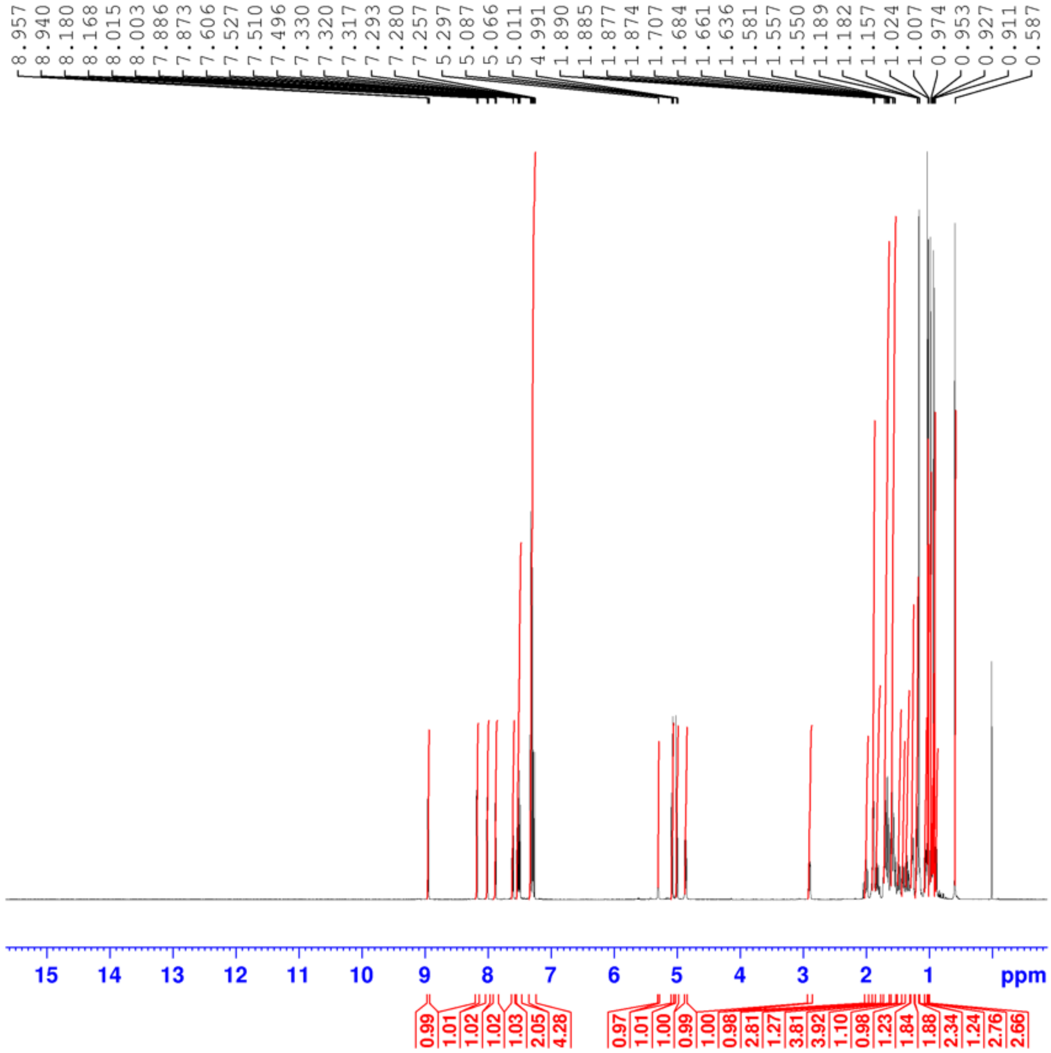

Figure S23: ^{13}C NMR Spectrum of **NOA-4**

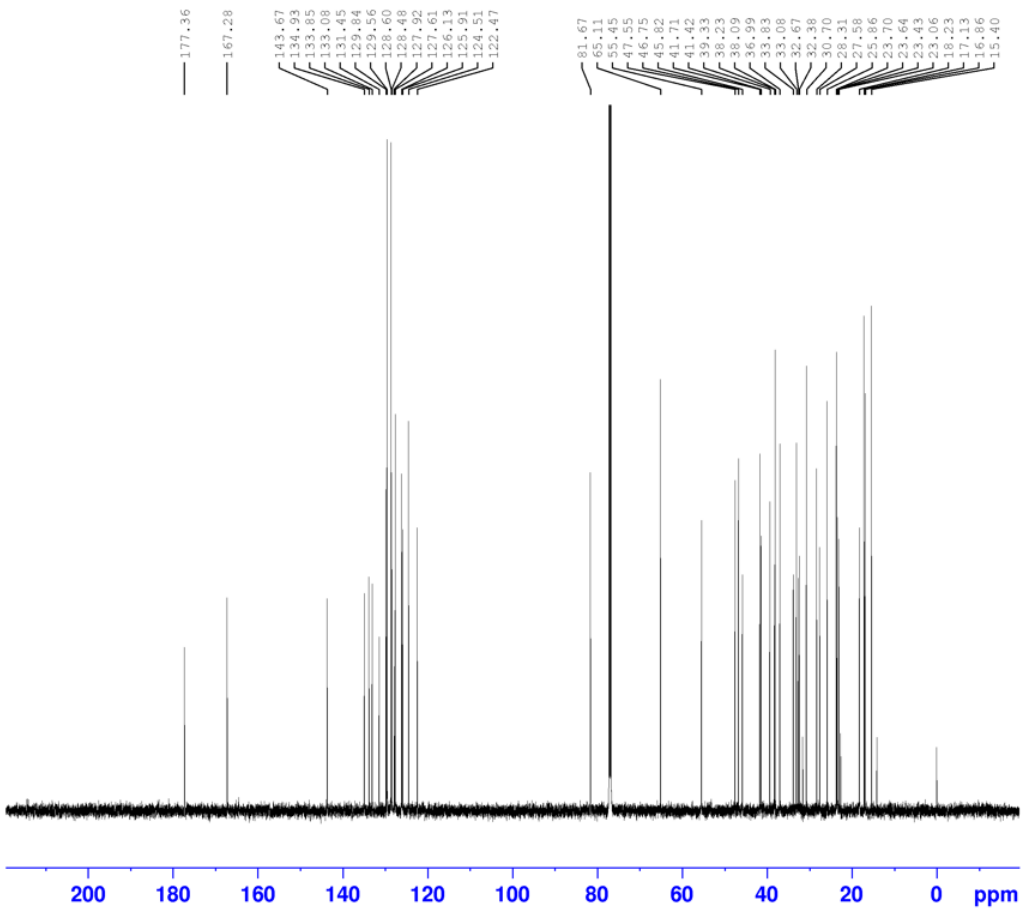

Figure S24: Mass Spectrum of **NOA-4**

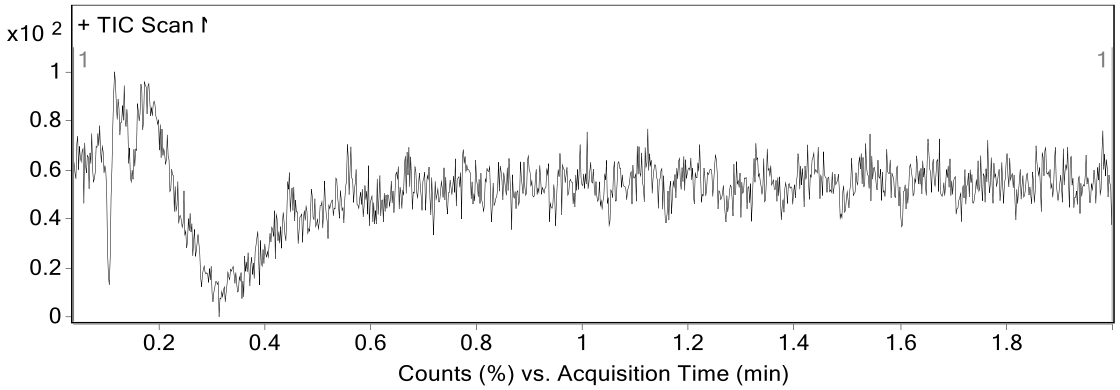

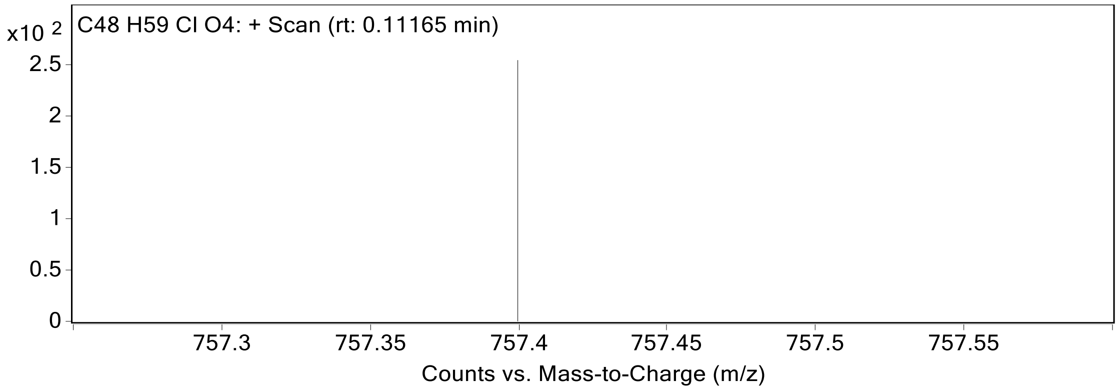

Formula Calculator Results

**Formula**	**Best**	**Mass**	**Tgt Mass**	**Diff (ppm)**	**Ion Species**	**Score**
C48 H59 Cl O4	TRUE	734,41048	734,41019	0,4	C48 H59 Cl Na O4	47,51

Figure S25: ^{1}H NMR Spectrum of **NOA-5**

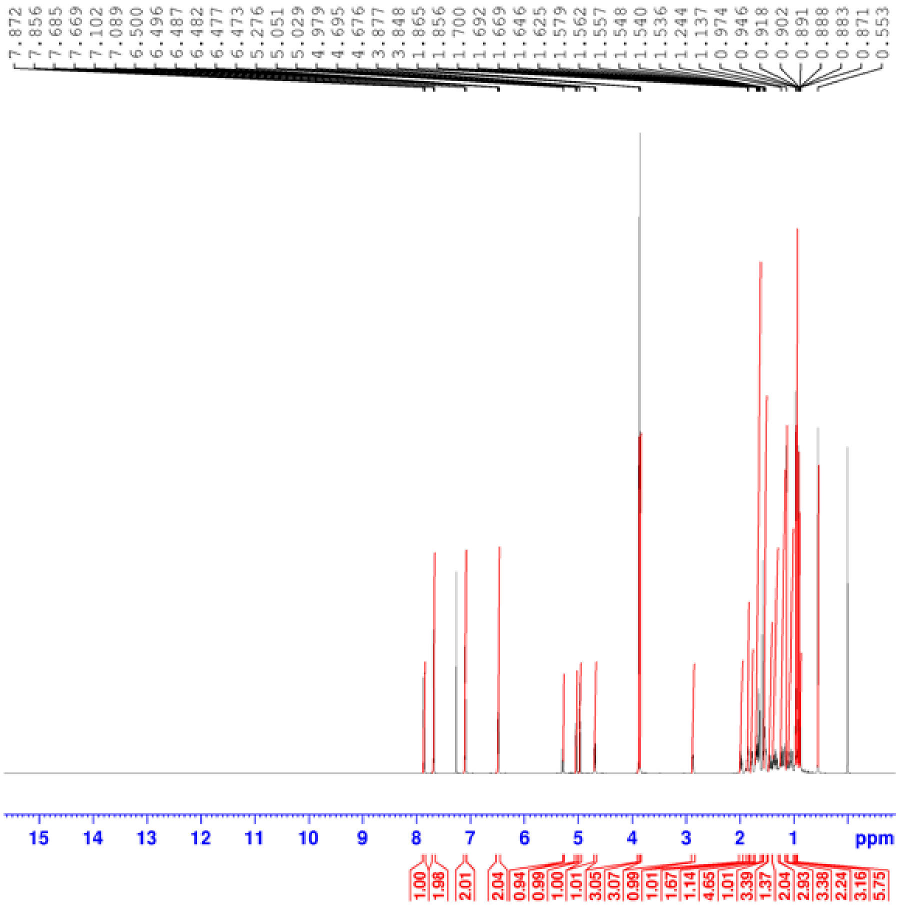

Figure S26: ^{13}C NMR Spectrum of **NOA-5**

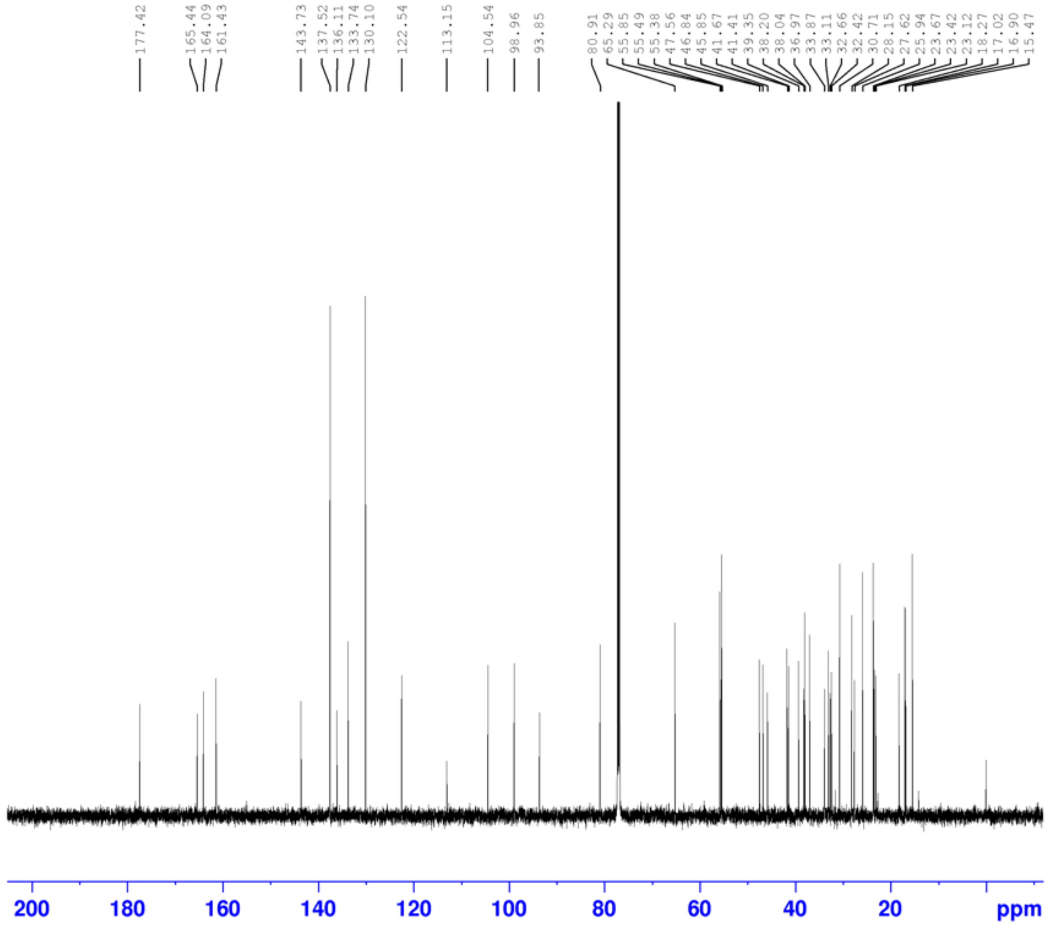

Figure S27: Mass Spectrum of **NOA-5**

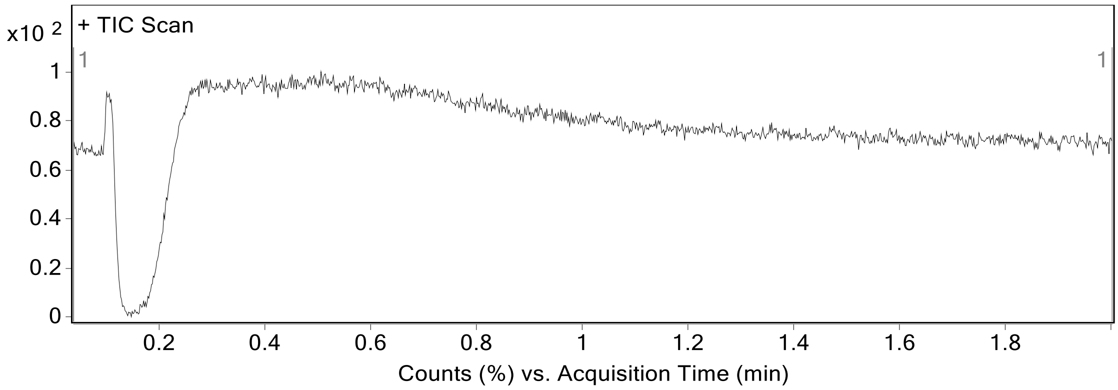

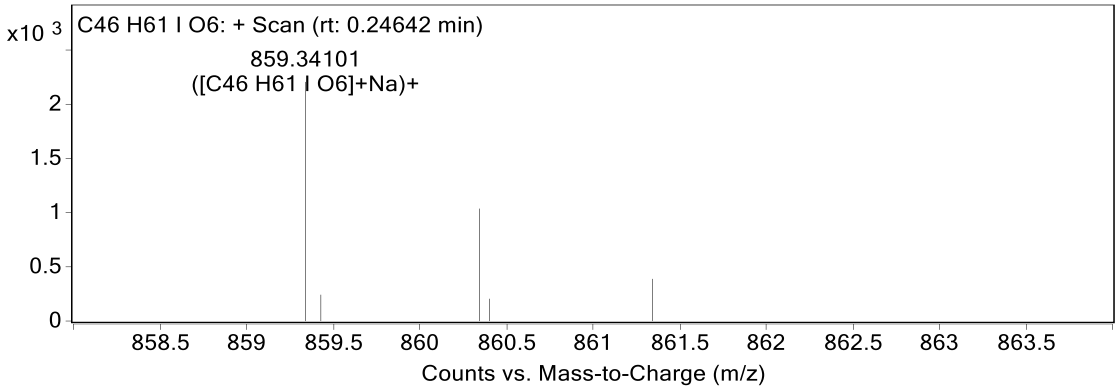

Formula Calculator Results

Formula	Best	Mass	Tgt Mass	Diff (ppm)	Ion Species	Score
C46 H61 I O6	TRUE	836,35143	836,35128	0,18	C46 H61 I Na O6	93,75

Figure S28: ^{1}H NMR Spectrum of **NOA-6**

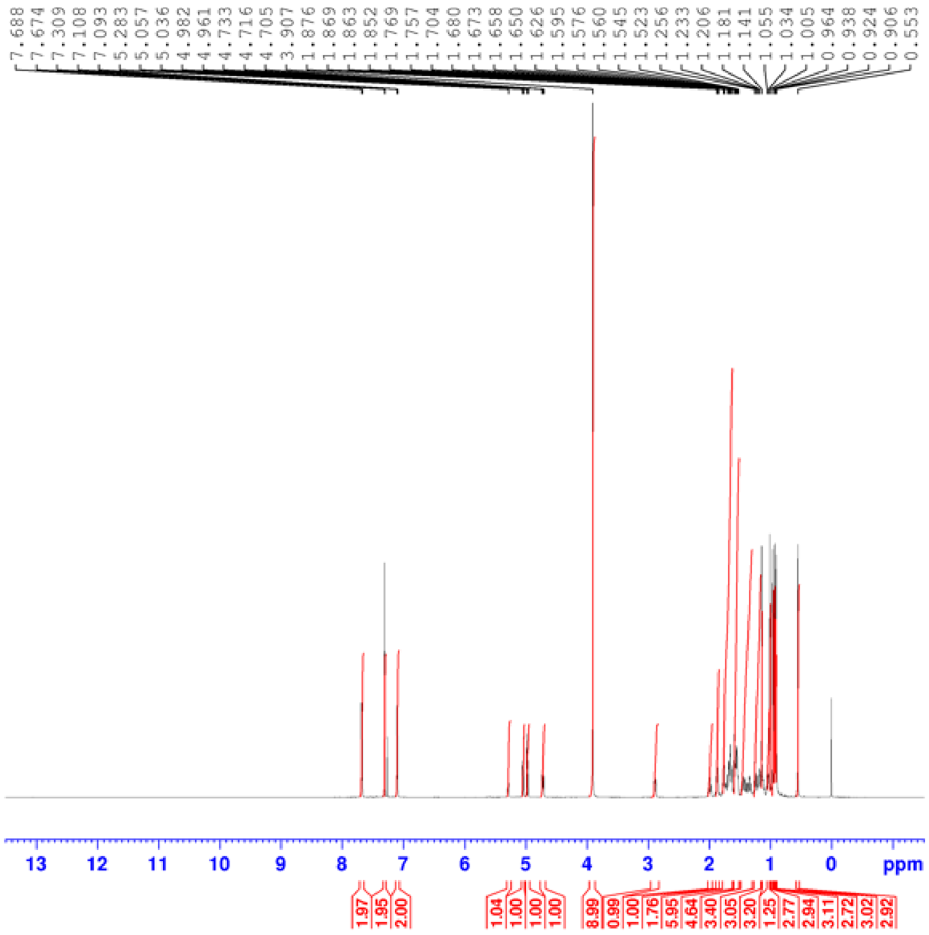

Figure S29: ^{13}C NMR Spectrum of **NOA-6**

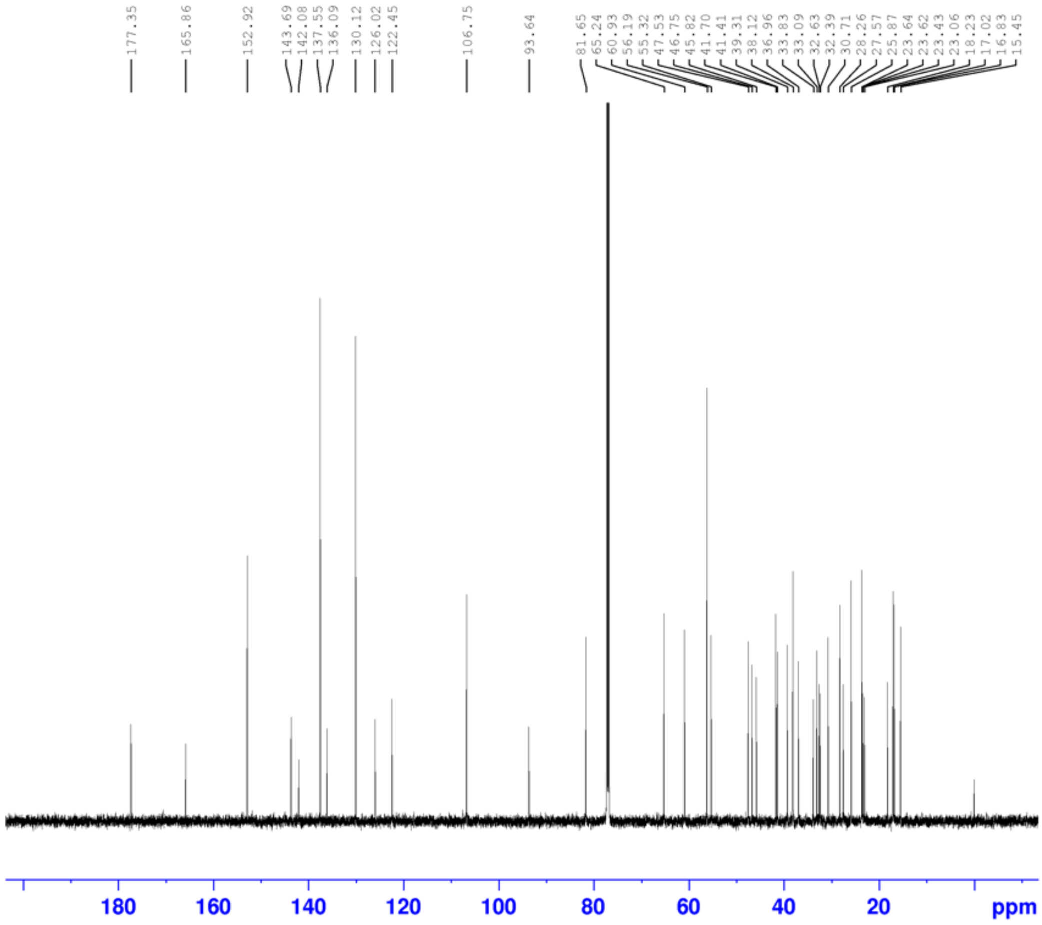

Figure S30: Mass Spectrum of **NOA-6**

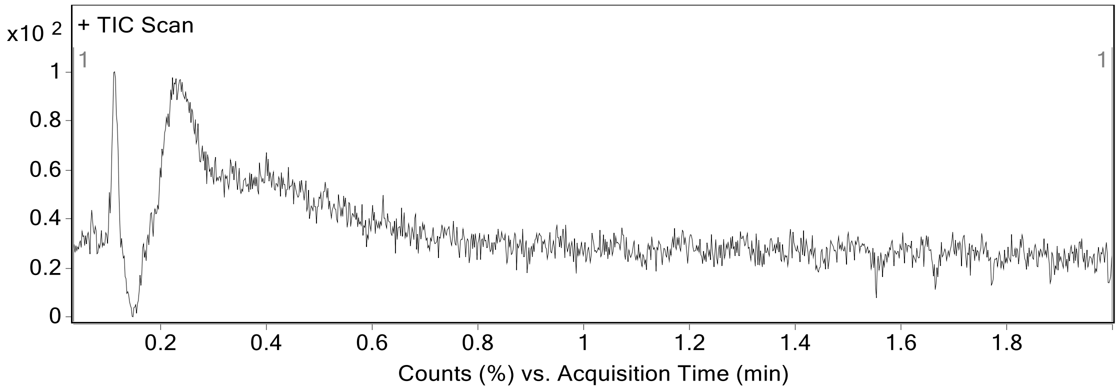

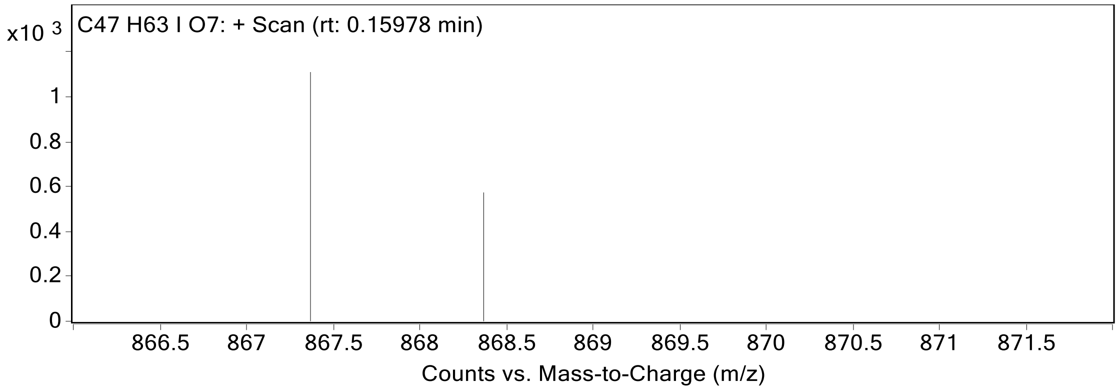

Formula Calculator Results

Formula	Best	Mass	Tgt Mass	Diff (ppm)	Ion Species	Score
C47 H63 I O7	TRUE	866,36101	866,36185	-0,97	C47 H64 I O7	61,3

Figure S31: ^{1}H NMR Spectrum of **NOA-7**

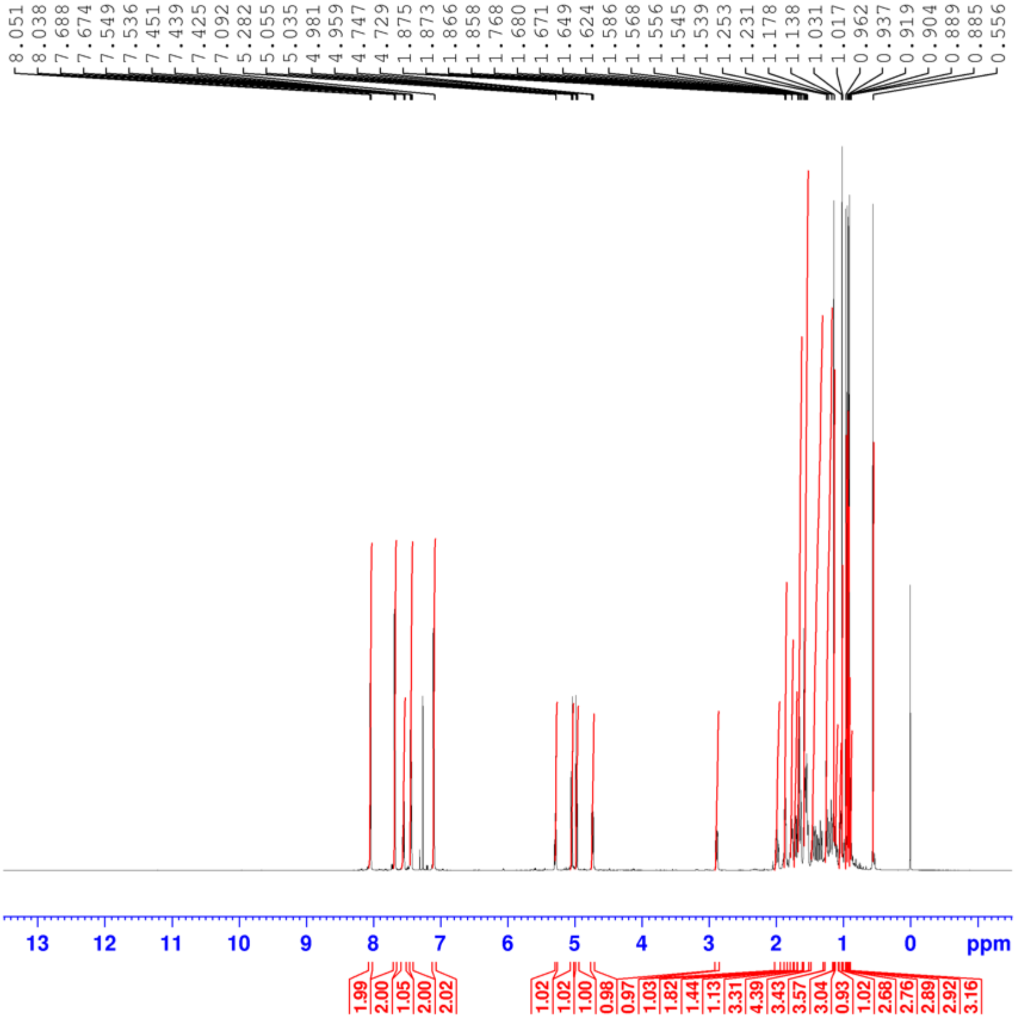

Figure S32: ^{13}C NMR Spectrum of **NOA-7**

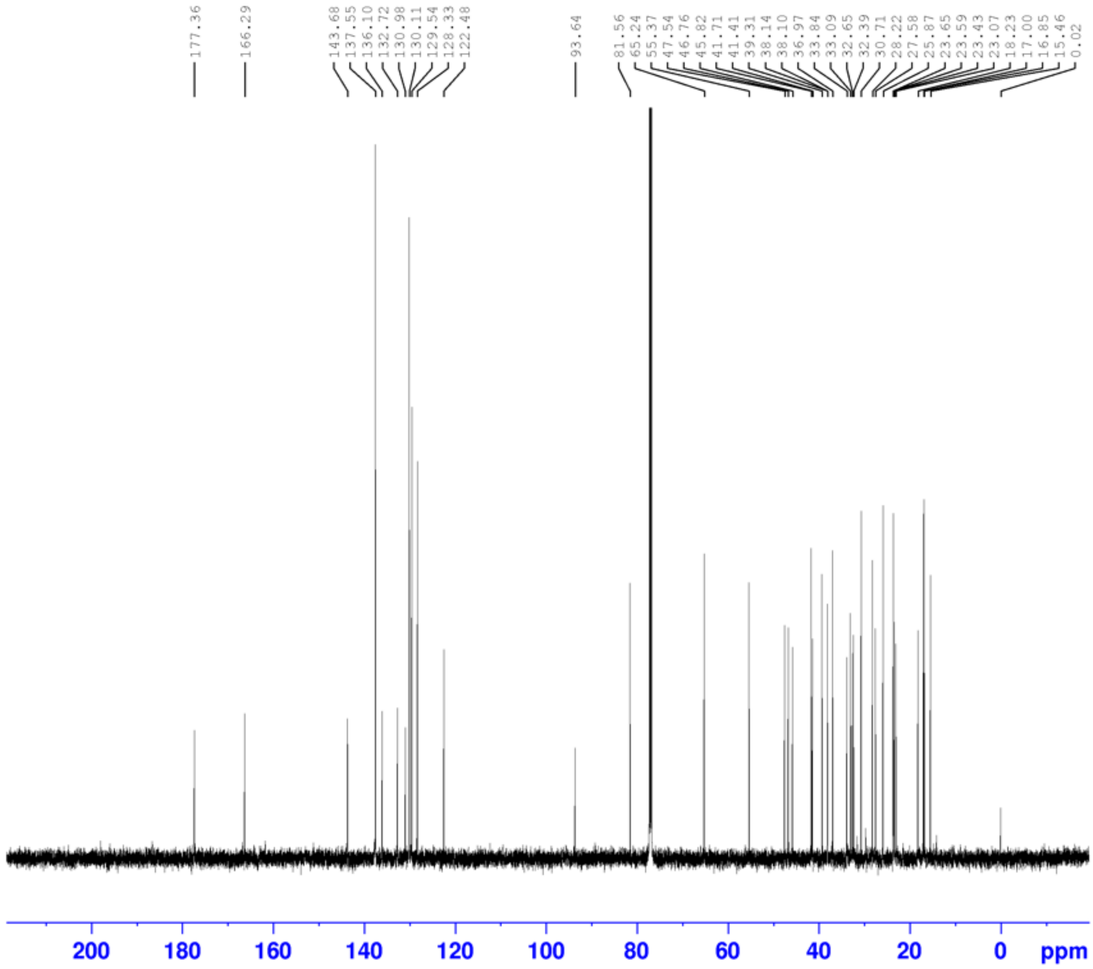

Figure S33: Mass Spectrum of **NOA-7**

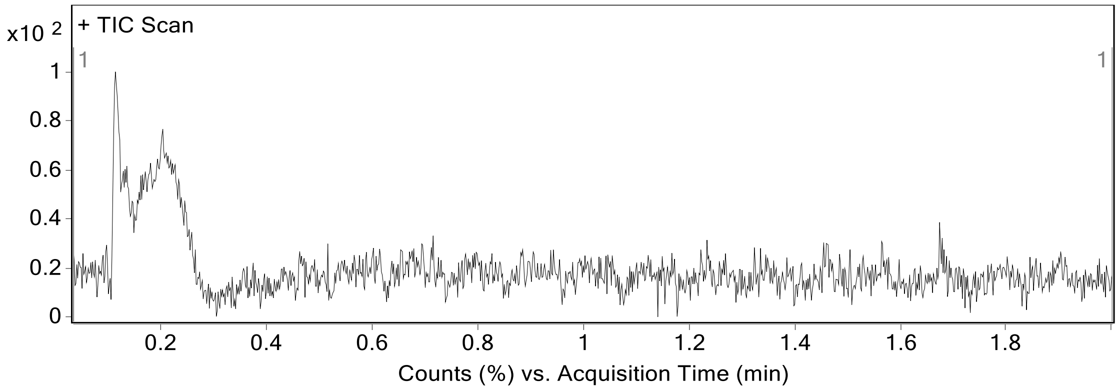

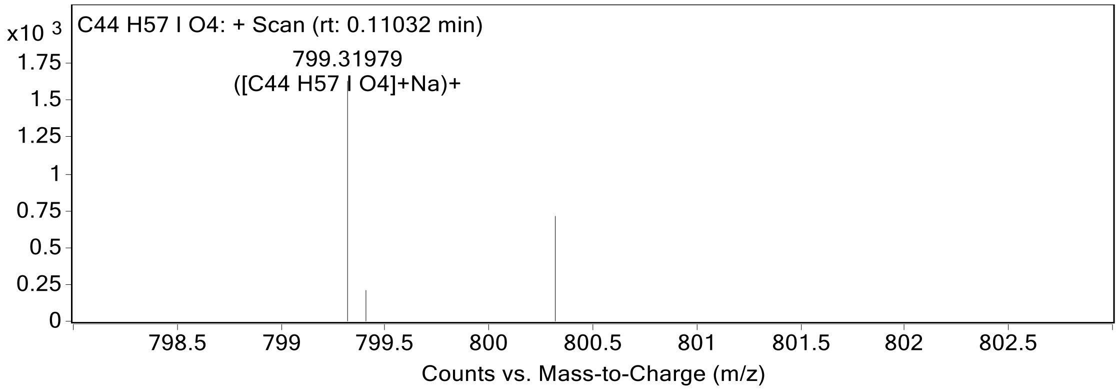

Formula Calculator Results

Formula	Best	Mass	Tgt Mass	Diff (ppm)	Ion Species	Score
C44 H57 I O4	TRUE	776,32929	776,33015	-1,11	C44 H57 I Na O4	63,32

Figure S34: ^{1}H NMR Spectrum of **NOA-8**

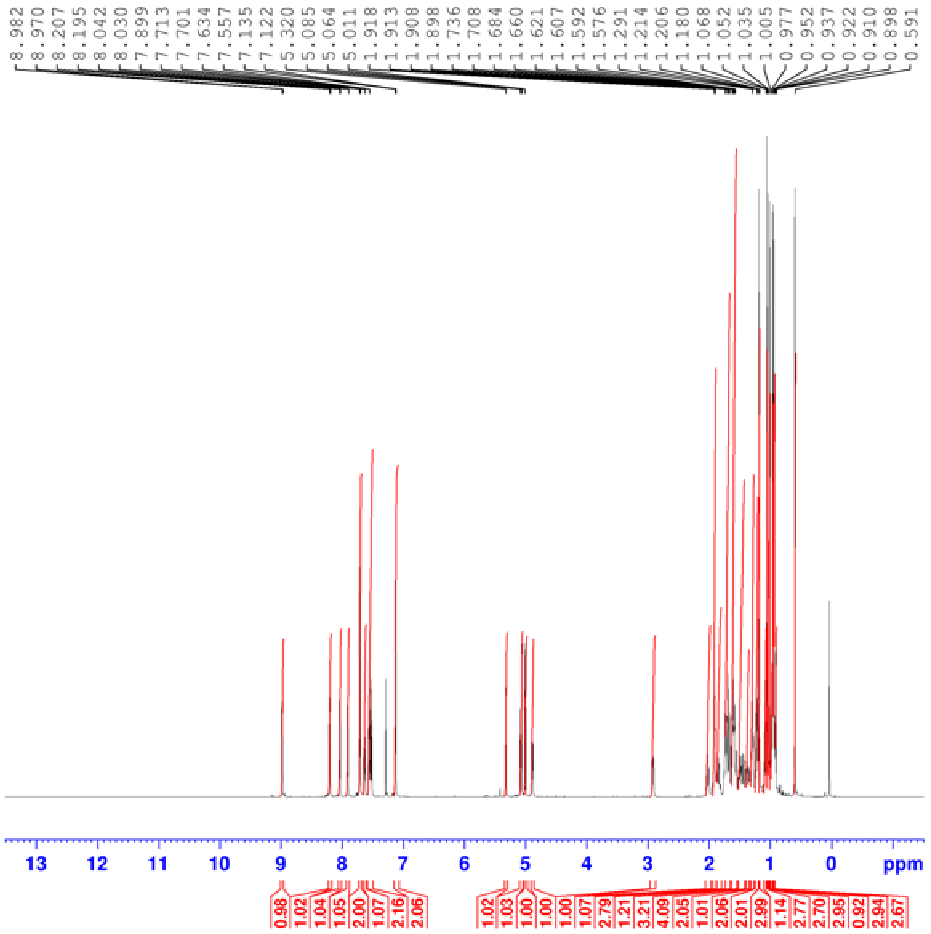

Figure S35: ^{13}C NMR Spectrum of **NOA-8**

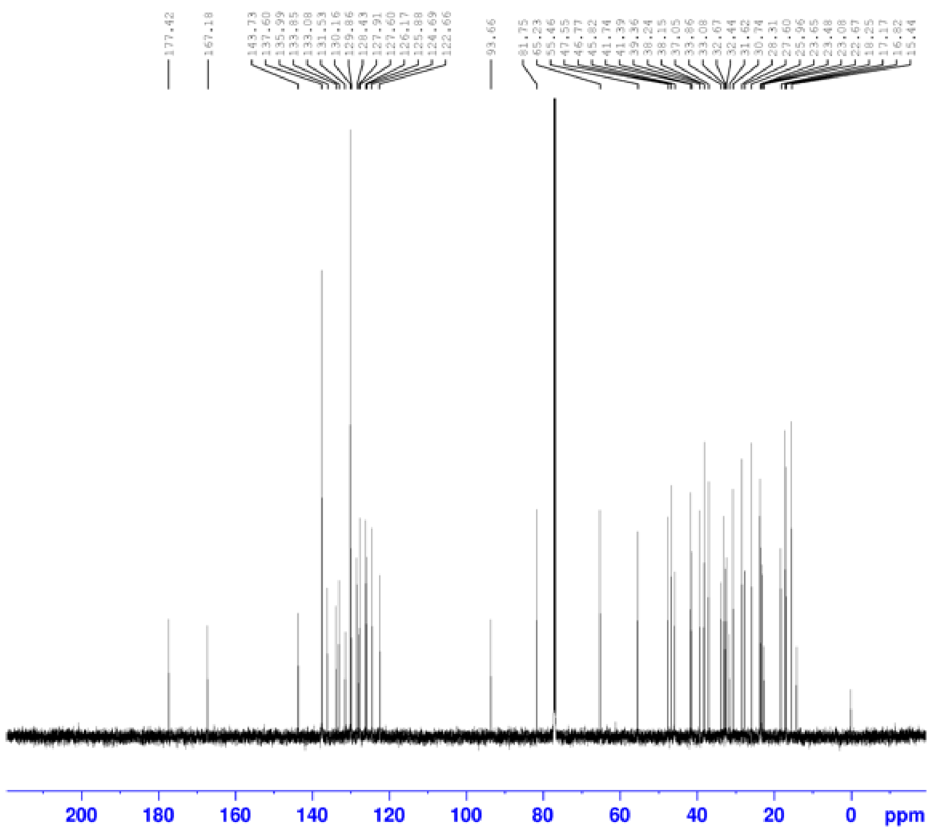

Figure S36: Mass Spectrum of **NOA-8**

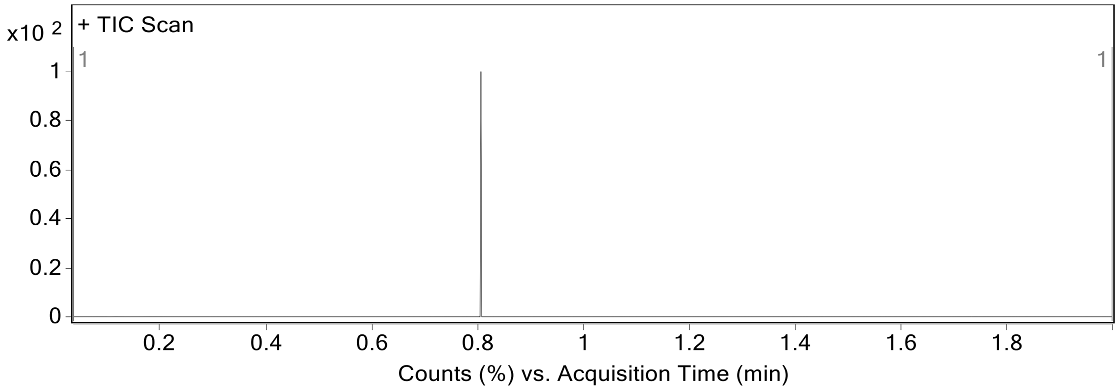

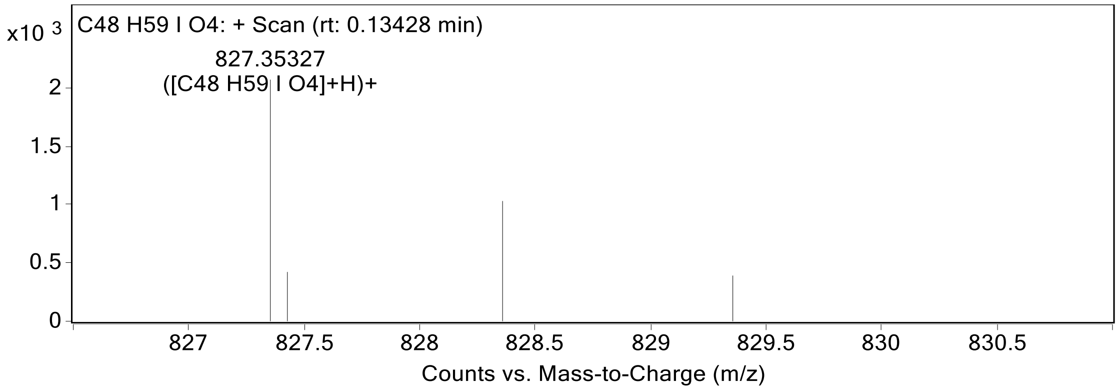

Formula Calculator Results

Formula	Best	Mass	Tgt Mass	Diff (ppm)	Ion Species	Score
C48 H59 I O4	TRUE	826,34622	826,3458	0,5	C48 H60 I O4	89,52

Figure S37: ^{1}H NMR Spectrum of **NOA-9**

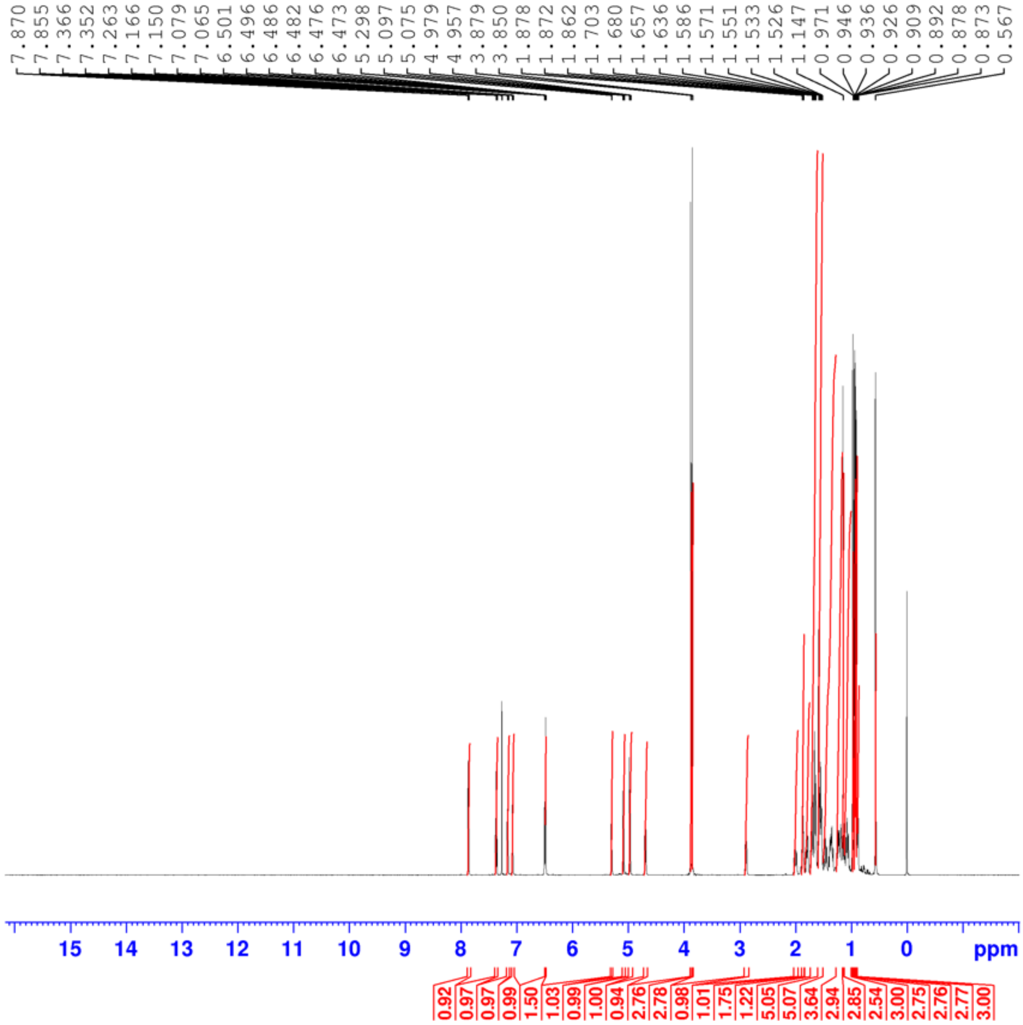

Figure S38: ^{13}C NMR Spectrum of **NOA-9**

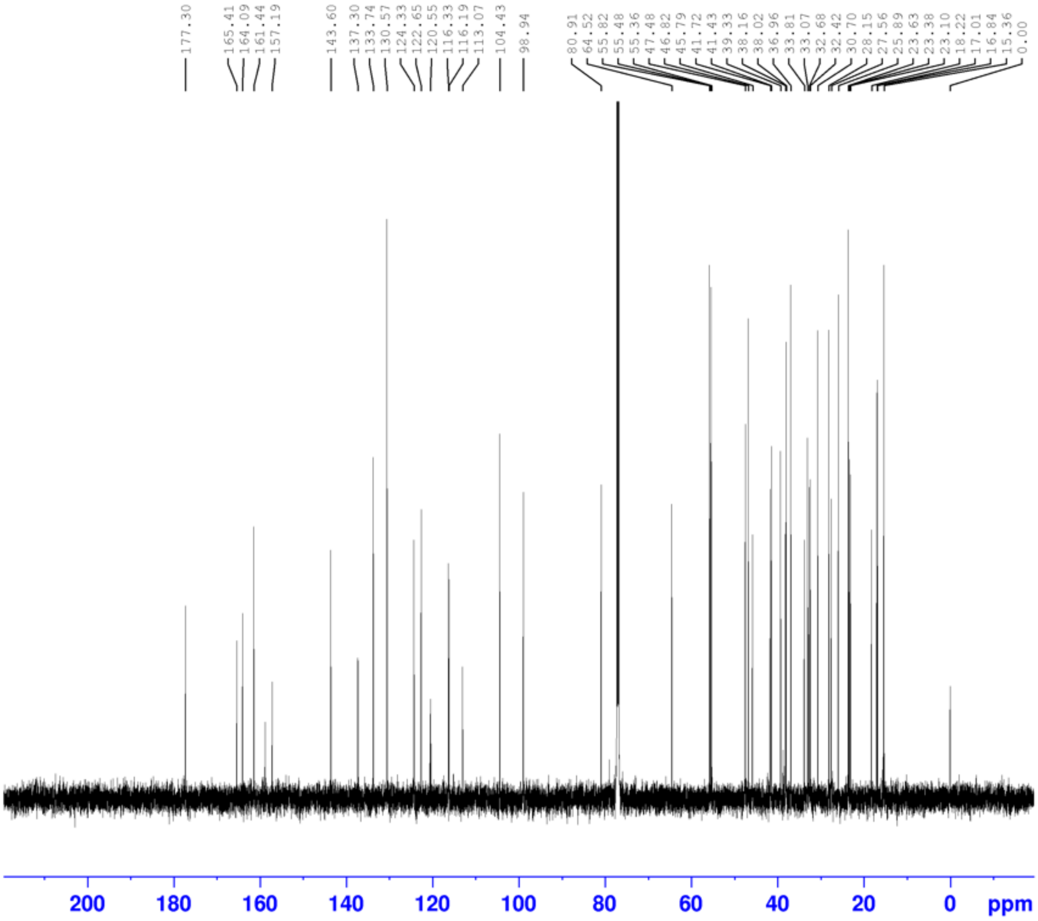

Figure S39: Mass Spectrum of **NOA-9**

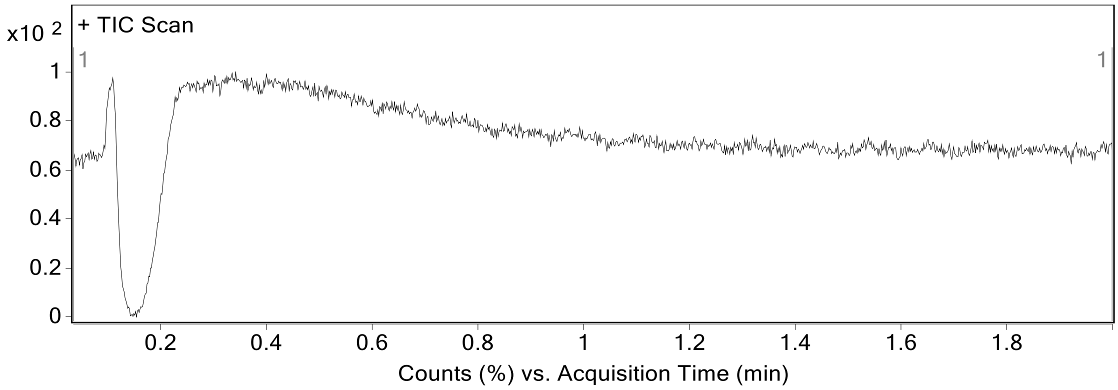

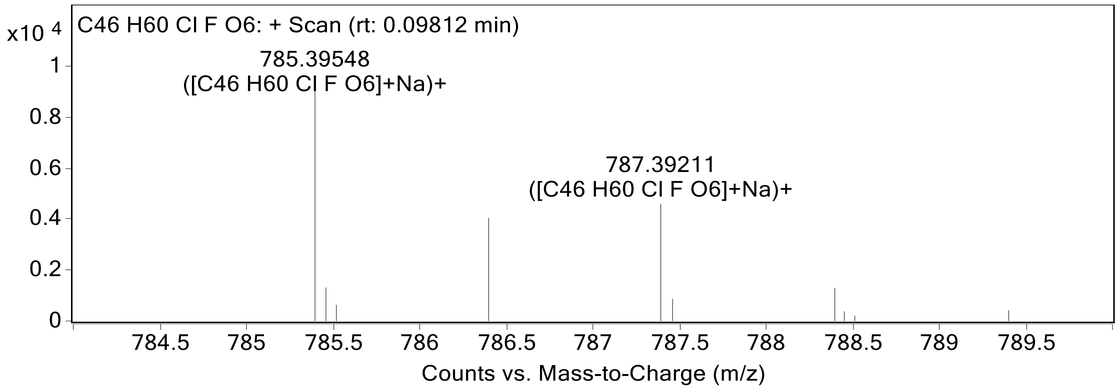

Formula Calculator Results

Formula	Best	Mass	Tgt Mass	Diff (ppm)	Ion Species	Score
C46 H60 Cl F O6	TRUE	762,40533	762,40625	-1,21	C46 H60 Cl F Na O6	89,51

Figure S40: ^{1}H NMR Spectrum of **NOA-10**

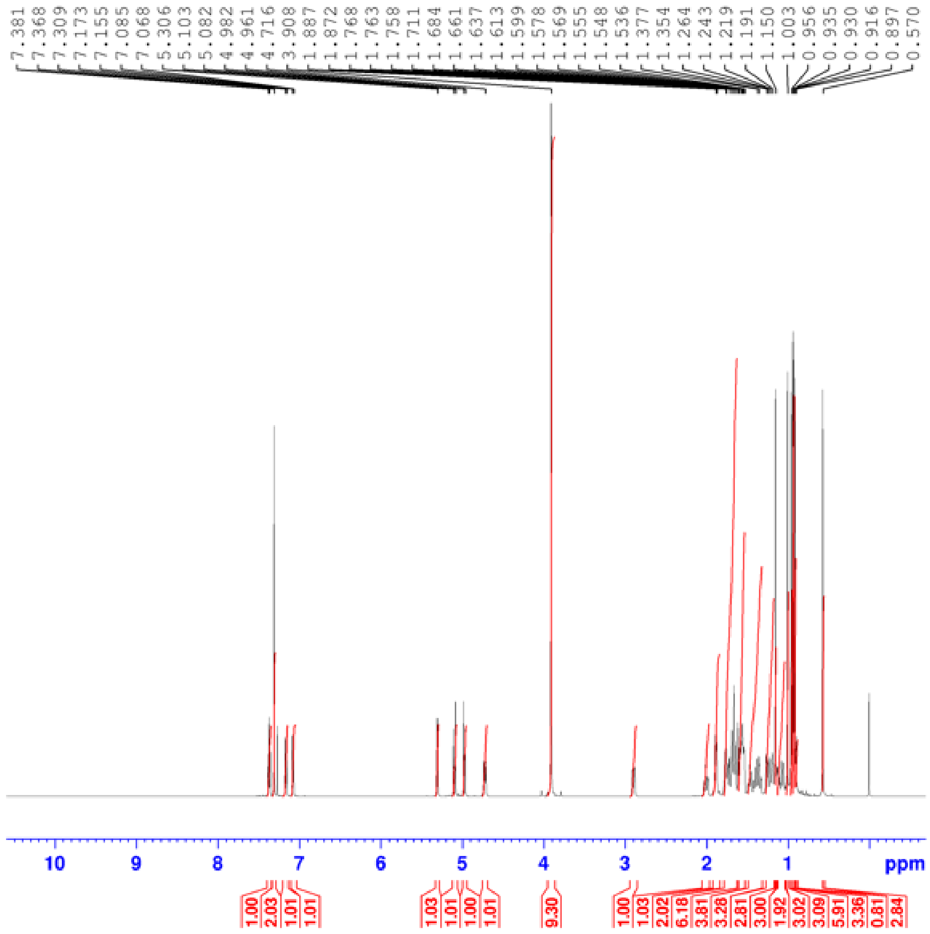

Figure S41: ^{13}C NMR Spectrum of **NOA-10**

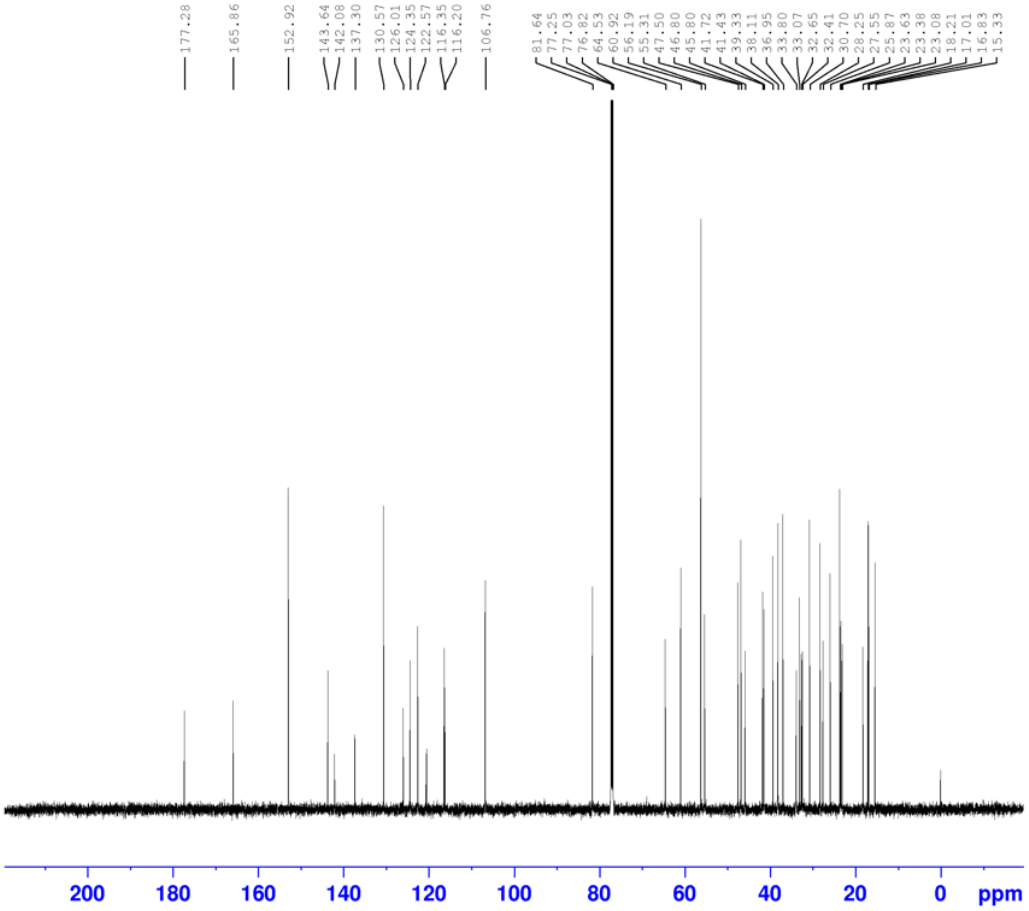

Figure S42: Mass Spectrum of **NOA-10**

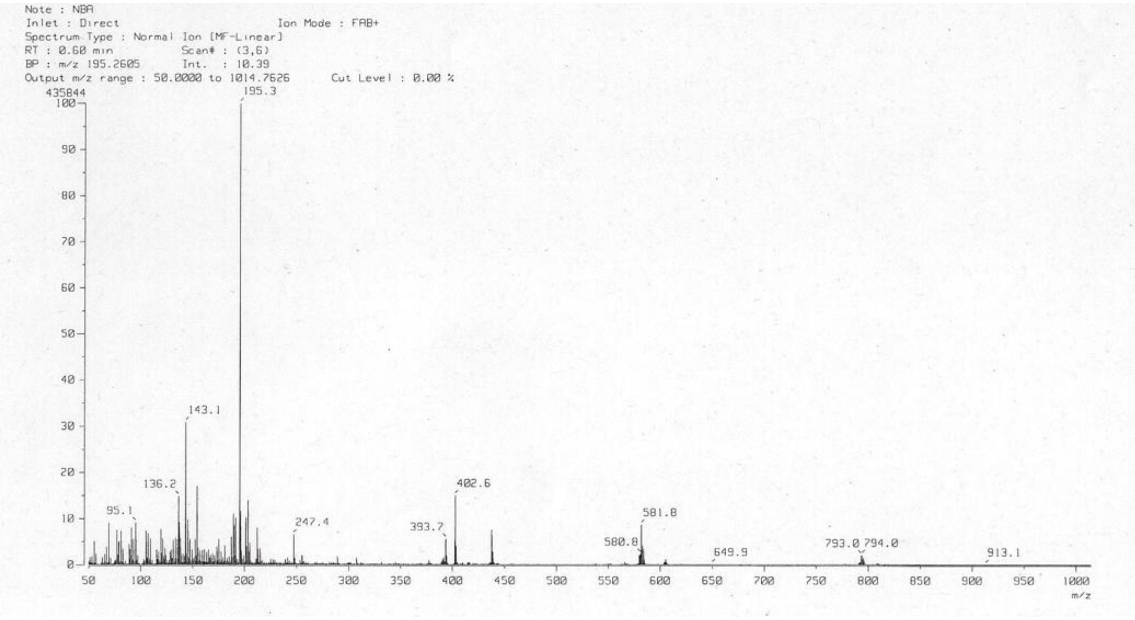

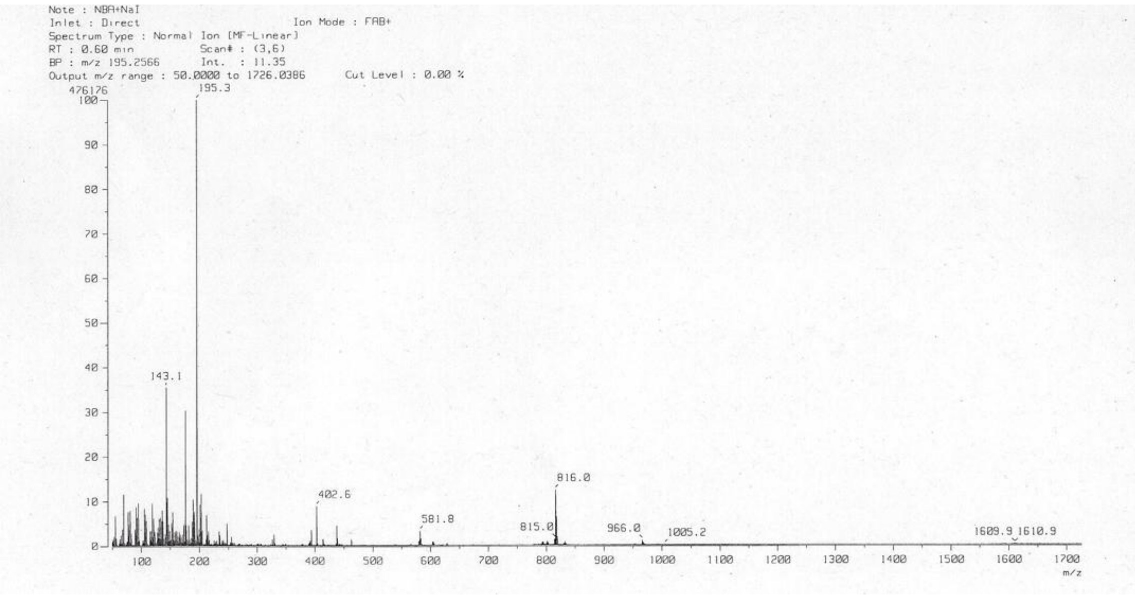

Figure S43: ^{1}H NMR Spectrum of **NOA-11**

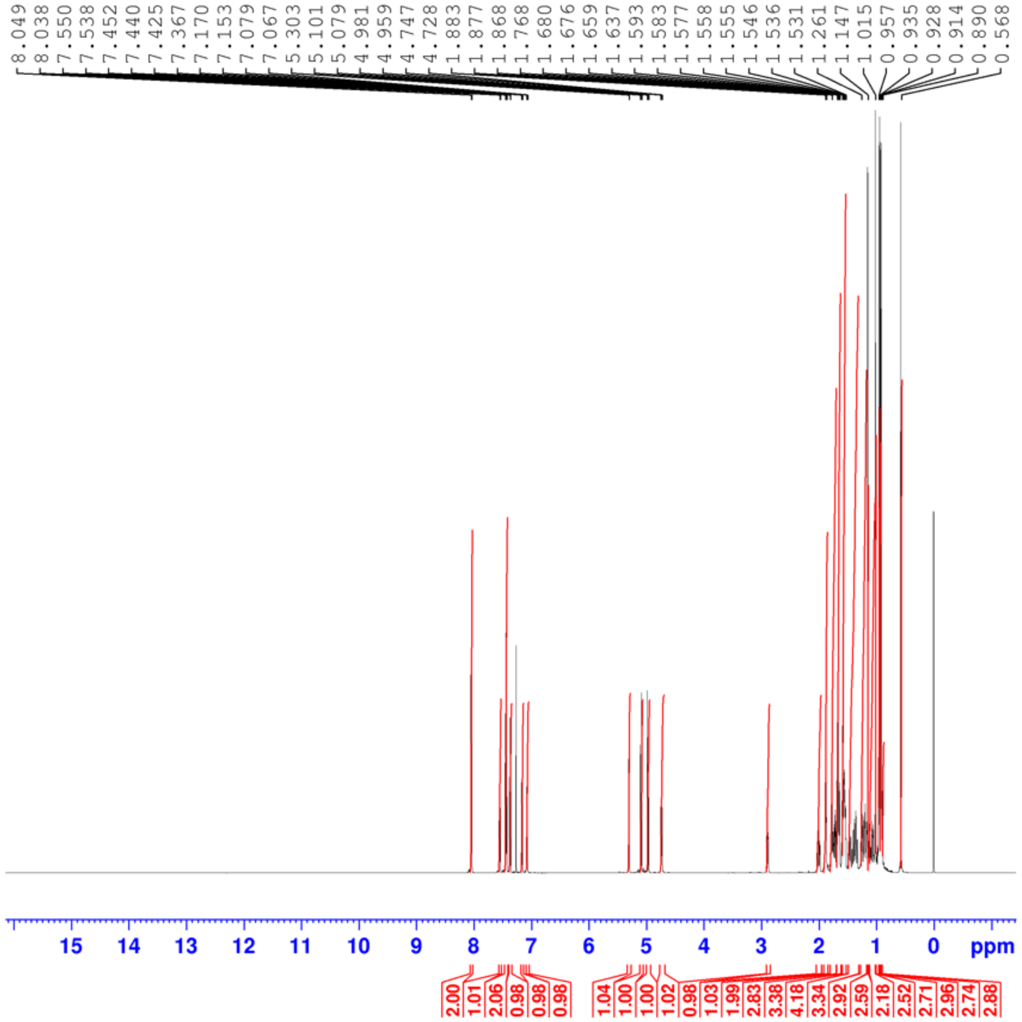

Figure S44: ^{13}C NMR Spectrum of **NOA-11**

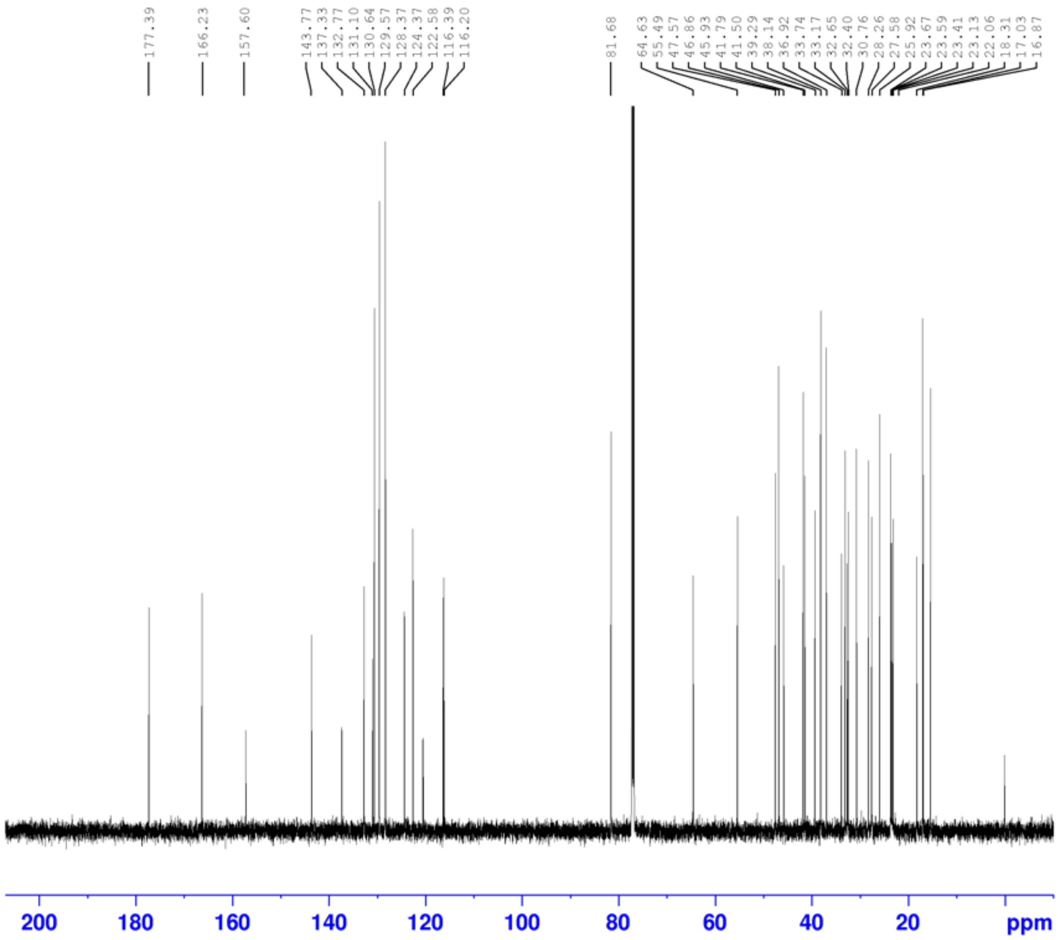

Figure S45: Mass Spectrum of **NOA-11**

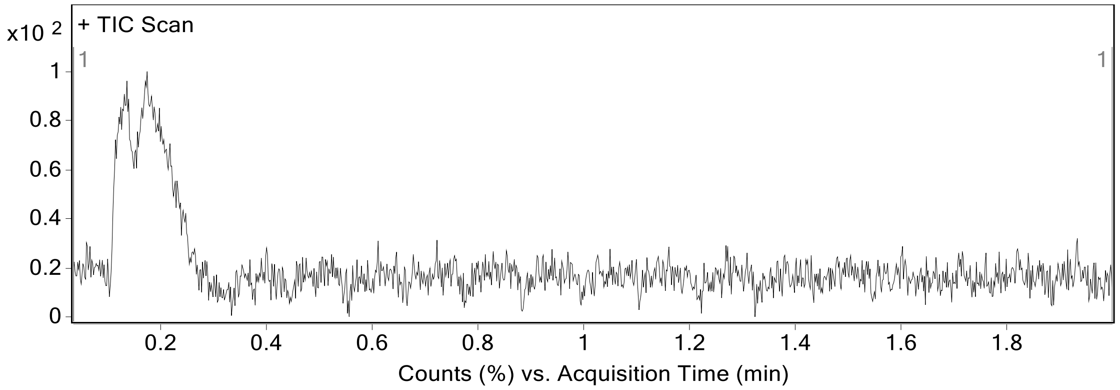

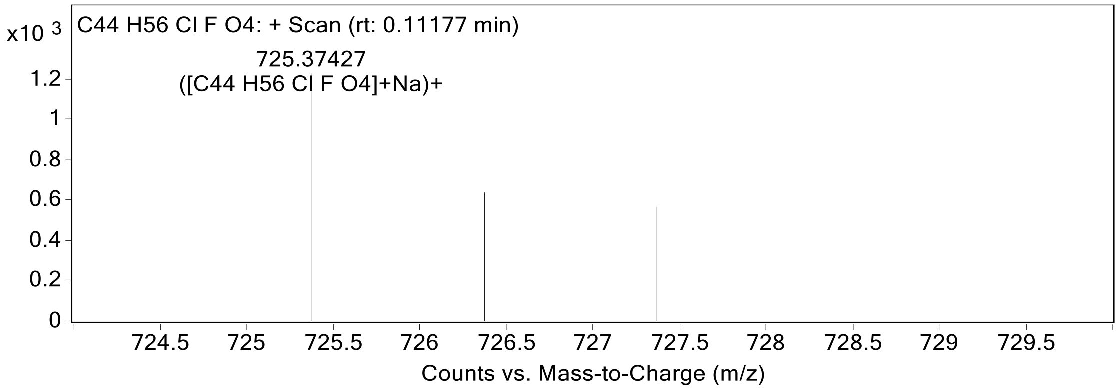

Formula Calculator Results

Formula	Best	Mass	Tgt Mass	Diff (ppm)	Ion Species	Score
C44 H56 Cl F O4	TRUE	702,38395	702,38512	-1,66	C44 H56 Cl F Na O4	72,81

Figure S46: ^{1}H NMR Spectrum of **NOA-12**

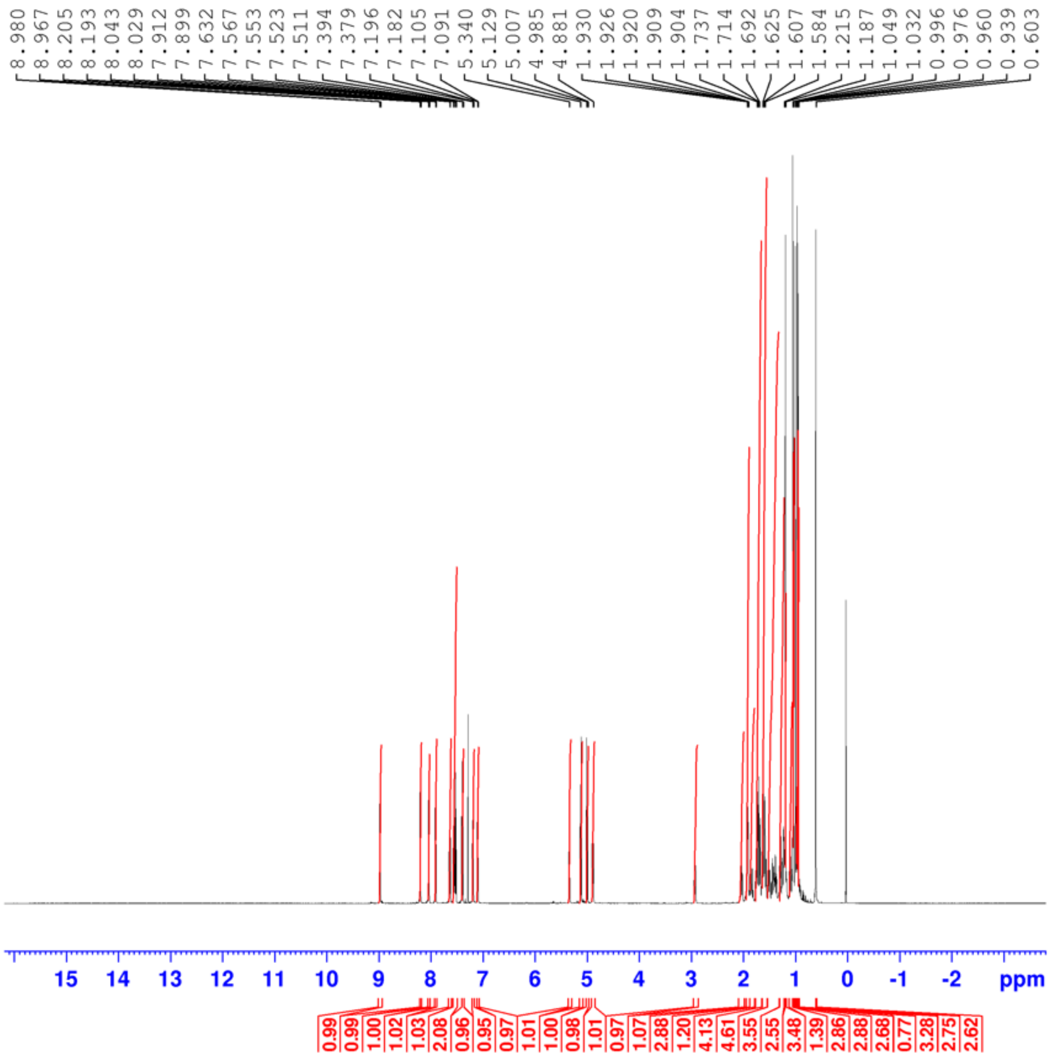

Figure S47: ^{13}C NMR Spectrum of **NOA-12**

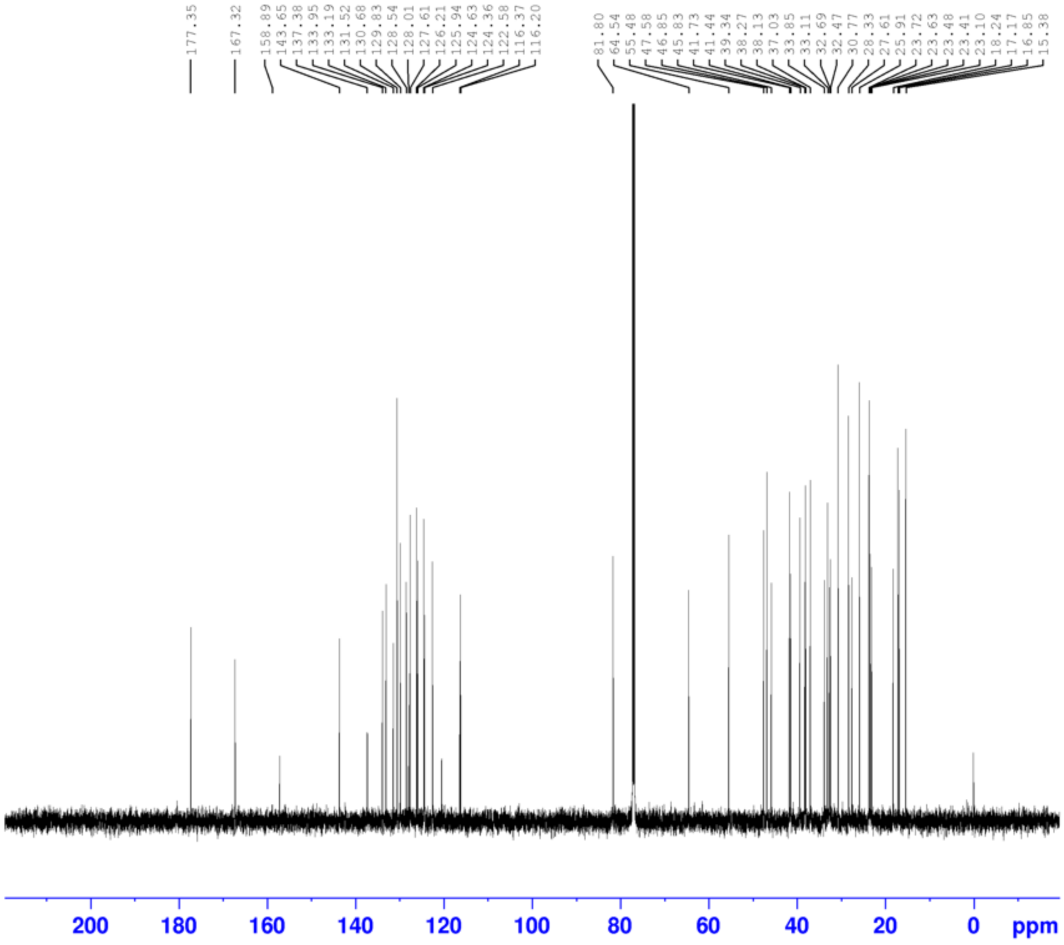

Figure S48: Mass Spectrum of **NOA-12**

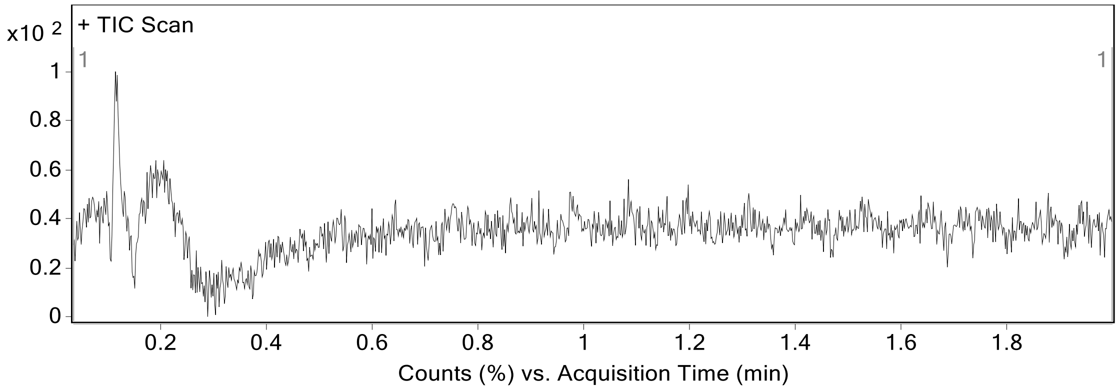

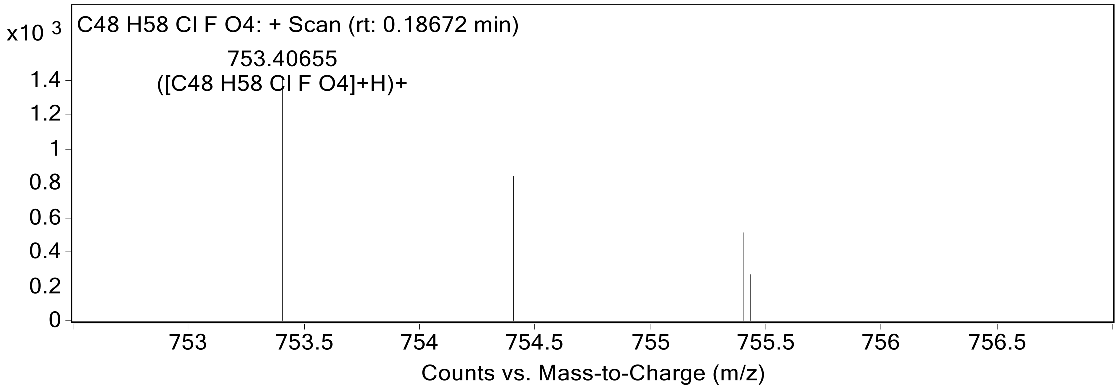

Formula Calculator Results

Formula	Best	Mass	Tgt Mass	Diff (ppm)	Ion Species	Score
C48 H58 Cl F O4	TRUE	752,39755	752,40077	-4,28	C48 H59 Cl F O4	56,12

Figure S49: ^{1}H NMR Spectrum of **NOA-13**

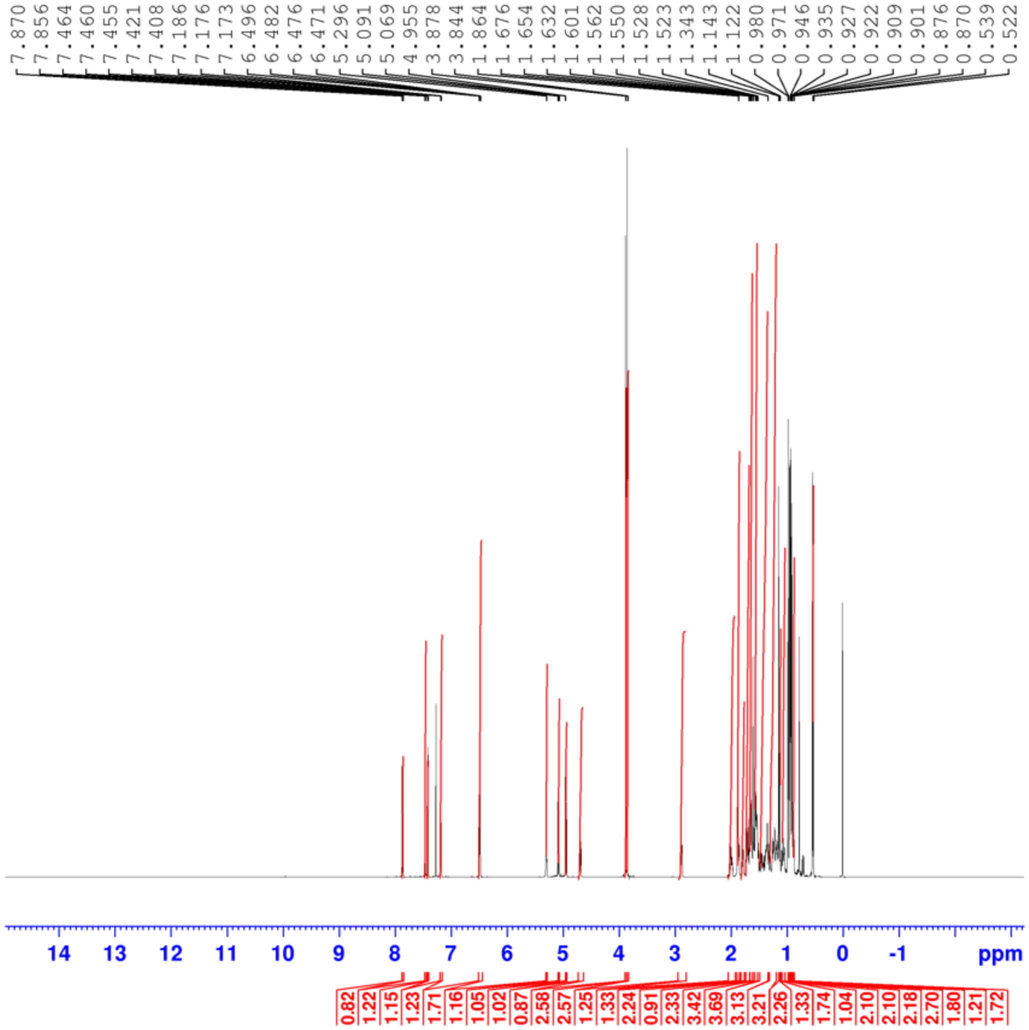

Figure S50: ^{13}C NMR Spectrum of **NOA-13**

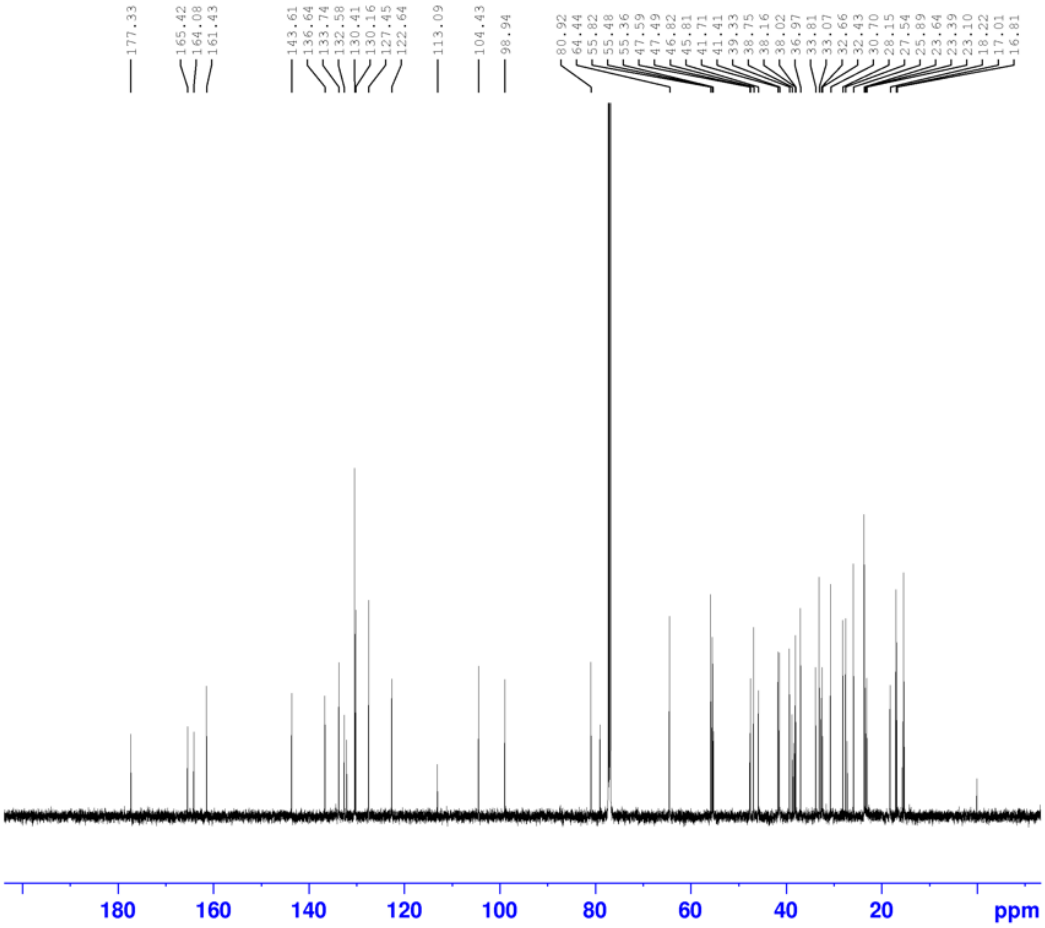

Figure S51: Mass Spectrum of **NOA-13**

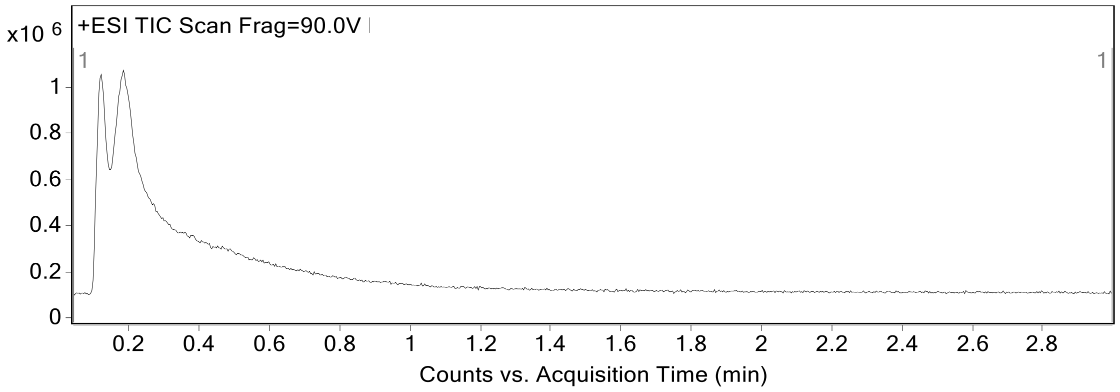

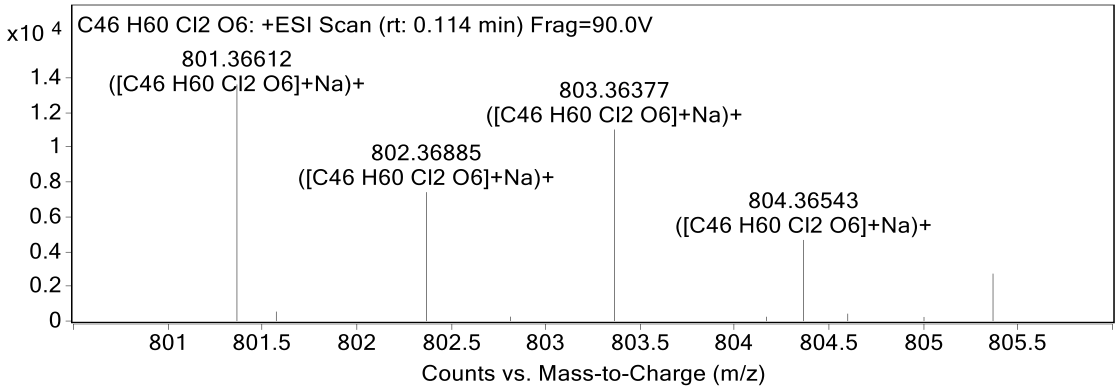

Formula Calculator Results

Formula	Best	Mass	Tgt Mass	Diff (ppm)	Ion Species	Score
C46 H60 Cl2 O6	TRUE	778,37646	778,3767	-0,3	C46 H60 Cl2 Na O6	96,64

Figure S52: ^{1}H NMR Spectrum of **NOA-14**

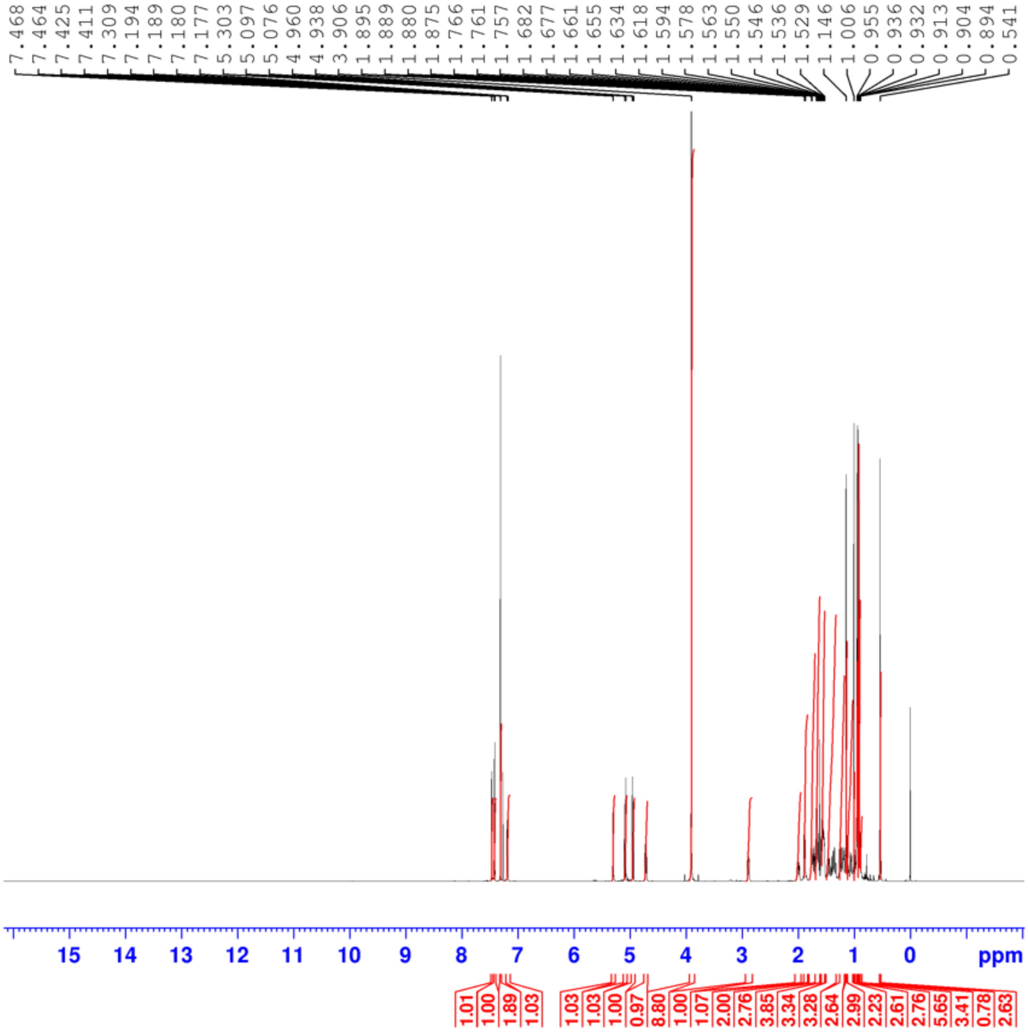

Figure S53: ^{13}C NMR Spectrum of **NOA-14**

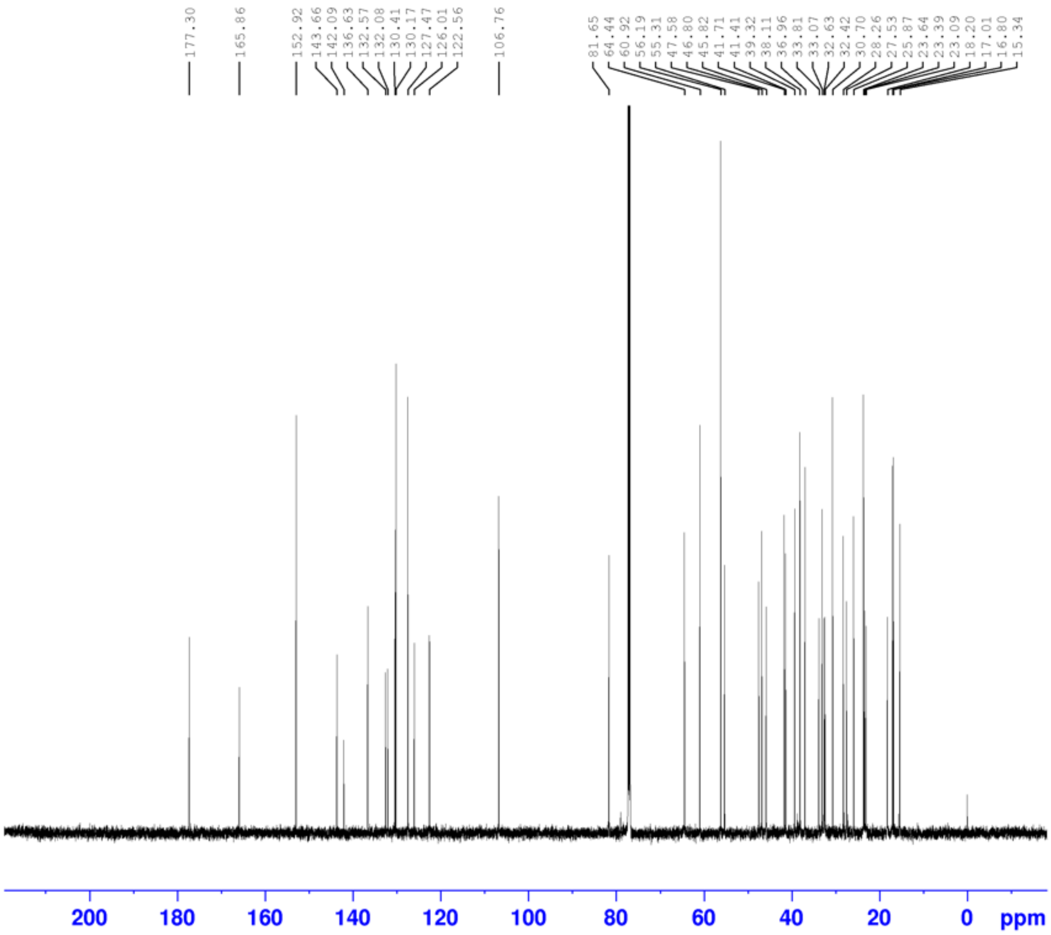

Figure S54: Mass Spectrum of **NOA-14**

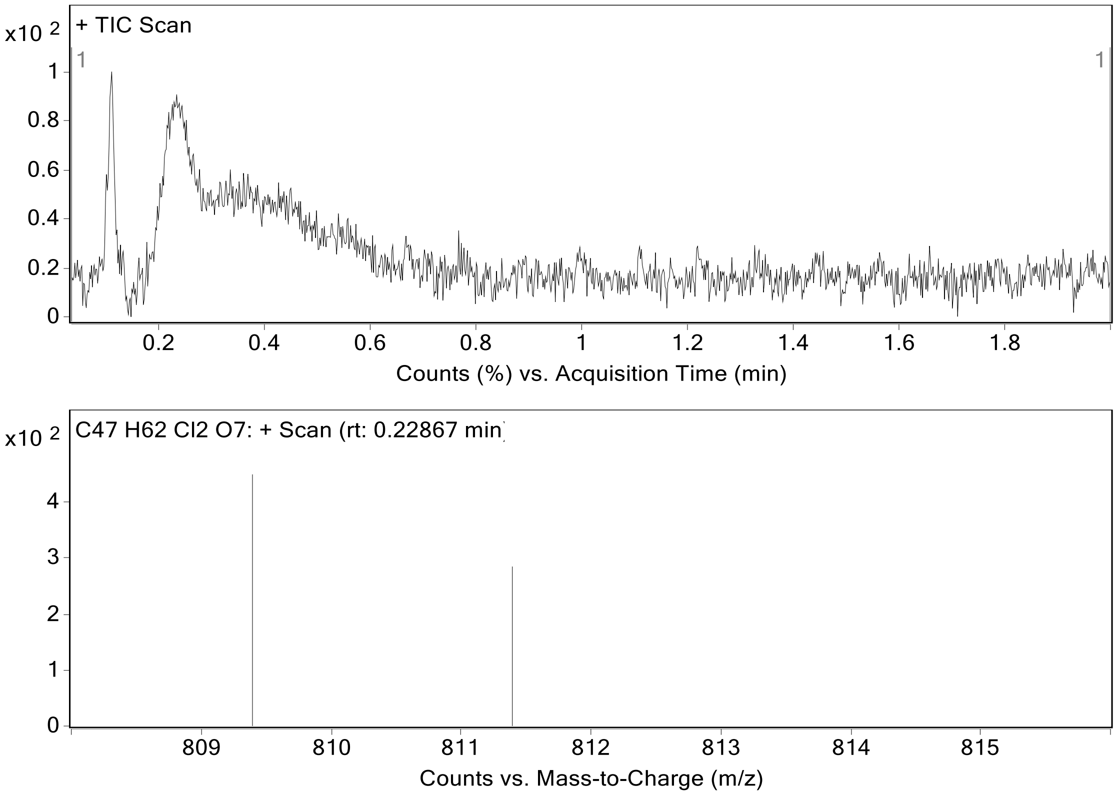

Formula Calculator Results

Formula	Best	Mass	Tgt Mass	Diff (ppm)	Ion Species	Score
C47 H62 Cl2 O7	TRUE	808,38739	808,38726	0,16	C47 H63 Cl2 O7	47,6

Figure S55: ^{1}H NMR Spectrum of **NOA-15**

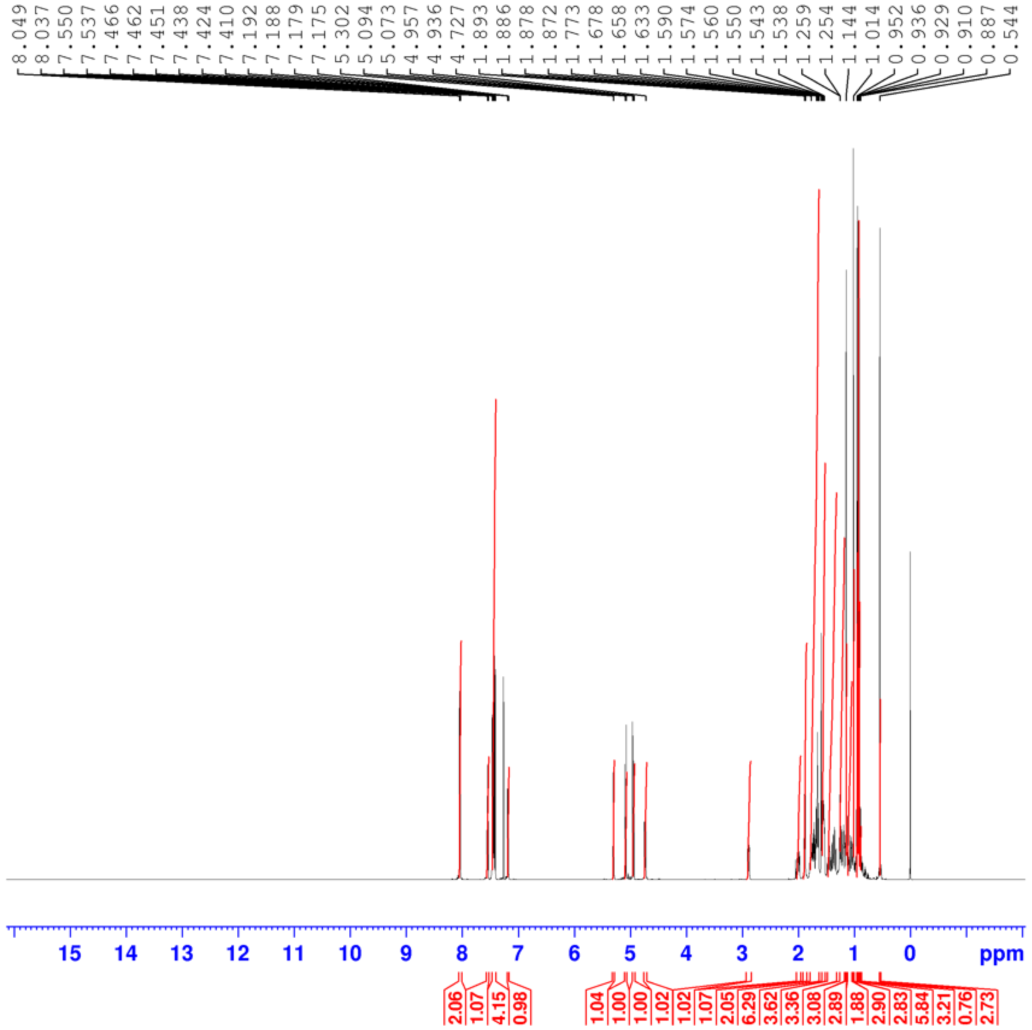

Figure S56: ^{13}C NMR Spectrum of **NOA-15**

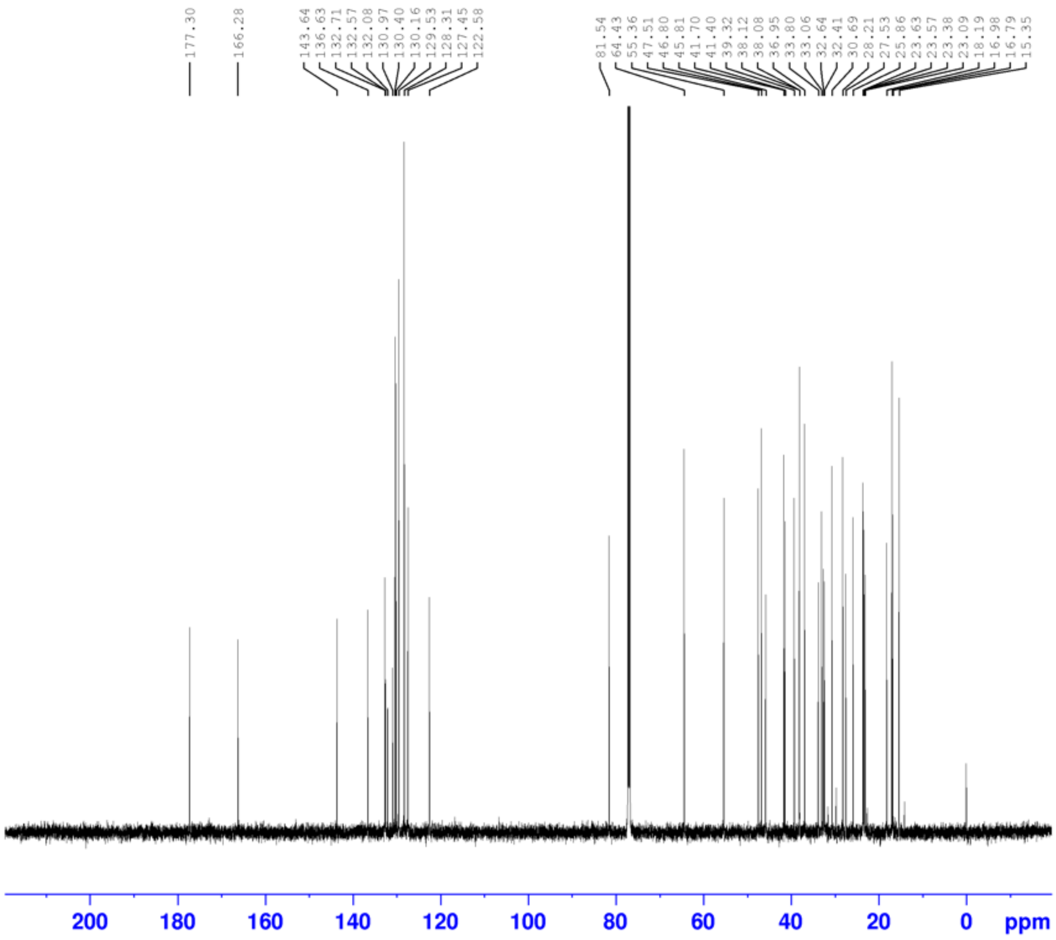

Figure S57: Mass Spectrum of **NOA-15**

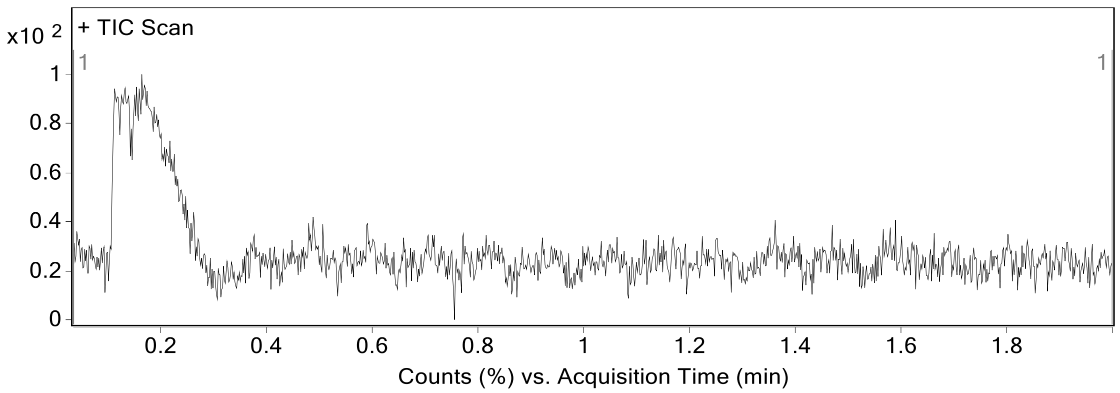

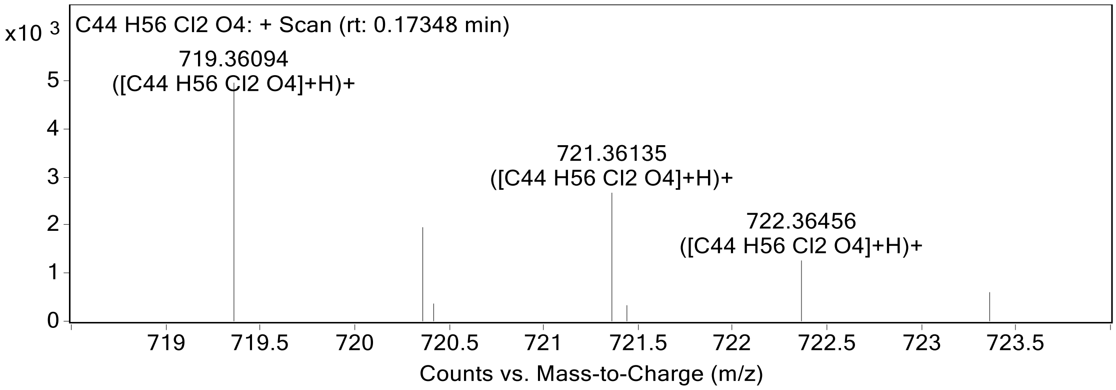

Formula Calculator Results

Formula	Best	Mass	Tgt Mass	Diff (ppm)	Ion Species	Score
C44 H56 Cl2 O4	TRUE	718,35412	718,35557	-2,01	C44 H57 Cl2 O4	83,05

Figure S58: ^{1}H NMR Spectrum of **NOA-16**

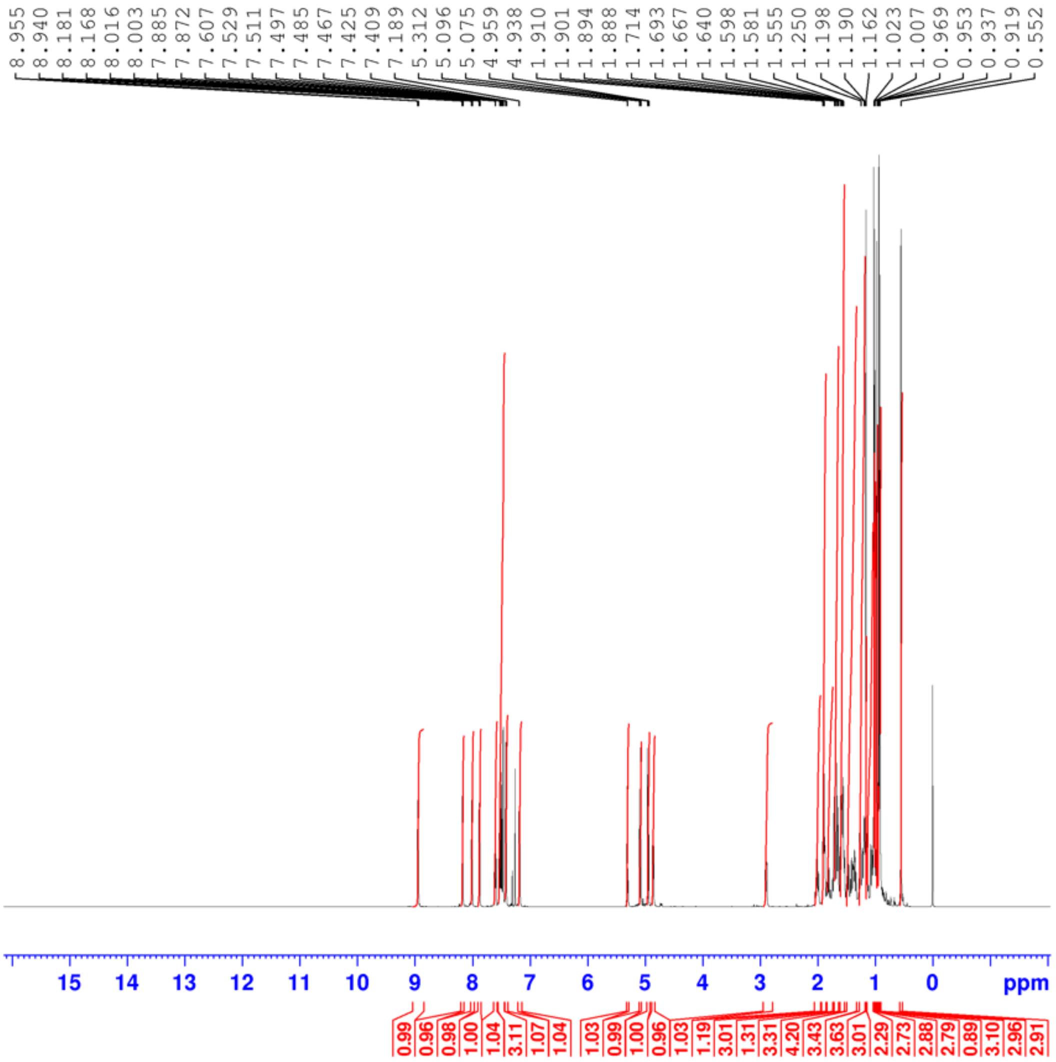

Figure S59: ^{13}C NMR Spectrum of **NOA-16**

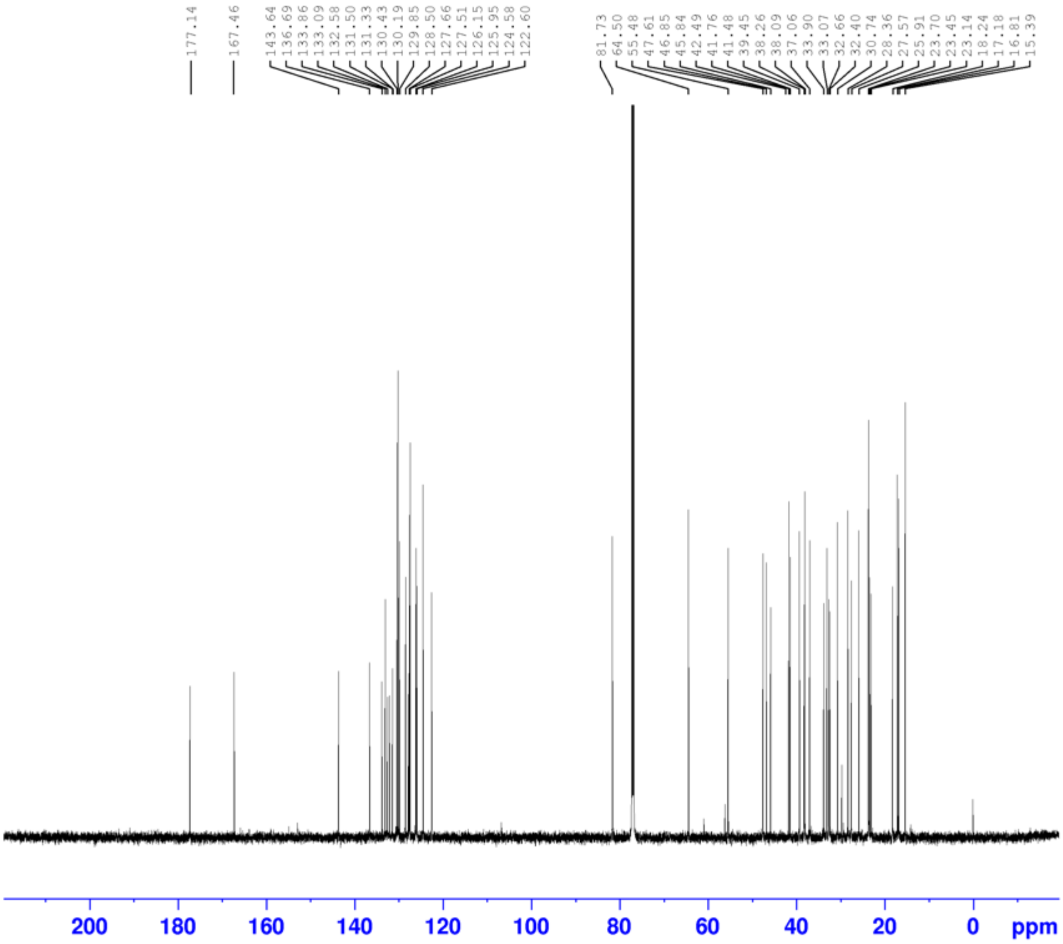

Figure S60: Mass Spectrum of **NOA-16**

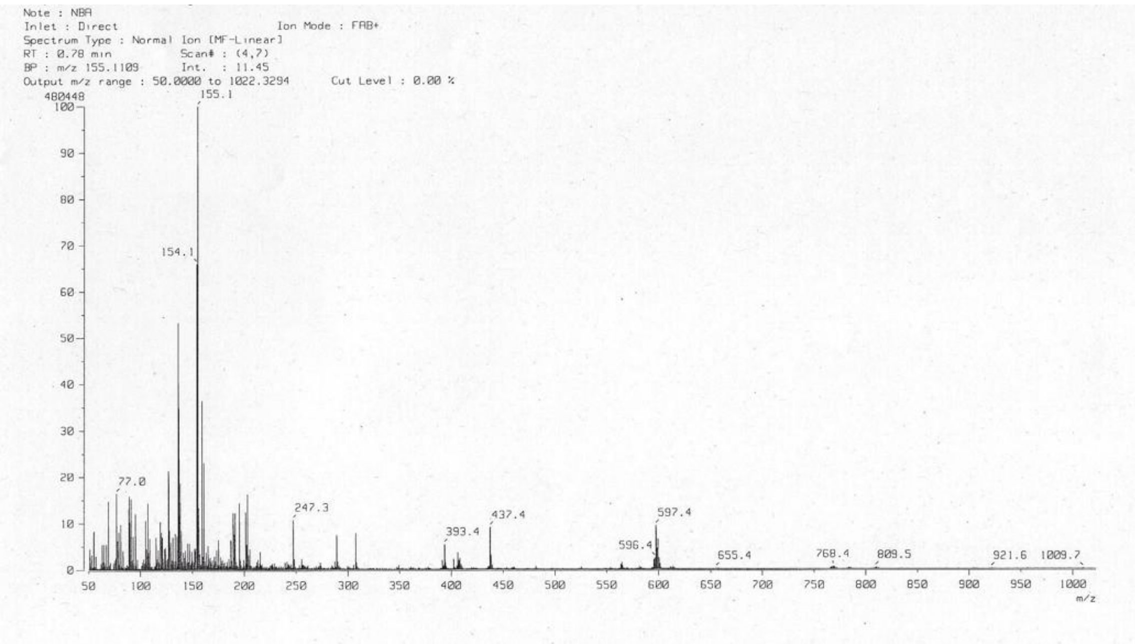

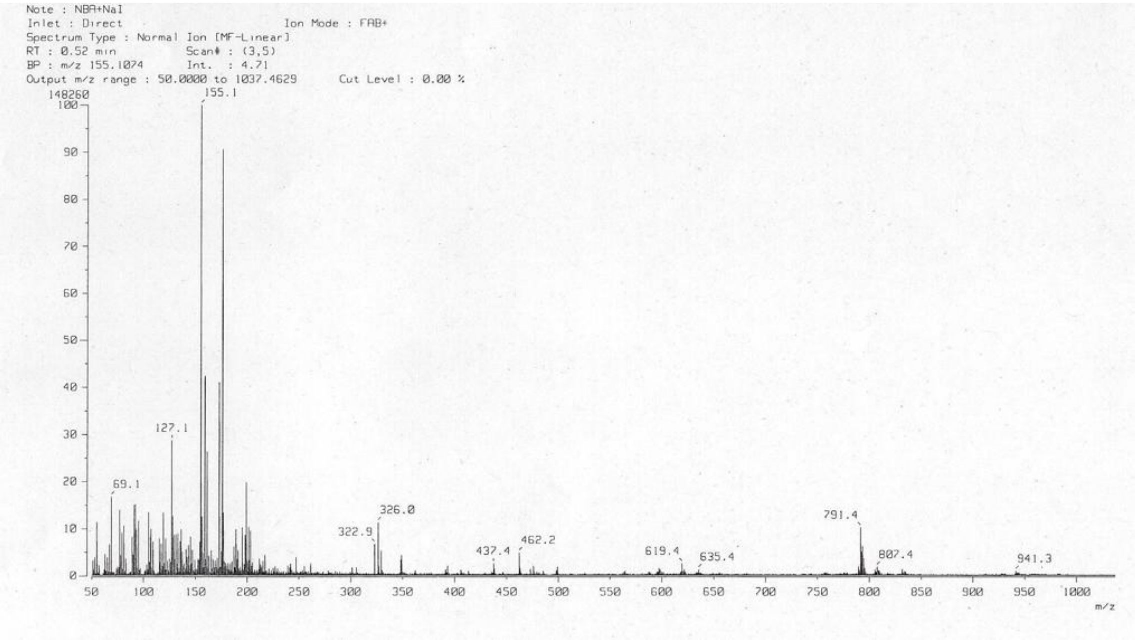

Supplement: Supplementary file 1 [file life-16-01335-s001.zip › life-4472618-supplementary.pdf]
